# HCN channels in the mammalian cochlea: Expression pattern, subcellular location, and age‐dependent changes

**DOI:** 10.1002/jnr.24754

**Published:** 2020-11-12

**Authors:** Maria Luque, Anneliese Schrott‐Fischer, Jozsef Dudas, Elisabeth Pechriggl, Erich Brenner, Helge Rask‐Andersen, Wei Liu, Rudolf Glueckert

**Affiliations:** ^1^ Department of Otorhinolaryngology Medical University of Innsbruck Innsbruck Austria; ^2^ Department of Anatomy, Histology & Embryology Division of Clinical & Functional Anatomy Medical University of Innsbruck Innsbruck Austria; ^3^ Department of Surgical Sciences, Head and Neck Surgery Section of Otolaryngology Uppsala University Hospital Uppsala Sweden; ^4^ Tirol Kliniken University Clinics Innsbruck Innsbruck Austria

**Keywords:** auditory development, auditory neuron diversity, axon initial segment, HCN channels, prestin, RRID:AB_90725, RRID:AB_2039906, RRID:AB_2302038, RRID:AB_2313584, RRID:AB_2313726, RRID:AB_2336419, RRID:AB_2336420, RRID:AB_2336790, RRID:AB_2340477, RRID:AB_2340452, RRID:AB_2340593, RRID:AB_2341028, RRID:AB_2617143, RRID:AB_2756625, RRID:AB_2756742, RRID:SCR_002865, RRID:SCR_013652, RRID:SCR_014823, sound coding, spiral ganglion neurons, voltage gated

## Abstract

Neuronal diversity in the cochlea is largely determined by ion channels. Among voltage‐gated channels, hyperpolarization‐activated cyclic nucleotide‐gated (HCN) channels open with hyperpolarization and depolarize the cell until the resting membrane potential. The functions for hearing are not well elucidated and knowledge about localization is controversial. We created a detailed map of subcellular location and co‐expression of all four HCN subunits across different mammalian species including CBA/J, C57Bl/6N, Ly5.1 mice, guinea pigs, cats, and human subjects. We correlated age‐related hearing deterioration in CBA/J and C57Bl/6N with expression levels of HCN1, −2, and −4 in individual auditory neurons from the same cohort. Spatiotemporal expression during murine postnatal development exposed HCN2 and HCN4 involvement in a critical phase of hair cell innervation. The huge diversity of subunit composition, but lack of relevant heteromeric pairing along the perisomatic membrane and axon initial segments, highlighted an active role for auditory neurons. Neuron clusters were found to be the hot spots of HCN1, −2, and −4 immunostaining. HCN channels were also located in afferent and efferent fibers of the sensory epithelium. Age‐related changes on HCN subtype expression were not uniform among mice and could not be directly correlated with audiometric data. The oldest mice groups revealed HCN channel up‐ or downregulation, depending on the mouse strain. The unexpected involvement of HCN channels in outer hair cell function where HCN3 overlaps prestin location emphasized the importance for auditory function. A better understanding may open up new possibilities to tune neuronal responses evoked through electrical stimulation by cochlear implants.

## Graphical abstract

All HCN channel subtypes were located at subcellular level in human and compared with other mammals such as guinea pig, cat, and three mouse strains. Changes in HCN expression levels were quantified in aging mice and murine postnatal development. The widespread expression and distinct localization emphasized the importance of HCN channels for sound coding and electrical stimulation with cochlear implants

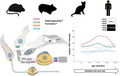

**Significance**

We provide a unique comparative analysis of subcellular HCN subunit localization across mammalian species, including humans. Spatiotemporal expression during postnatal development suggests its involvement as an active element in hair cell innervation. Lateral membrane staining in outer hair cells proposes association with cochlear amplification. There is no common regulation of expression level during aging across mouse strains. HCN hotspots in neuron clusters suggest functions for synchronization of timing cues. HCN expression patterns reflect auditory neuron heterogeneity that contributes to the enormous dynamic range and timing precision; consideration of HCN channels for electrical stimulation may enable new strategies.

## 1 INTRODUCTION

Auditory function essentially depends on the concerted interplay of the sensory cells and bipolar spiral ganglion neurons (SGNs) that convey receptor potential changes to ascending auditory pathways. Although most computational processing of auditory information occurs in the central nervous system (CNS), SGNs are more than point‐to‐point wires that route sound information to the brainstem. From eight to 20 of these bipolar neurons innervate a single inner hair cell (IHC) (Nayagam et al., 2011; Spoendlin, 1972) that are the perceptual cells of hearing. Multiple innervations of IHCs help to extract different qualities of sound through individual SGN subtypes. Type I neurons have long been known to differ in sensitivity to sound and spontaneous neural activity (Kiang et al., 1965; Merchan‐Perez & Liberman, 1996) that contributes considerably to the prodigious dynamic range of sound perception and temporal resolution. Many factors contribute to neural diversity, such as receptors that influence activity, ion channels, ribbon synapse variability, or efferent tuning of SGN activity (Davis & Crozier, 2015; Petitpre et al., 2018; Reijntjes & Pyott, 2016; Rusznak & Szucs, 2009). Expression patterns and localization of molecular components that shape these physiological characteristics remain elusive, and a meticulous analysis of all candidates is necessary to decipher the coding of sound.

Intrinsic firing properties are mainly generated and formed by ion channels. Among voltage‐gated channels, hyperpolarization‐activated cyclic nucleotide‐gated (HCN) channels generate an inward current (*I*_h_) through the entrance of sodium and potassium ions. Four different subunits (HCN1–4) assemble to form homo‐ or heterotetramers that broaden biophysical characteristics. HCN1 is the subunit with the fastest activation kinetics (*τ* = 25–300 ms, depending on the voltage) and lowest half activation voltage near to the resting membrane potential (RMP) (*V*_1/2_ = −70 mV), while HCN4 is the slowest (*τ* presents a few hundred of ms, *V*_1/2_ = −100 mV). HCN2 and HCN3 demonstrate gating characteristics in between (*V*_1/2_ = −99 mV to *V*_1/2_ = −77 mV, respectively; Wahl‐Schott & Biel, 2009). HCN channels are short‐term regulated by cyclic nucleotides such as 3′,5′‐cyclic (cAMP). When cAMP joins to HCN2 and HCN4, the opening of the channel is accelerated; therefore, the activation time is reduced. However, HCN1 and HCN3 are not modulated by cAMP binding, as noted in the comprehensive review by Sartiani et al. (2017). HCN channels are key regulators of neuronal excitability (Wahl‐Schott & Biel, 2009) controlling the RMP, lowering activation thresholds, and shortening excitatory postsynaptic potentials (EPSPs), maintaining those signals briefly by avoiding summation. These channels act as pacemakers in heart muscle cells and have been hypothesized to generate spontaneous neural firing (He et al., 2014; Sartiani et al., 2017; Shah, 2018). This qualifies them as a potential component to determine neuronal subtypes in the cochlea.

Although several studies have examined physiological functions and the presence of HCN channels in the auditory (Horwitz et al., 2011; Kim & Holt, 2013; Liu, Lee, et al., 2014; Liu, Manis, et al., 2014; Ramakrishnan et al., 2012; Shen et al., 2018; Yi et al., 2010) and vestibular systems (Almanza et al., 2012; Horwitz et al., 2011, 2014), there are some controversies and a lack of information about their subcellular location (Horwitz et al., 2010; Ramakrishnan et al., 2009, 2012).

We performed an immunohistochemical analysis across different mammalian species including CBA/J, C57Bl/6N, Ly5.1 mice, guinea pigs, cats, and human subjects to determine subcellular location and co‐expression of all four HCN subunits. Quantitative changes in key molecules in age‐related hearing loss (ARHL) of C57Bl/6N and CBA/J mice potentially provide a tool to identify functional implications. HCN expression levels and hearing performance were therefore tested to identify any correlation and data were further used to identify common trends that these inbred strains share.

With our results, we aimed to address the following questions:

1. Are there marked differences in HCN expression among mammalian species?
2. Do HCN expression levels correlate with hearing performance during aging in mouse strains with different types of ARHL?
3. How is the spatiotemporal HCN expression pattern during mouse postnatal development associated with known data on auditory function?

The findings of this study shall provide a robust basis upon which to assess possible functions and better estimate HCN involvement in neuronal coding of sound.

## 2 MATERIAL AND METHODS

### 2.1 Animals and human temporal bones

C57Bl/6NCrl (strain 027; Charles River, Germany), JAX™ CBA/J (JAX Nr. 000656; Charles River, France), and Ly5.1, a CD45.1 expressing B6.SJL‐Ptprca Pepco/BoyCrl strain (strain 494; Charles River, Italy), were used to study murine species. The C57Bl/6N strain present an early onset and progression of ARHL and was compared with the CBA/J strain that shows a slower rate of ARHL (Kane et al., 2012). Ly5.1 mice share SGN characteristics of an unmyelinated neuron soma with humans (Jyothi et al., 2010) and served as a “human‐like” animal model of SGNs. The animal studies conformed to the Austrian guidelines for the care and use of laboratory animals and were approved by the Austrian Federal Ministry of Education, Science and Research (reference number BMWFW‐66.011/0120‐WF/V/3b/2016). Animals were bred until different time points from 1 day to 19 months of age. Ears were fixed with 4% formaldehyde (PFA) overnight followed by decalcification in 20% ethylenediaminetetraacetic acid (EDTA). There were no gender differences in age‐related hearing thresholds found in C57Bl/6N strains until 32 kHz (Hunter & Willott, 1987). In CBA/J, no difference in hearing deficits were previously reported until 18 months of age, when females showed faster deterioration of hearing than males (Ohlemiller et al., 2010). Because of these results, we did not account for gender effects. All 65 CBA/J (37 males and 28 females) and 84 C57Bl/6N (30 males and 54 females) were bred in the same room at the Innsbruck animal facility with a 12‐hr dark–light cycle and unlimited access to food and water. Sections from cat and guinea pigs served only for comparative studies of subcellular HCN location. This archival material lacked data about the sex and was used in previous studies (Glueckert et al., 2008; Rattay et al., 2013). Since we did not find any differences in immunolocalization of HCN channels between female and male murine species, we regarded sex information as not relevant. For our experiments, we tested pure‐tone hearing thresholds in different age periods including young animals (1–4 months); 5–8 months when C57Bl/6N started losing hearing (Hunter & Willott, 1987); 9–12 months; 13–15 months; and 15–19 months which covers ARHL in CBA/J (Ohlemiller et al., 2010). We added groups for immunolabeling analysis without functional hearing (P0–P14) and a young hearing animal group with an age that matched the final maturation period (P15–P31).

Human tissue from 10 different temporal bones was collected by the Division of Clinical and Functional Anatomy of the Medical University of Innsbruck. All the individuals were anonymized and had donated their bodies voluntarily to scientific research via their informed consent prior to death (McHanwell et al., 2008; Riederer et al., 2012). There was no evidence of any ear malformation. The time postmortem varied up to a maximum of 13.5 hr before they were fixed with 4% PFA for 48 hr. Sex and ages of individuals were not reported due to anonymization rules. Tissue was decalcified in 20% EDTA at 37°C. Human and animal specimens were cryoembedded as described elsewhere (Coleman et al., 2009) and cut at 10 µm.

### 2.2 Auditory brainstem responses

Evoked auditory brainstem response (eABR) testing was performed with a custom‐made system (Otoconsult, Frankfurt am Main, Germany). A multifunction data acquisition device (USB‐6251; National Instruments, Austin, TX, USA) was used for providing stimuli and recording of evoked potentials with Audiology Lab v.36 software (Marcus Müller, University Tuebingen, Germany). A total of 77 mice (4–10 in each group) were used (28 males and 49 females) for audiometric testing. Animals were anesthetized with ketamine (Ketasol 100 mg/ml; Animedica, Senden, Germany), xylazine (Rompun, 20 mg/ml; Bayer, Leverkusen, Germany), and atropine (Atropinum sulfuricum [Nycomed] 0.5 mg; Takeda, Tokyo, Japan). Measurements were performed in a custom‐made anechoic chamber. Potentials were recorded via three subcutaneous needle electrodes. Tone bursts of 4, 8, 16, and 32 kHz served as stimuli. Tone pips of 3‐ms duration (1‐ms rise and fall time) were presented at a rate of 60/s with alternating phases. Frequency‐specific sound pressure levels were adjusted with a custom‐made attenuator, and tone pips were amplified with a custom‐made amplifier. Stimuli were delivered in free field to a Beyer DT911 loudspeaker calibrated in situ prior to each measurement with a KE4 microphone (Sennheiser, Wedemark, Germany). A software averager included an artifact rejection algorithm to facilitate the extraction of the evoked potentials from unwanted noise such as heart beating and muscle activity. After amplification (100 dB) and band‐pass filtering (high pass: cut off 220 Hz, 0 dB gain; low pass: cut off 5,000 Hz, 80 dB gain), electrical signals were averaged (64 repetitions each phase). Starting with 0 dB, the stimuli were increased in 5 dB steps up to 90 dB. Hearing thresholds were determined as the minimum stimulation level that produced a clearly recognizable potential. Recordings were evaluated by two independent researchers in a blinded manner. According to previous publications, we assigned 4 kHz to an apical‐, 8 kHz to an apex‐middle‐, 16 kHz to a middle‐, and 32 kHz to an upper basal turn (Liu et al., 2019) location.

### 2.3 Immunohistochemistry

Antibody characteristics are summarized in Tables 1 and 2. Within each run, control slides were included by substituting the primary antibodies with isotype‐matched controls, preabsorbing the primaries with immunogenic peptide according to the manufacturer's recommendation, or omitting the primary antibody. In double staining experiments, single antibody stainings and isotype‐matched primary antibodies served as quality controls. Evaluation of positive and negative immunoreactivity was done in various mouse tissues, such as kidney, olfactory epithelium, or brain (Figures S1 and S2). Western blot characterization of each HCN primary antibody are published on the company website (www.alomone.com). All HCN antibodies used here had been extensively used in previous work, including auditory research. The specificity of HCN1, −2, and −4 antibodies were validated with gene knockout tissue.

**TABLE 1:** Primary antibody summary table

Primary antibody	Immunizing antigen	Manufacturer/RRID/Cat/Lot/Host/Clones	Working dilution
CASPR (CNTNAP1)	Fusion protein 1308–1381 (cytoplasmic domain) of rat CASPR	Origene (Maryland, US)/Cat# TA326340/Lot. 1012/S65‐35/mouse/monoclonal	1:100
CG1 Calretinin	Recombinant human calretinin containing a 6‐his tag at the N‐terminal	Swant (Switzerland)/RRDI: AB_10000342/Cat#7697/Lot.1893‐0114/goat/polyclonal	1:1,000
HCN1	Peptide (C)KPNSASNSRDDGNSVYPSK (amino acid residues 6–24 of rat HCN1)	Alomone Labs (Jerusalem, Israel)/RRID:AB_2756625/Cat#AGP‐203/Lot.AGP203AN0202/guinea pig/polyclonal	LM: 1:300 Q: 1:900 F: 1:100
HCN2	Peptide (C)EEAGPAGEPRGSQAS (amino acid residues 147–161 of human HCN2)	Alomone Labs (Jerusalem, Israel)/RRID:AB_2313726/Cat#APC‐030/Lot.APC030AN1650/rabbit/polyclonal	LM: 1:3,000 Q: 1:6,000 F: 1:500
HCN2‐ATTO‐594	Peptide (C)EEAGPAGEPRGSQAS, corresponding to amino acid residues 147–161 of human HCN2.	Alomone Labs (Jerusalem, Israel)/RRID:AB_2341028/Cat#APC‐030‐AR/Lot. APC030ARANO150/rabbit/polyclonal	F: 1:500
HCN3 extracellular	Peptide (C)ELEPRLDAEVYK (amino acid residues 190–201 of rat HCN3)	Alomone Labs (Jerusalem, Israel)RRID:AB_2756742/Cat#APC‐083/Lot.APC057AN0150/rabbit/polyclonal	1:1,000
HCN4	GST fusion protein with the sequence HGHLHDSAEERRLIAEGDASPG EDRTPPGLAAEPERP (amino acid residues 119–155 of human HCN4)	Alomone Labs (Jerusalem, Israel)/RRID:AB_2039906/Cat#APC‐052/Lot.APC052AN2025/rabbit/polyclonal	LM: 1:1,000 Q: 1:7,000 F: 1:200
Peripherin	Electrophoretically pure trp‐E‐peripherin fusion protein [Dev. Brain Res. (1990) 57:235–248], containing all but the 4 N terminal amino acids of rat peripherin	Millipore (Darmstadt, Germany)/RRID:AB_90725/Cat#AB1530/Lot. 2488786/rabbit/polyclonal	1:2,500
Prestin	Raised against a peptide mapping at the N‐terminus of prestin of human origin	Santa Cruz Biotechnology (Texas, US)/RRID:AB_2302038/Cat# sc‐22692/goat/polyclonal	1:250

Note

Cat., catalogue number; Lot, lot number. Primary antibody dilutions were optimized for colorimetric visualization (Light microscopy‐LM), quantification (Q) as well as fluorescent detection (F).

**TABLE 2:** Secondary antibody summary table

Secondary antibody	Target/Host/Specificity	Manufacturer/RRID/Cat/Lot/Clones	Working dilution
biotin‐SP *F*(ab′)₂ anti‐guinea pig	Guinea pig/donkey/IgG	Jackson ImmunoResearch Labs (Pennsylvania,USA)/RRID:AB_2340452/Cat# 706‐066‐148/Lot. 117526/polyclonal	1:2,000
biotin‐SP‐conjugated anti‐rabbit	Rabbit/donkey/IgG	Jackson ImmunoResearch Labs (Pennsylvania, USA)/RRID:AB_2340593/Cat# 711‐065‐152/Lot.136387, Lot.133152, Lot.140405/polyclonal	1:400
anti‐goat E0466	Goat/rabbit/IgG	Agilent Dako (CA, USA)/RRID:AB_2617143/Cat# E046601‐2/Lot. 20028689/polyclonal	1:400
Alexa Fluor® 647 AffiniPure *F*(ab′)₂	Guinea pig/donkey/IgG	Jackson ImmunoResearch Labs (Pennsylvania, USA)/RRID:AB_2340477/Cat# 706‐606‐148/Lot. 140821, Lot.131664/polyclonal	1:2,000
Alexa Fluor® 488 AffiniPure	Rabbit/donkey/IgG	Jackson ImmunoResearch Labs (Pennsylvania, US)/RRID:AB_2313584/Cat# 711‐545‐152/Lot. 1674651/polyclonal	1:2,000
DyLight® 649	Rabbit/goat/IgG	Vector Laboratories (CA, USA)/RRID:AB_2336420/Cat# DI‐1649/Lot. Z0820/polyclonal	1:500
DyLight® 649	Mouse/horse/IgG	Vector Laboratories (CA, USA)/RRID:AB_2336419/Cat# DI‐2649/Lot. ZA0424/polyclonal	1:600

Note

H + L antibodies recognize heavy and light chain of antibody molecules, while *F*(ab′)₂ antibody fractions have no Fc portion.

#### 2.3.1 Automated immunohistochemistry

Most immunostainings were performed on a fully automated system (Ventana Discovery; Roche Medical Systems Inc., Mannheim, Germany, RRID:SCR_013652) and visualized with its DAB‐MAP (760‐124), Red‐MAP (760‐123), Blue‐MAP (760‐120), and Ultra‐MAP (760‐4314) detection systems or with fluorescent‐labeled antibodies (Table 2). Double immunostainings with colorimetric visualization were performed consecutively with a heat‐induced denaturation step in between (85°C, 8 min) a 3,3′‐diaminobenzidine (DAB) and 3‐amino‐9‐ethylcarbazole (AEC) staining. Heat‐induced antigen retrieval was done with a tris‐EDTA (950‐124) or citrate‐based buffer (950‐123). For semi‐quantitative evaluation, only DAB‐processed sections were used and counterstained with hematoxylin (DAKO, Carpenteria, CA, USA).

#### 2.3.2 Manual immunohistochemistry

After blocking (1 hr, 30% normal donkey serum and 0.3% Triton X‐100), primary antibodies were incubated overnight at 4°C, and then for 1 hr at RT, followed by secondary antibody exposure for 2 hr at RT. Mounting in Vectashield‐DAPI (Cat# H‐1200, RRID:AB_2336790; Vector Laboratories Inc, Burlingame, CA, USA) provided nuclear staining. In experiments with primary antibodies raised in the same species (rabbit anti‐HCN2 and rabbit anti‐HCN4), a heat denaturation step (8 min at 81°C) between immunostainings was applied.

### 2.4 Quantification of neuronal staining and imaging

The membrane staining around the SGN somata was acquired with a PixeLink PL‐B623 camera (Pixelink®, Canada) at 2,048 x 1,536 pixel resolution on a TisseFaxPlus® microscope system equipped with 40x/1,3 lens, and quantification was performed with the dedicated image analysis software Histoquest® 6, (RRID:SCR_014823; both from Tissuegnostics®, Vienna, Austria). A software internal color deconvolution strategy separated hematoxylin from DAB staining. An algorithm was trained to segment specifically SGNs by their hematoxylin nuclei staining considering size, shape, mean staining intensity, and color shade until SGN recognition was appropriate. A growing mask around these nuclei served to label a ring‐shaped area around the cell membrane in a reproducible way. Mean intensity of gray values for the DAB immunoreactivity was calculated for each individual neuron. We stained sections from 37 different mice in CBA/J (20 males and 17 females) and 36 C57Bl/6N (19 males and 17 females). The minimum criteria of sections from three different mice for each cochlear turn were met for all age groups. In total, 31,580 individual neurons were evaluated: HCN1 (10,738 SGNs), HCN2 (9,901 SGNs), and HCN4 (11,030 SGNs). Fluorescent double stainings were imaged with confocal microscopes (LSM 510 Meta and LSM980 with Airyscan2; Carl Zeiss, Göttingen, Germany). For co‐expression studies, identical acquisition parameters were applied on 438 SGNs and single channel images evaluated with Image J. Colorimetric double stainings were color deconvolved with Image J (www.imagej.nih.gov, version 1.52n, algorithm FastRed‐FastBlue‐DAB) for visualization purposes only. Super‐resolution confocal imaging for HCN double staining experiments on 33‐day‐old CBA/J mice resulted in a lateral resolution of approximately 220–300 nm. Electron microscopy data arose from previous work. Protocols were published for transmission electron microscopy (TEM; Glueckert et al., 2008) and scanning electron microscopy (SEM; Glueckert et al., 2005).

### 2.5 Experimental design and statistical analysis

The number of animals based on sample size calculation studies is extensively described elsewhere (Arifin & Zahiruddin, 2017; Charan & Kantharia, 2013). Initial sample size estimation premised normal distributed data. Due to the huge variability of HCN expression in individual SGNs increasing with age, we had to increase the number of mice for the immunohistochemical semi‐quantification experiments over the number calculated using the resource equation approach. Males and females were used indistinctly. Statistical tests were performed with IBM SPSS statistics v.24 (RRID:SCR_002865; IBM, Armonk, NY, USA). Normal distribution of data was tested with the Kolmogorow–Smirnow and Shapiro–Wilk test. Data obtained on semi‐quantification of staining and most of the ABR measurements were not normally distributed; hence, non‐parametric tests were applied. The Kruskal–Wallis test, followed by Dunn's post hoc test and Bonferroni correction, was used to test statistical significances (*p* < 0.05 was considered significantly different). The co‐expression relationship was checked with nonparametric Kendall rank test, where the correlation coefficient tau‐b (*τb*) indicated the strength of the co‐expression.

## 3 RESULTS

### 3.1 HCN localization

#### 3.1.1 HCN1 localization

**FIGURE 1:**
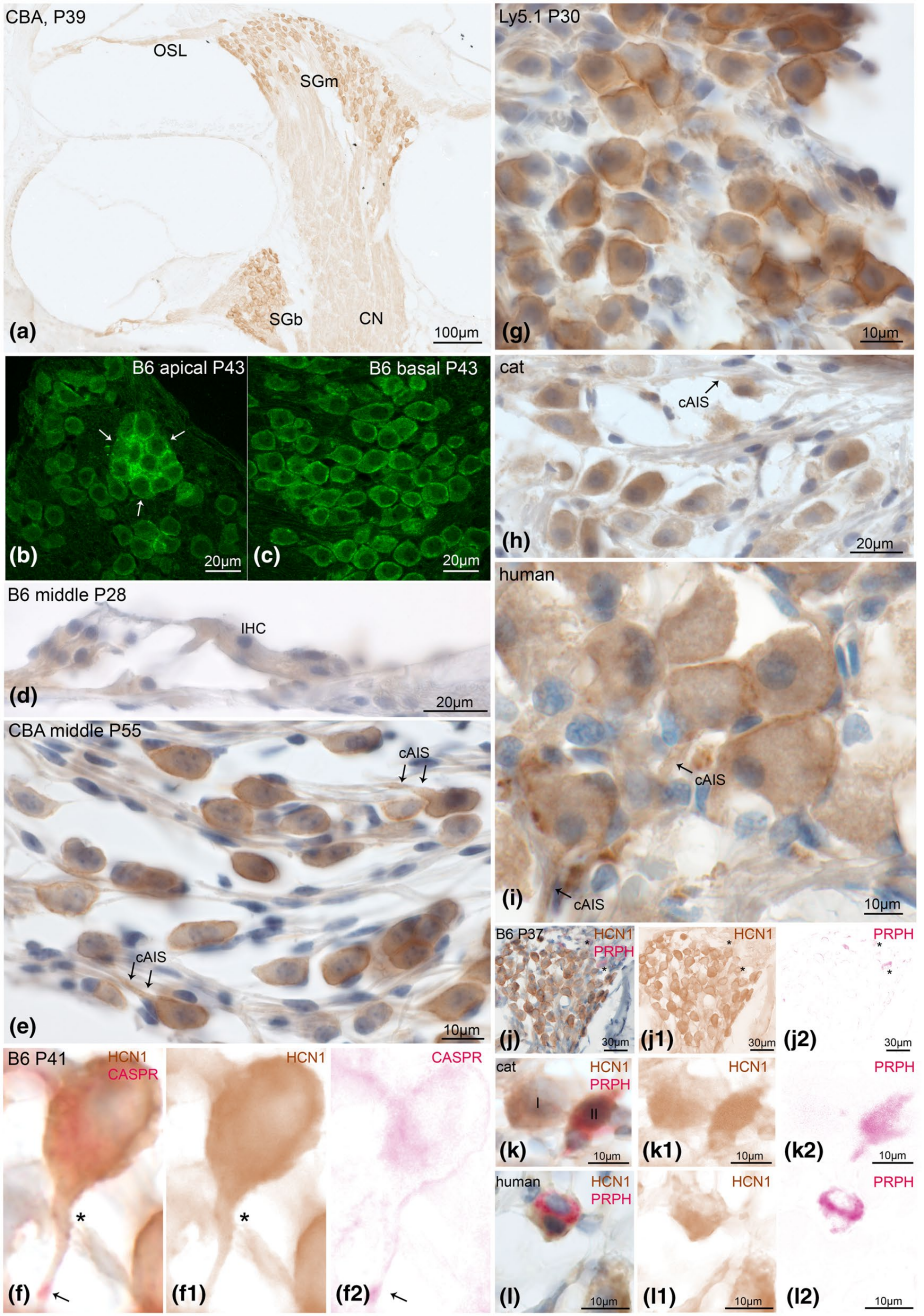
HCN1 expression in the adult mammalian cochlea. HCN1 channel subunits were highly expressed in neurons with intense cytoplasmatic and perisomatic membrane staining. a, CBA/J spiral ganglion neurons (SGNs) somatic staining with DAB. There was no visible gradient across SGNs of the middle (SGm) and basal (SGb) turns. The central cochlear nerve (CN) and axons in the osseous spiral lamina (OSL) were weakly stained. Confocal imaging in C57Bl/6N showed highest intensity of HCN1 at neuronal clusters (arrows) in the apical turn (b); neuron soma membrane staining in the basal turn (c). d, No specific staining was found in the sensory epithelia; inner hair cell (IHC). e, Intense staining at the axon hillock and central axon initial segment (cAIS, arrows). No staining was detected at the peripheral AIS. f, Double staining of HCN1 (f1) with contactin‐associated protein 1 (CASPR, f2, arrow) identified HCN1 staining to overlap with the cAIS. g, Neurons in Ly5.1 presented staining with higher intensity. Cat (h) and human (i) shared equal HCN1 distribution at the neuron soma and cAIS (arrows). j, Double staining with peripherin (PRPH), a specific type II SGN marker does not co‐express with HCN1 in C57Bl/6N (asterisk), but in cat (k) and human (l). A type II SGNs cluster in human presented different intensities of HCN1 (l1). f1, j1, k1, l1, DAB channel (HCN1), f2, j2, k2, l2 AEC channel (CASPR1 and PRPH) after color deconvolution. B6, C57Bl/6N; CBA, CBA/J; P, postnatal day

HCN1 was located at the plasma membrane of type I SGNs in mice (Figure 1a–g, cat (Figure 1h), and human (Figure 1i). Weaker and more diffuse HCN1‐like immunoreactivity (HCN1‐LI) in the cytoplasm of neural somata possibly indicated protein synthesis (Figure 1a–l). No cell types other than SGNs showed any immunoreactivity (Figure 1d). Confocal images presented elevated staining intensity in neuronal clusters that were frequently found in cochlear turns toward the apex in C57Bl/6N (Figure 1b) and Ly5.1 mice (Figure 1g). The majority of other neurons showed a highly variable degree of HCN1‐LI intensities across cochlear turns ranging from untinged to intensely stained (Figure 1a–q). Ly5.1 presented the most intense staining among murine species (Figure 1g). Postsomatic segments comprise the axon initial segment (AIS) that generates and shapes the action potentials (APs) and maintains neuron polarity (Huang & Rasband, 2018). Some authors' distinct a peripheral axon initial segment (pAIS) and a central axon initial segment (cAIS) located at the pre‐ and postsomatic segments, respectively (Hossain et al., 2005). Pre‐ and postsomatic segments can be identified by anatomical position and the fact that the central axon is double the diameter of the peripheral axon (Potrusil et al., 2012). We could frequently observe HCN1‐LI in the postsomatic region, but never observed staining at presomatic sites (Figure 1e). Double stainings with contactin‐associated protein 1 (CASPR 1) showed HCN1 staining along the entire cAIS (Figure 1f). Previous studies in motor neurons localized CASPR 1 at the para‐AIS compartment. This site marks the distal end of AIS Nav channel distribution and start of the axonal myelination in CNS neurons (Duflocq et al., 2011). Both of these segments and the soma are fully myelinated in rodents and cats, while most type I SGNs in humans completely lack myelination at these neuronal segments. The length of postsomatic segments varies considerably in humans from more than 30 µm to not identifiable (Liu et al., 2015).

Tonotopical gradients of HCN1 expression were not static but changed among different mouse strains and within different age groups (semi‐quantified in Figures 10 and 11). Adult CBA/J mice showed a clear apical‐basal gradient with more HCN1 toward the apex (Figures 1a, 10a, and 11a) while there was no clear gradient in adult C57Bl/6N (Figures 1b,c, 10d, and 11b).

Double staining experiments with peripherin as a selective marker for adult type II SGNs revealed a lack of HCN1‐LI in this cell type in mice (Figure 1j). In human and cat samples, few type II cells were stained (Figure 1k–l).

Summing up, HCN1 sits in the cell body membrane and postsomatic segment of type I neurons, suggesting that the action of HCN1 is limited to the SGN soma. SGN clusters showed the highest immunostaining. Type II SGN somata were positive in humans and cats.

#### 3.1.2 HCN2 localization

**FIGURE 2:**
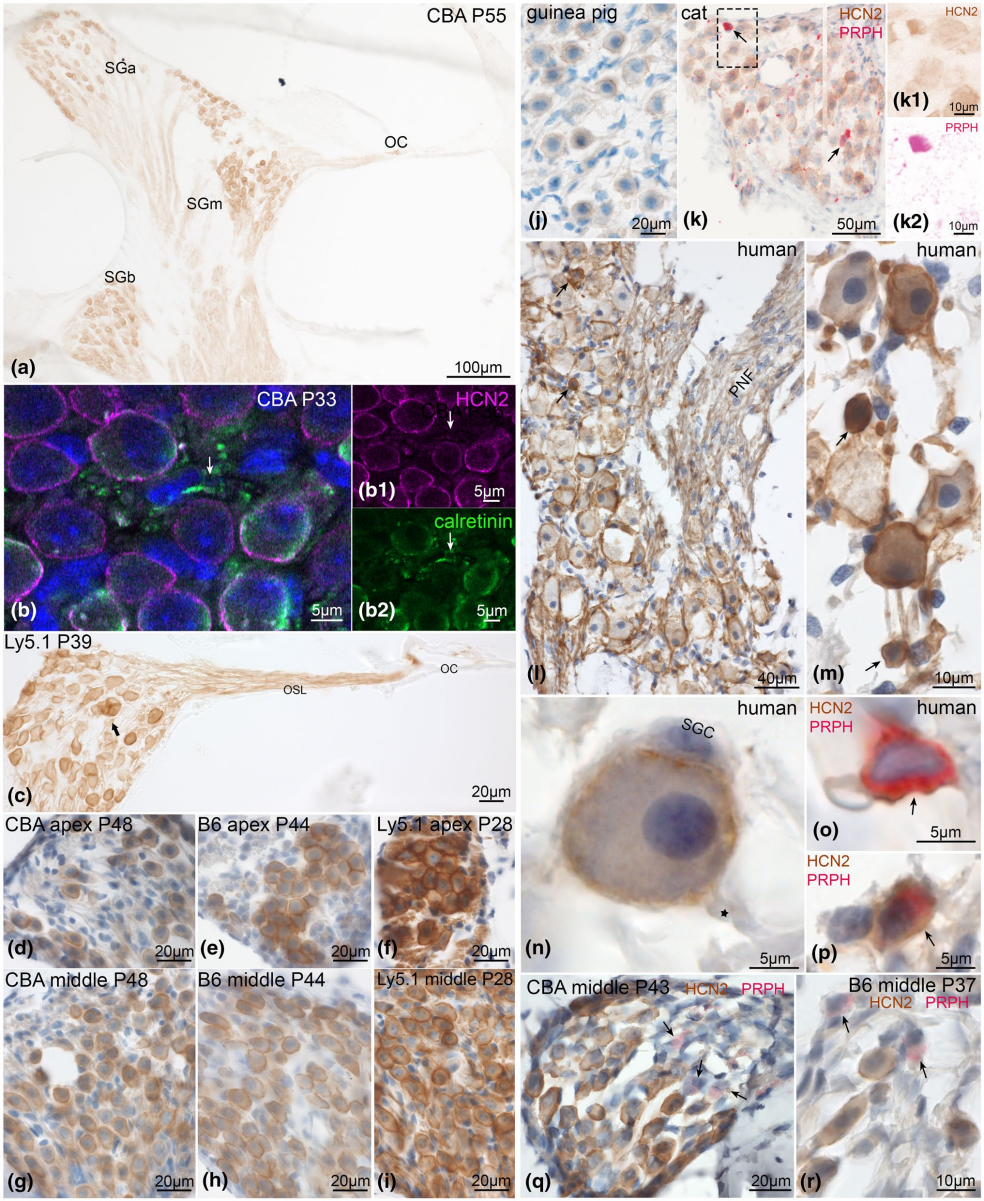
HCN2 expression in the adult mammalian SGNs. HCN2 presented staining at the neuronal soma membrane and axons. a, Midmodiolar view exposed immunostaining in the organ of Corti (OC) and a rather homogeneous staining along the tonotopical axis in CBA/J across the spiral ganglion in the apical (SGa), middle (SGm), and basal (SGb) turn. b, Super‐resolution confocal imaging with calretinin as neuronal counterstaining and DAPI nuclear stain. b1, direct‐conjugated HCN2 antibody signal, b2, calretinin immunoreactivity. HCN2 was present only in perisomatic SGN membranes, axons (arrows) are void of any HCN2‐LI. c, High intensity of the staining was visible in a Ly5.1 mouse in the apical turn within a neuron cluster (arrow), along the osseous spiral lamina (OSL) and in the organ of Corti (OC). d–i, Comparison of neuronal staining in three inbreed mouse strains identified similar expression levels of HCN2 in CBA/J (d, g) and C57Bl/6N (e, h) but higher intensity of staining in Ly5.1 mice (f, i). HCN2 in type I neurons of a guinea pig (j), type I and peripherin (PRPH)‐positive type II neurons in cat (k) and human (l–p); k1 DAB channel (HCN2), k2, AEC channel (PRPH), after color deconvolution. l, Peripheral nerve fibers (PNF) appear weakly positive in human. m, Small human type II neurons were heavily stained for HCN2 (arrows). n, High magnification of human type I SGNs showed intense staining at the soma membrane, and weaker in the cytoplasm; postsomatic segment (asterisks) and the satellite glia cell (SGC) were void of reactivity. Double staining with PRPH was positive for HCN2 in human type II neurons (arrows) (o, p), but negative in CBA/J mice (q) and C57Bl/6N (r). B6, C57Bl/6N; CBA, CBA/J; P, postnatal day

Most intense HCN2 immunostaining occurred in the membrane around the soma of type I SGNs in mouse (Figure 2a–i,s–t), guinea pig (Figure 2j), cat (Figure 2k), and human specimens (Figure 2l–p). We could clearly locate HCN2 to perisomatic and not to axonal membranes with super‐resolution confocal imaging (Figure 2b). Heterogeneity of individual neuron staining was similar to HCN1, as well as having high intensity in neuron clusters (Figure 2c) and enhanced immunoreactivity in Ly5.1 mice (Figure 2c,f,i). HCN2 appeared more intensely stained in the apex of CBA/J (Figure 2a,d,g), C57Bl/6N (Figure 2e,h), and Ly5.1 mice (Figure 2f,i). Type II afferents revealed staining in cats (Figure 2k) and humans (Figure 2m,o,p) but were void of immunoreactivity in CBA/J and C57Bl/6N (Figure 2q,r).

**FIGURE 3:**
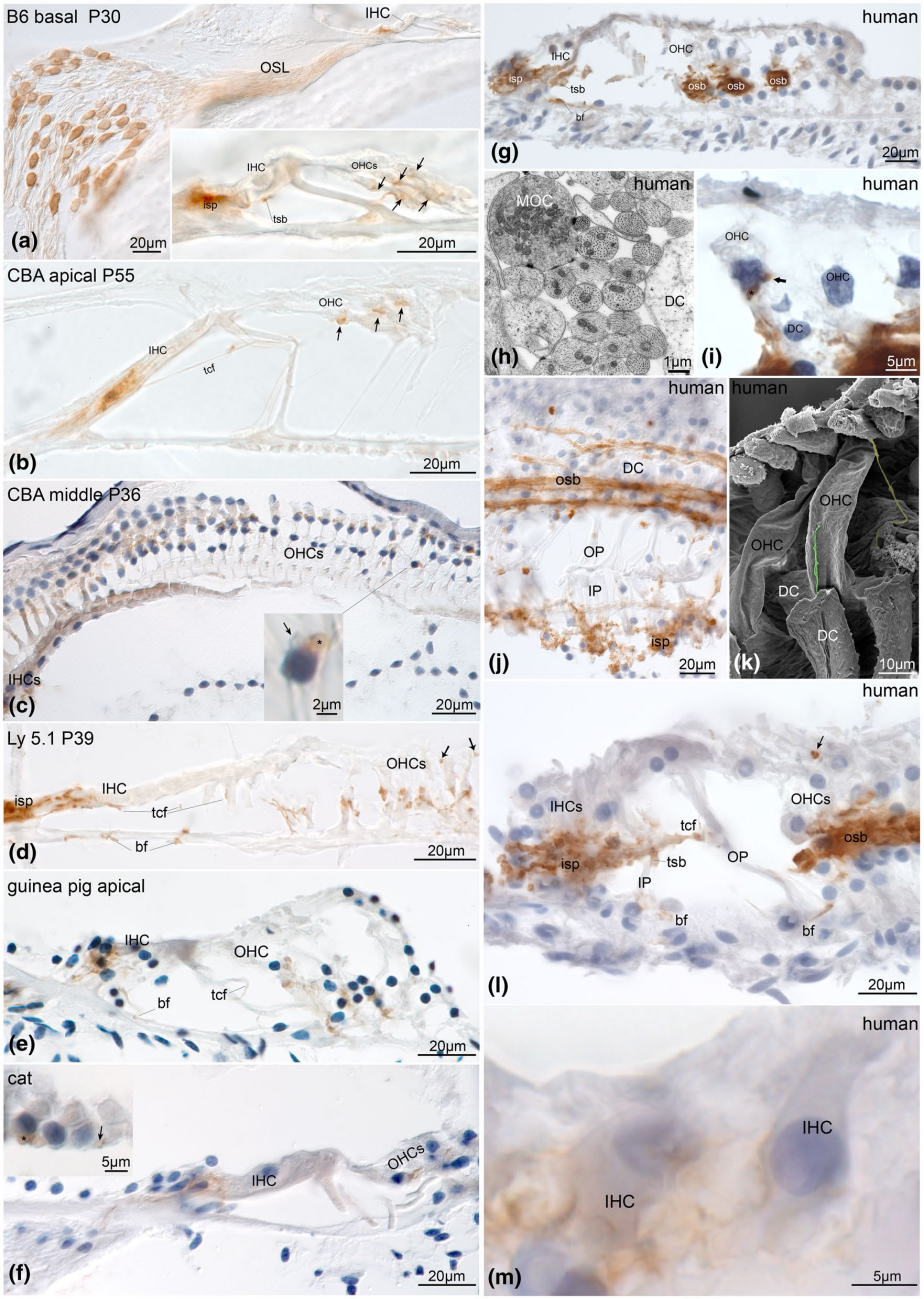
HCN2 expression in the adult mammalian sensory epithelia. HCN2 was present at afferent and efferent nerve fibers innervating the organ of Corti. a, Differential interference contrast image (DIC) of DAB visualized immunostaining showed presence in C57Bl/6N neurons, osseous spiral lamina (OSL), and underneath the inner hair cell (IHC). The inset with a magnified view of the organ of Corti exposed staining at the inner spiral plexus (isp) underneath the IHC, boutons and nerve fibers between outer hair cells (OHCs) and supporting cells (arrows) and the efferent fibers of the tunnel spiral bundle (tsb). b, Similar staining in CBA/J in the organ of Corti with expression underneath IHC and OHCs (arrows). Positive bigger caliber tunnel crossing fibers (tcf) account for the medial efferent innervation. c, DIC of a CBA/J horizontal orientation, inset: high magnification clearly showed staining in the large efferent synapses (asterisk), while smaller putative afferent type II terminals (arrow) were void of immunoreactivity. d, Ly5.1 mice HCN2 staining underneath the IHC, fibers traveling at the base of the tunnel (basilar fibers, bf) and thin caliber tcf (arrows) represent type II afferent fibers. Large boutons underneath OHCs correspond to the efferent innervation. Guinea pig (e) and cat (f) organ of Corti showed the same HCN2 staining pattern like Ly5.1 mice with type I and type II afferent and efferent fibers stained. In cat, the inset shows staining in the large synapses underneath OHCs from the medial efferent fibers (asterisk) and smaller putative type II terminals (arrow). Human sensory epithelia presented high levels of HCN2 expression at equal localization as in animals (g‐m) emphasized in the massive staining in outer spiral bundles (osb) typical for human. g, Immunopositive isp and bf (type II afferents), as well as medial efferent fibers of the tsb. The prominent osb comprises type II afferent as well as efferent fibers shown in (h) in transmission electron microscopy. The figure expose the different nerve fibers of the osb that are difficult to distinguish at light microscopic level. Big caliber efferent (medial olivocochlear fibers, MOC) intermingle with small‐sized type II afferents that are numerous in human. i, Bigger efferent nerve terminals (asterisk) adhere around the basal pole of OHCs together with smaller putative type II afferent terminals (arrow, DC, Deiters cell). j, Horizontal orientated sections from the human organ of Corti confirmed HCN2 staining in isp, osb and fibers crossing Corti's tunnel, (OP, outer pillar; IP, inner pillar). k, Scanning electron microscopy of human OHCs depicts thin caliber nerve fibers (colored nerve fibers) that climb up OHCs as far as the reticular lamina (yellow‐colored fiber). These fibers are positively labeled for HCN2 in human (l, arrow) and mice (d) marked with arrows. The function of these fibers is unknown. l, Another human specimen with positive HCN2 staining m, High magnification of human IHCs identified type I afferents nerve terminals positive for HCN2. B6, C57Bl/6N; CBA, CBA/J; P, postnatal day

HCN2 was not restricted to the spiral canal but showed its presence in the organ of Corti. The spiral plexus was intensively stained and comprised efferent as well as afferent nerve fibers that innervate hair cells (Figure 3). Axonal transport to these sites may also explain the diffuse immunostaining in the osseous spiral lamina (OSL) (Figures 2c and 3a). Immunostaining embraced the basal aspect of IHCs in all species (Figure 3a–m). While we were not able to distinguish afferents from efferents in the spiral plexus with conventional imaging techniques, we could track nerve fibers (Figure 3a–d,g,l) that traveled toward the outer hair cells (OHCs). Staining of tunnel spiral bundle (TSB) fibers (Figure 3a,g,l) and large caliber tunnel crossing fibers (TCFs) (Figure 3d,g,l) as well as large synapses at the basal aspects of OHCs (Figure 3a–I) suggested the presence of HCN2 in the medial efferent olivocochlear (MOC) system. Thin caliber TCFs (Figure 3b,j,k) and basilar fibers that travel at the floor of the tunnel of Corti refer to type II afferents. These fibers also showed positive HCN2‐LI in Ly5.1 mice and other mammalian species investigated here (Figure 3d,e,g,j,l) but were lacking in CBA/J and C57Bl/6N mice (Figure 3a,b). The same mouse strains were also void of HCN2 immunoreactivity in type II neuron somata (Figure 2q,r). Some nerve fibers and nerve endings that travel along the lateral wall of OHCs and even contact the very apical reticular lamina (Figure 3d,k,l) were positively stained. The function of these putative efferent nerve fibers is unknown. They were found in human specimens as well (Figure 3k,l). High magnification of human IHCs illustrated HCN2‐LI at the afferent type I synaptic membrane contacting the IHCs (Figure 3m).

In summary, we localized HCN2 in most, but not all type I neurons with varying levels of expression, extending as far as the afferent synapses of the IHC. Type II afferents in most mammalian species showed expression for HCN2 but were lacking in CBA/J and C57Bl/6N mice. Expression in these strains may have dropped below detection levels with classical methods. MOC efferents, including the large OHC efferent synapse, presented positive immunoreactivity while radial efferents contacting type I afferent nerve endings required higher resolution imaging techniques for a reliable identification.

#### 3.1.3 HCN3 localization

**FIGURE 4:**
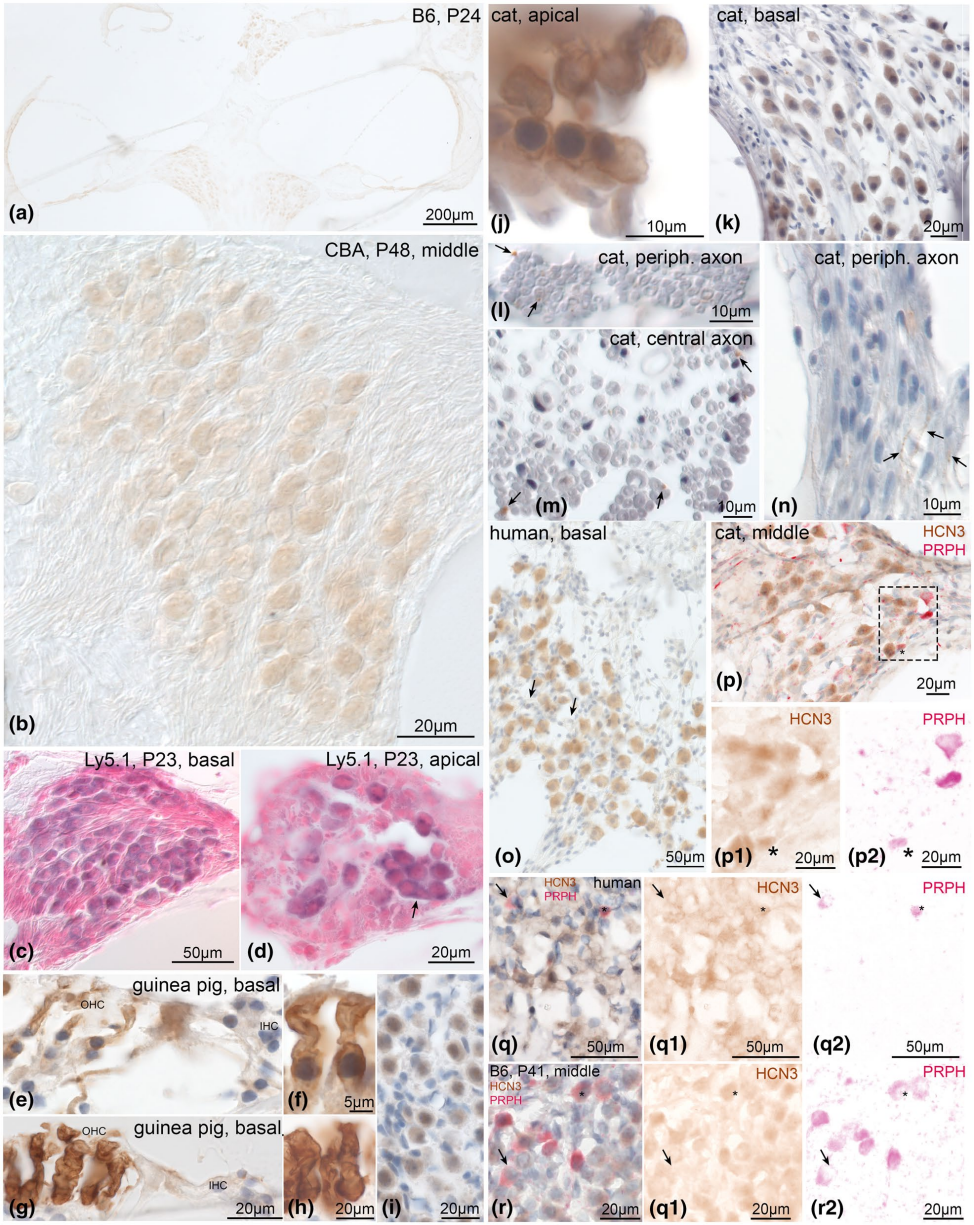
HCN3 expression in the adult mammalian cochlea. HCN3 was weakly expressed in spiral ganglion neuron (SGN) cytoplasm of mice (a–d), guinea pig (i), cat (k), and human (o). In guinea pig and cat HCN3 was detected at the level of outer hair cells (OHCs) (e–f and j). a, In a mid‐modiolar view of C57Bl/6N SGNs presented weak staining. A tonotopic gradient was observed from apex to base. b, CBA/J showed weak staining in neuron cytoplasm. c–d, A more sensitive immunostaining protocol with alkaline phosphatase detection as marker enzyme was performed in Ly5.1; reactivity in apical neuron clusters (d, arrow) was more intense than in basal neurons (c). OHCs in the guinea pig showed specific staining at the lateral membrane (e–f). Staining pattern was similar to the motor protein prestin (g–h). i, HCN3 was also present in the guinea pig SGNs, in cat OHCs (j) as well as cat SGNs (k). l–n, Thin unmyelinated fibers at the peripheral (l, n) and central axon (m) were positive in cat. o, human tissue revealed similar staining as in animal tissue. Most, but not all neurons (arrows) appeared stained. Double staining with peripherin (PRPH) confirmed the presence of HCN3 in type II SGNs (arrows and asterisk) in cat (p), human apical turn SGNs (q) and in C57Bl/6N (r) with varying intensities. p1, q1, r1, DAB channel (HCN3), p2, q2, r2, AEC channel (PRPH) after color deconvolution. B6, C57Bl/6N; CBA, CBA/J; P, postnatal day

HCN3‐LI presented as a very weak staining within neuron cytoplasm (Figure 4a,b) that distinguished it from control slides (Figure S1g–l). Higher sensitivity detection using alkaline phosphatase revealed its presence more clearly in the Ly5.1 strain (Figure 4c,d). Apical neurons seemed to be more intensely stained than basal (not quantified, Figure 4a,c,d). Neuron clusters presented high reactivity (Figure 4d).

Interestingly, guinea pigs and cats produced intense immunoreactivity at the lateral membrane of OHCs (Figure 4e,f,j). Basal aspects where synaptic contacts occupy the membranes were void of any staining (Figure 4e,f). Staining patterns coincided with immunolocalization of prestin (Figure 4g,h), the motor protein of OHCs. There was no clear OHC membrane staining in mice or humans.

We observed a more intense staining in cochlear neurons of guinea pigs (Figure 4i) and cats (Figure 4k). Cross sections of the OSL and central cochlear nerve depicted HCN3‐LI in fibers that were smaller than 1 µm in diameter (Figure 4l,m,n). Longitudinal sections confirmed the presence of very thin positively stained unmyelinated nerve fibers in the OSL between the thicker unstained myelinated axons (Figure 4n). This expression pattern suggested the presence of HCN3 in unmyelinated efferent or type II afferents. Also, human cochlear sections exhibited positively stained type I SGNs (Figure 4o). Peripherin‐positive type II neurons revealed different nuances of rather weakly stained SGNs in cat (Figure 4p), human (Figure 4q), and mouse (Figure 4r).

HCN3 was the least abundant subunit present in type I as well as in type II neurons. We were not able to find it in the membrane of adult afferent cochlear neurons but concentrated in some unmyelinated fibers projecting peripheral as well as central from SGN somata. HCN3 expression in the lateral membrane of OHCs in cats and guinea pigs was an unexpected novel finding.

#### 3.1.4 HCN4 localization

**FIGURE 5:**
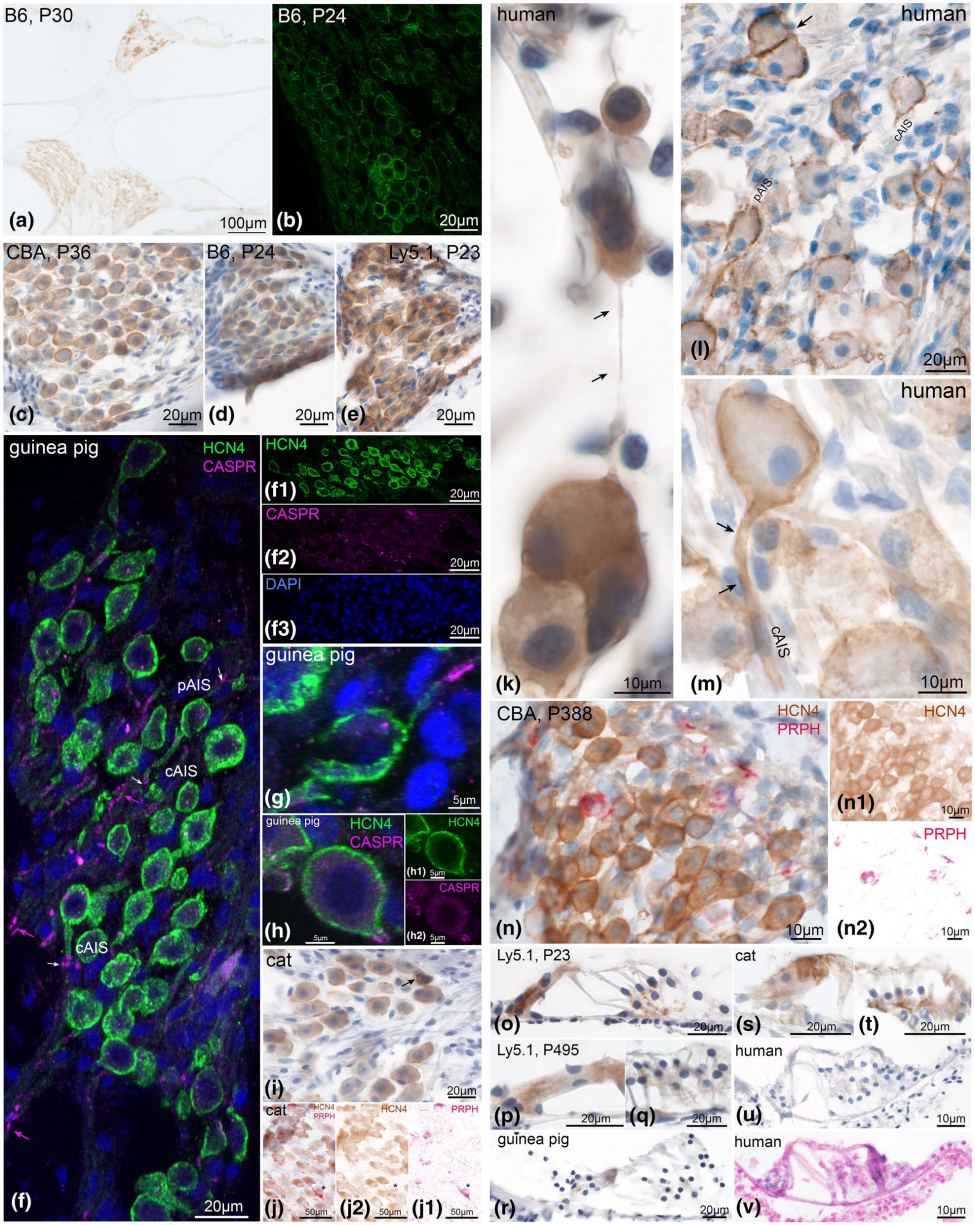
HCN4 expression in the adult mammalian cochlea. a, Mid‐modiolar view of a section from a C57Bl/6N mouse presented visually a tonotopical apex to base gradient. Neurons of the apical turn, especially in neuron clusters were intensely stained. b, Confocal microscopy confirmed the high expression of HCN4 at the neuronal membrane, especially between neurons of a cluster. c–e, HCN4 presented higher intensity of staining in Ly5.1 (e), compared to CBA/J (c) and C57Bl/6N (d). f–h, Confocal microscopy showed HCN4 to be present at the neuronal membrane of type I neurons as well as the peripheral axon initial segment (pAIS) and central axon initial segment (cAIS) in guinea pig (f). CASPR 1 was used as a marker for the distal end of the AISs. AISs lengths differed considerably (f, h). f1, h1, HCN4 staining, f2, h2, CASPR 1 staining, f3, nuclear staining (DAPI). Cat type II neurons presented immunoreactivity in single (i, arrow) and double‐stained sections with additional peripherin (PRPH) staining (j). j1, DAB channel (HCN4), j2, AEC channel (PRPH). Human sections (k–m) presented staining at the perisomatic membrane as well as the cAIS and pAIS. Type II neurons with their delicate unmyelinated axons in humans are positively stained for HCN4 (k). l–m, cAIS length in human varied from very short (l) up to more than 30 µm here (m, arrows). SGN membranes were immunopositive especially in the neuron clusters (arrow, l). n, Double staining with PRPH in a CBA/J mouse showed no co‐expression with HCN4 above background level, n1, DAB channel (HCN4), n2, AEC channel (PRPH) after color deconvolution. o–v, HCN4 expression in the sensory epithelia differed among species: in adult mice, no staining is visible in CBA/J and C57Bl/6N while in Ly5.1 showed staining in young (o) as well as old individuals (p, q). Guinea pig sections presented HCN4 mainly at type I afferents (r), while for cat mainly type II fibers (s, t) were positive. Standard immunohistochemistry methods were not sensitive enough to detect HCN4 in the human organ of Corti (u), but highly sensitive visualization methods using alkaline phosphatase enzymes exposed weak staining underneath the IHC (v). B6, C57Bl/6N; CBA, CBA/J; P, postnatal day

HCN4 immunoreactivity resided predominantly in SGNs' perisomatic membranes (Figure 5). Among type I SGNs, staining intensities of individual neurons were distributed more evenly compared with HCN1 and HCN2. HCN4 was enriched in neuron clusters and exhibited its most prominent staining at membranes facing adjacent neurons within a neuron cluster (Figure 5a–b). There was a tonotopical gradient visible in young C57Bl/6N (Figures 5a, 10f, and 11b) but semi‐quantification of the staining exposed that these grading was not constant (Figures 10f and 11b) throughout lifetime. Similar to HCN1 and HCN2, staining intensity appeared most intense in Ly5.1 mice (Figure 5c–e). Membrane staining in type I SGNs extended to both the pre‐ as well as the postsomatic segments (Figure 5f–h). Confocal image stack projections of double stainings with CASPR 1 to specifically detect the AIS most distal spread were performed (Figure 5f). High magnification of these neurons showed long (Figure 5g) and short (Figure 5h) cAIS as well as pAIS stained for HCN4. This was observed in animal as well as in human sections (Figure 5k–m).

Type II SGNs exhibited positive HCN4‐LI in cat (Figure 5i,j) and human (Figure 5k) and appeared negative in CBA/J and C57Bl/6N mice (Figure 5n). Interestingly, these mouse strains lacked HCN4 staining in the sensory epithelium.

HCN4‐LI was visible in the organ of Corti of Ly5.1 mice and presented at the inner spiral plexus (ISP) and the basolateral aspects of IHCs (Figure 5r). Type II afferent nerve tracks (basilar fibers, outer spiral bundles (OSB), puncta underneath the very basal aspect of OHCs) were immunostained (Figure 5o) while the other mouse strains largely lost staining in the sensory epithelium until the onset of hearing (Figure 5a). Ly5.1 mice preserved immunoreactivity even in 1.5‐year‐old individuals (Figure 5p‐). In adult guinea pigs we detected some staining at the ISP (Figure 5r) while in cats staining was present mainly underneath the OHCs and OSBs (Figure 5s,t). Humans sections appeared negative with standard IHC techniques (Figure 5u), while slight immunoreactivity underneath the IHC was observed with highly sensitive immunohistochemical methods (Figure 5v; Ultra‐MAP).

In summary, HCN4 located quite evenly across the perisomatic SGN membrane and was observed at both the pre‐ and postsomatic segments in all species. Membrane staining did not exceed most distal ends of the cAIS and pAIS. Neural clusters expressed considerably more HCN4. Type II neurons appeared negative in C57Bl/6N and CBA/J but showed reactivity in other mammals at varying intensities. The expression of HCN4 was highly variable at the peripheral nerve endings of type I and type II neurons among investigated mammalian species. Expression in the sensory epithelium was evident in Ly5.1 mice, guinea pig and cat but fell below detection levels with standard immunolabeling techniques in human.

### 3.2 HCN subunit co‐expression and correlative distribution in SGNs of mice and man

**FIGURE 6:**
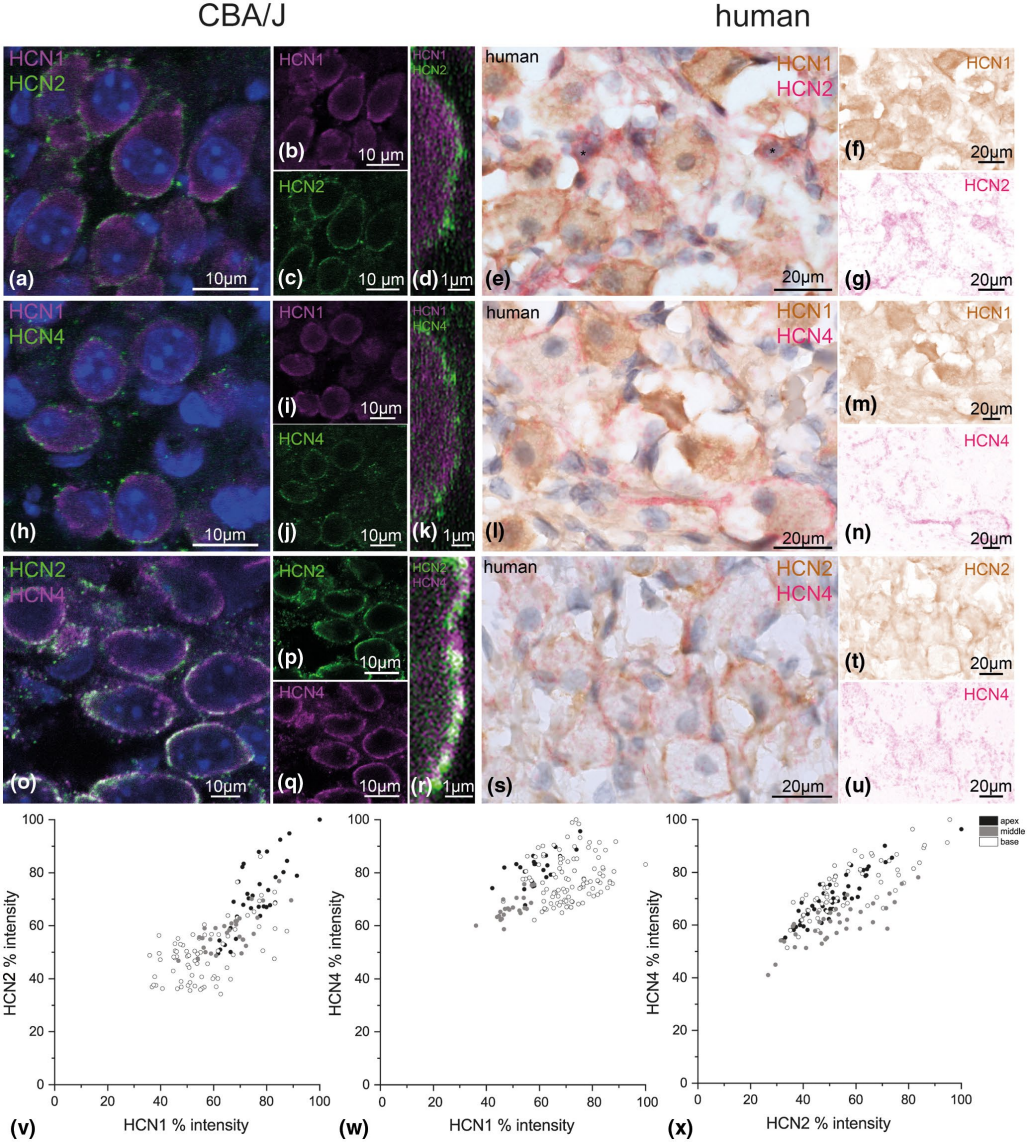
HCN channel co‐expression in SGNs. Co‐expression of HCN1, HCN2, and HCN4 shown in spiral ganglion neurons (SGNs) of 33‐day‐old CBA/J mice and in human. Confocal microscopy was used to unravel the distribution of these channels at the neuronal membrane in basal–middle turn (a–r). Colorimetric immunohistochemistry showed co‐expression in human SGNs in the apical–middle turn (e–u). a–d, HCN1 and HCN2 co‐expression in CBA/J. a, Co‐expression of HCN1 and HCN2 channels at the neuronal membrane. b, c, Single channel expression for HCN1 (b) and HCN2 (c). d, High magnification of the neuronal membrane showed a spotted distribution without color overlapping suggesting homomeric channels. e–g, Co‐expression of HCN1 and HCN2 in human showed a more balanced expression of these subunits, asterisks mark putative type II cells. HCN1 (DAB, f) and HCN2 (AEC, g) channels after color deconvolution. h–k, HCN1 and HCN4 co‐expression revealed a patchy distribution of staining without color overlaps (h). i–j, Single channel staining for HCN1 (i) and HCN4 (j). k, High magnification of the membrane suggested predominant presence of homomeric channels. l–n, HCN1 and HCN4 co‐expression in human (l). Single channel staining for HCN1 (DAB, m) and HCN4 (AEC, n) after color deconvolution showed single‐stained neurons for HCN4 with a large central axon initial segment (asterisk). o–r, HCN2 and HCN4 co‐expression in mice showed higher correlation of staining with partial overlapping fluorescent light (o). Single channel images for HCN2 (p) and HCN4 (q). r, High magnification of the membrane showed overlapping emission spectra (white color). The presence of heteromeric channels cannot be excluded here. s–u, HCN2 and HCN4 co‐expression in human showed a very heterogeneous distribution pattern (s). Single channel staining for HCN2 (DAB, t) and HCN4 (AEC, u) after color deconvolution. v–x, HCN fluorescent staining intensities from different sections in each cochlear turn were plotted as XY‐diagrams with its relative mean intensities of HCN1 versus HCN2 (v), HCN1 versus HCN4 (w) and HCN2 versus HCN4 (x)

Since few neurons were completely void of any immunoreactivity against one of the HCN channels, a co‐expression of several subunits within an individual neuron was obvious (Figure 6). SGN clusters that contained the highest staining intensities for HCN1, −2, and −4 were excluded from evaluation and only the staining of the “normal” separated neuron somata was quantified. Resolution was not sufficient to detect a co‐localization of the different HCN subunits in a heteromeric channel configuration. However, a lack of fluorescent overlap with conventional confocal imaging techniques provided information about the presence of non‐paired HCN combinations that appeared as single‐stained sites in double immunolabeling stainings. To assess proportional differences of HCN subunits in individual SGNs, the relative staining intensities in double staining combinations of HCN1‐HCN2, HCN1‐HCN4, and HCN2‐HCN4 were plotted as a combined XY‐histogram (Figure 6v–x). *τb* was calculated for each HCN combination and additionally for each cochlear turn. Colorimetric‐visualized immunostainings were done with human samples but not utilized for any quantitative analysis.

#### 3.2.1 HCN1–HCN2 co‐expression

Confocal optical sections gave no indication of any co‐localization of HCN1 and HCN2 (Figure 6a–d) in mice. Clearly separated positive pixels for both fluorochromes along the SGN soma membrane suggested no heteromeric pairing of HCN1 and HCN2 (Figure 6d). XY‐diagrams indicated similar proportions of HCN1 and HCN2 content in apical (*τb*_apical turn_ = 0.532, *p* < 0.001) and middle turn neurons (*τb*_middle turn_ = 0.522, *p* < 0.001), while this correlation was less pronounced in the basal turn neurons (*τb*_basal turn_ = 0.334, *p* < 0.001). HCN1 intensity had limited dependence on HCN2 intensity within the same basal neuron. The majority of HCN1‐rich cells (>60% relative HCN1 intensity) revealed the lowest HCN2 content (<60% relative HCN2 intensity) within a single neuron (Figure 6v). This is consistent with previous reports that HCN1 is emphasized toward higher frequencies (Liu, Manis, et al., 2014). Colorimetric staining in human apical to middle SGNs showed some membrane spots were more positive for HCN1 and some more for HCN2 within the same type I neurons (Figure 6e–g) suggesting a similar separation of HCN1 and HCN2 like in mice.

#### 3.2.2 HCN1–HCN4 co‐expression

Co‐expression of HCN1 and HCN4 in SGNs gave a similar picture to the HCN1/2 co‐staining (Figure 6h–k). HCN1 and HCN4‐positive puncta were well separated, indicating no presence of HCN1–HCN4 heteromers (Figure 6k). Relative staining intensities (Figure 6w) were similar for the apical to middle turn (*τ*_apical turn_ = 0.415, *p* < 0.01; *τ*_middle turn_ = 0.565, *p* < 0.001) but deviated for high‐frequency neurons (*τ*_basal turn_ = 0.145, *p* < 0.05) with no correlation to this HCN combination. This could be read as if most high‐frequency neurons comprised a basic level of HCN4 and only HCN1 varied. In human, similar trends with little variation in HCN4 and greater variability in HCN1 staining intensities in most neurons were observed (Figure 6l–n). Interestingly, we found type I neurons in the human middle turn that solely expressed HCN4. These neurons with high expression of HCN4 and low or negative staining for HCN1 were found in murine sections as well (data not shown), suggesting additional “specialized” subtypes.

#### 3.2.3 HCN2–HCN4 co‐expression

The co‐expression of these channels presented the strongest correlation coefficients for all turns, especially in the apex (Figure 6x: *τb*_apical turn_ = 0.729, *p* < 0.001; *τb*_middle.turn_ = 0.589, *p* < 0.001; *τb*_basal turn_ = 0.608, *p* < 0.001). This distribution indicated a mutual relationship of HCN2 and HCN4 and could have accounted for concerted functions, such as both subunits being modulated by cAMP. Fluorescent signals were less clearly separated in co‐staining experiments (Figure 6o–r). Most immunoreactivity sites revealed clearly separated fluorescent signals, but we also observed co‐localized pixels within spatial resolution limits. Hence, we cannot exclude some heteromeric HCN2/HCN4 channel combinations. In human, HCN2/HCN4 co‐staining resulted in most congruent color deconvoluted images in the apical turn (Figure 6s–u).

Overall, HCN1 dominated in high‐frequency neurons over HCN2 and HCN4. HCN2 and HCN4 co‐expression was mostly proportional. In both combinations of HCN4 with HCN1 and HCN2, we found single‐stained HCN4‐positive neurons in mouse and human. In HCN1–HCN2 double stainings, we found single‐stained neurons only in human sections indicating an even higher degree of specialized neurons.

### 3.3 Distinct pattern of HCN channel expression in mouse postnatal development

HCN channels revealed a distinct spatiotemporal pattern of expression in postnatal developing mice. There were no significant differences in HCN staining results in postnatal maturation between the C57Bl/6N and CBA/J mouse strains. We stained P1, P7, P9, and P16 mice to cover some important steps of hair cell innervation and refinement, and we present results of C57Bl/6N mice here. For comparative purposes, antibody dilutions and methods were equal to qualitative immunostainings in adult specimens.

#### 3.3.1 HCN1 and HCN3

**FIGURE 7:**
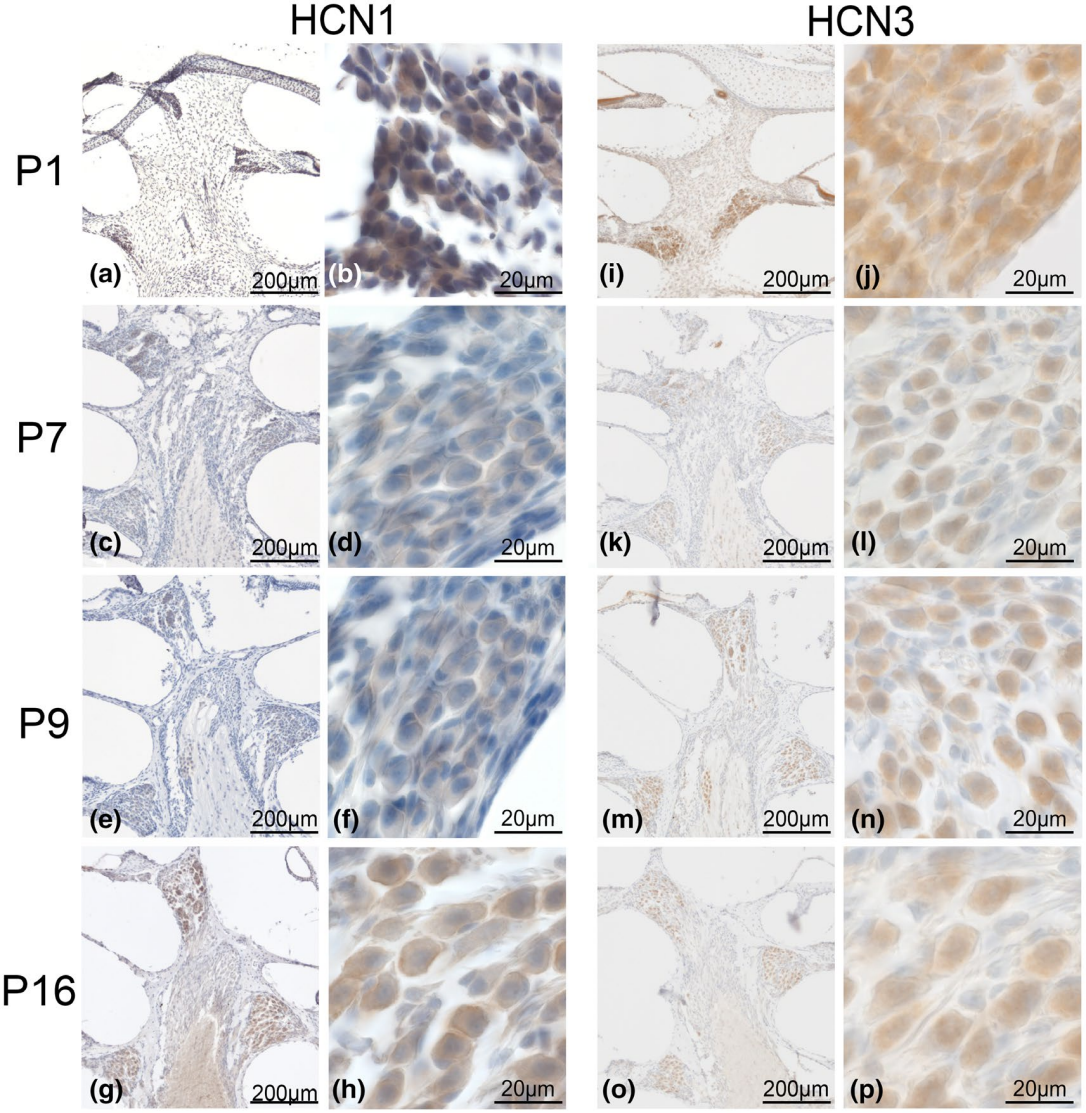
HCN1 and HCN3 postnatal expression until the onset of hearing. HCN1 and HCN3 were only visible at the neuron soma. Differences in the expression of these channels were evaluated during maturation from the first postnatal day until the onset of hearing at 16 days in C57Bl/6N. a–h, HCN1 expression in 1‐, 7‐, 9‐, and 16‐day‐old mice. a, b, HCN1 in P1 C57Bl/6N mice showed an apical–basal gradient (a). Staining in neuron soma (b). c, d, Apical–basal gradient was present at P7 (c), staining intensity decreased at the soma and HCN1 was found mainly at the membrane (d). e, f, At P9, the gradient of staining between different turns faded (e), and HCN1 was mainly present at the neuronal membranes (f). g, h, At the onset of hearing, the apical turn presented high expression in neuronal clusters (g) and overall intensity increased in the perisomatic membranes and the cytoplasm (h). i‐p, HCN3 expression at 1‐, 7‐, 9‐, and 16‐day‐old C57Bl/6N. i–j, at P1 no gradient for HCN3 between turns was observed (i), but high expression of HCN3 in the neuronal soma (j). k–p, no visible gradient was detected at P7 (k, l) or P9 (m, n) until onset of hearing (o, p) and the cytoplasmic staining decreases (P7, l; P9, n; P16, p)

Before the onset of hearing, HCN1 and HCN3 were only detectable in neurons but not in the sensory epithelium (Figure 7).

HCN1 showed intense staining at P1 in SGN soma membranes and in the cytoplasm, thus indicating high levels of protein production (Figure 7a,b). At later stages (P7–P9; Figure 7c–f), staining intensity decreased and localization shifted almost completely to the perisomatic membrane. Overall, staining intensity increased again until P16 (Figure 7g,h) with elevated HCN1‐LI in apical neuron clusters (Figure 7g). This resembled the expression pattern in the adult situation.

HCN3‐LI peaked at P1 in SGN cytoplasm (Figure 7i,j) and considerably decreased in all later stages (Figure 7k–p). Sensory epithelium staining was regarded as unspecific (Figure 7i). HCN3 immunoreactivity appeared homogenously distributed along the tonotopic axis. We were not able to find a distinct membrane staining.

#### 3.3.2 HCN2 and HCN4

We found HCN2 and HCN4 in SGNs and the sensory epithelium underneath the IHCs and OHCs as early as P1.

**FIGURE 8:**
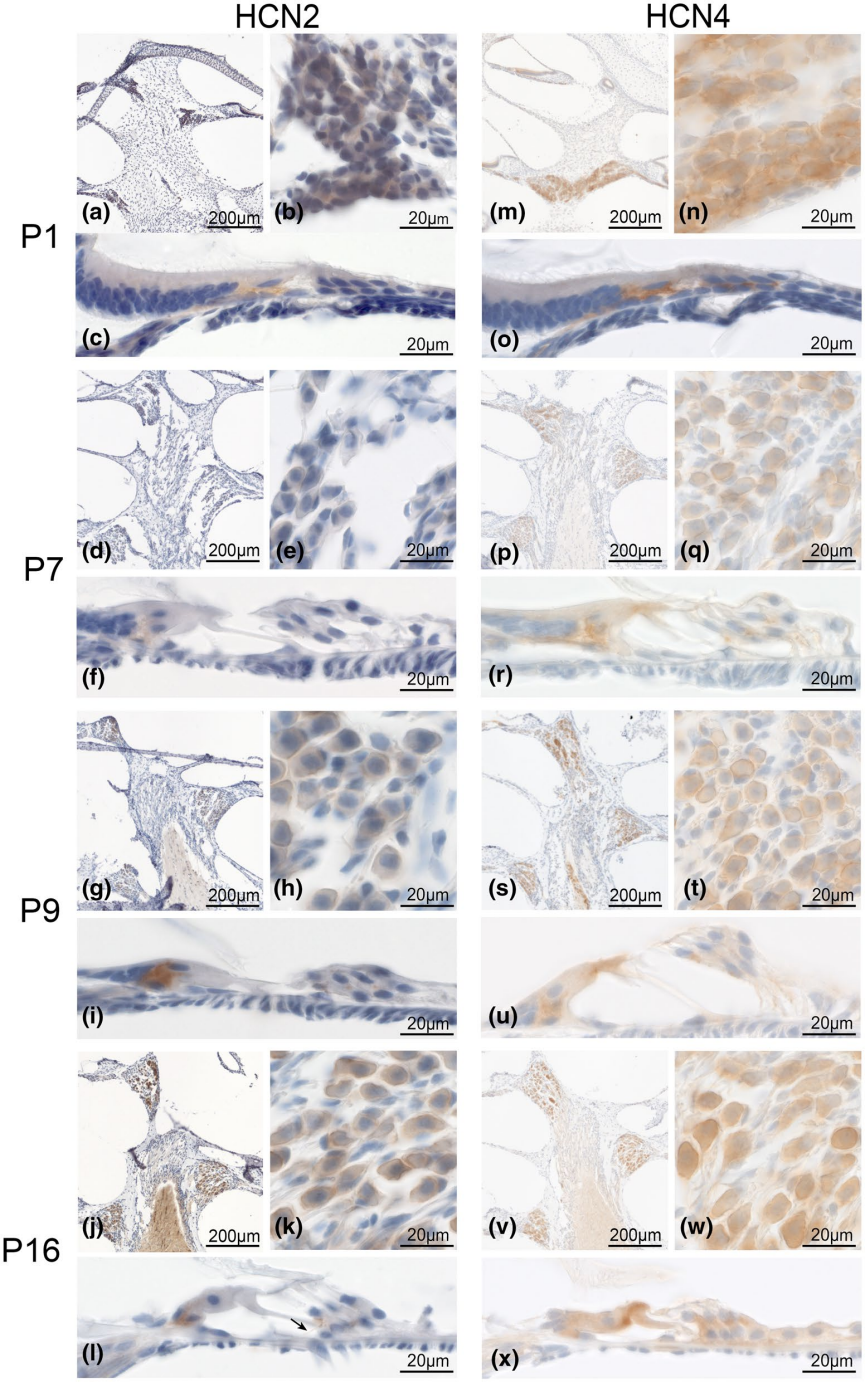
HCN2 and HCN4 postnatal expression until the onset of hearing. HCN2 and HCN4 were expressed in spiral ganglion neurons (SGNs) and the organ of Corti of C57Bl/6N mice as early as P1 and changed expression during maturation until onset of hearing. a–l, HCN2 expression at 1, 7, 9, and 16 days after birth. a–c, HCN2 was highly expressed at P1 without any visible tonotopical gradient (a). Staining located at the soma (b) as well as in the organ of Corti at afferent fibers underneath the inner hair cell (IHC), (c). d–f, At P7 the staining decreases in neurons (d, e) as well as sensory epithelium (f). g–i, at P9, neurons presented similar staining compared to P7 (g, h) but the intensity underneath the IHC increased and a faint staining underneath the outer hair cells (OHCs) appeared (i). j–l, around the onset of hearing, SGNs presented a more intense staining at the perisomatic neuron membranes compared to P9 and P7 (j, k). The now functional organ of Corti expressed HCN2 in putative type I afferents as well as in the efferent fibers underneath the OHCs (l). m–x: HCN4 expression in 1‐, 7‐, 9‐, and 16‐day‐old C57Bl/6N. m–o, High intensity staining of HCN4 was present at P1 at the soma membrane of SGNs in each turn (m–n). Prominent staining was visible underneath the IHCs and OHCs corresponding to afferent fibers at that developmental stage (o). p–r: at P7 a tonotopical gradient was visible with most intense reactivity in apical clusters (p). Staining intensity decreased at the neuron membrane (q) but prominent staining was still present in the afferent fibers of the organ of Corti (r). s–u, similar staining pattern was present in P9 neurons (s, t) and organ of Corti (u). v–w, after the onset of hearing, the tonotopical gradient faded with similar levels of staining in all turns (v), while overall staining in single neurons remained constant (w). No visible specific staining was detectable in the organ of Corti (x)

P1 HCN2‐LI (Figure 8a–c) was confined mainly to neuron somata and nerve fibers. We found intense staining especially underneath the IHCs (Figure 8c). Immunoreactivity appeared lower at P7 (Figure 8d–f) before it increased at P9 when this ion channel was located at neuronal perisomatic membranes and the ISP and, for the first time point, underneath the OHCs (Figure 8g–i). The latter location coincided with reports that first synapses between efferent nerve terminals and OHCs were seen at P9 (Shnerson et al., 1981). P16 mice (Figure 8j–l) allowed us to identify immunostained MOC‐TCFs (Figure 8l), and so immunostaining of SGNs visually increased (Figure 8k).

HCN4 showed a high‐expression level at P1 (Figure 8m–o) in neuron somata and underneath the IHCs and OHCs. Since there are no MOC fibers present at that stage of postnatal development (Bulankina & Moser, 2012; Shnerson et al., 1981), afferent type I and/or type II fibers are candidates. Initially, neurites of both SGN classes extend to all hair cell types (Huang et al., 2007). Neurite refinement and retraction and synaptic pruning eliminates type I SGN innervation of OHCs that extend to P6 (Huang et al., 2007). Hence, HCN4‐LI at P7 (Figure 8p–r) represented type I as well as type II neurons positively stained with type II nerve endings underneath the OHCs (Figure 8r). From P9 (Figure 8s–u) until the onset of hearing at P16 (Figure 8v–x) immunostaining increased in SGN somata (Figure 8t vs. 8w) but faded in the sensory epithelium (Figure 8u vs. 8x) of C57Bl/6N and CBA/J mice. Other species preserved HCN4 expression within the sensory epithelium into adulthood as reported above (Figure 5). Neuron clusters stood out with increased HCN4‐LI. Visually, no tonotopic gradient could be identified.

Newborn mice revealed intense immunostaining for all HCN channel types. HCN3 appeared to act more before birth and fade out after. HCN2 presented underneath the IHC at birth while HCN4 was additionally expressed at OHC afferents. HCN4 lost importance in the sensory epithelium until the onset of hearing function, and the faster kinetic HCN2 remained in IHC innervation and OHC efferents.

### 3.4 HCN expression in SGNs during aging and correlation with hearing thresholds

#### 3.4.1 eABR results

**FIGURE 9:**
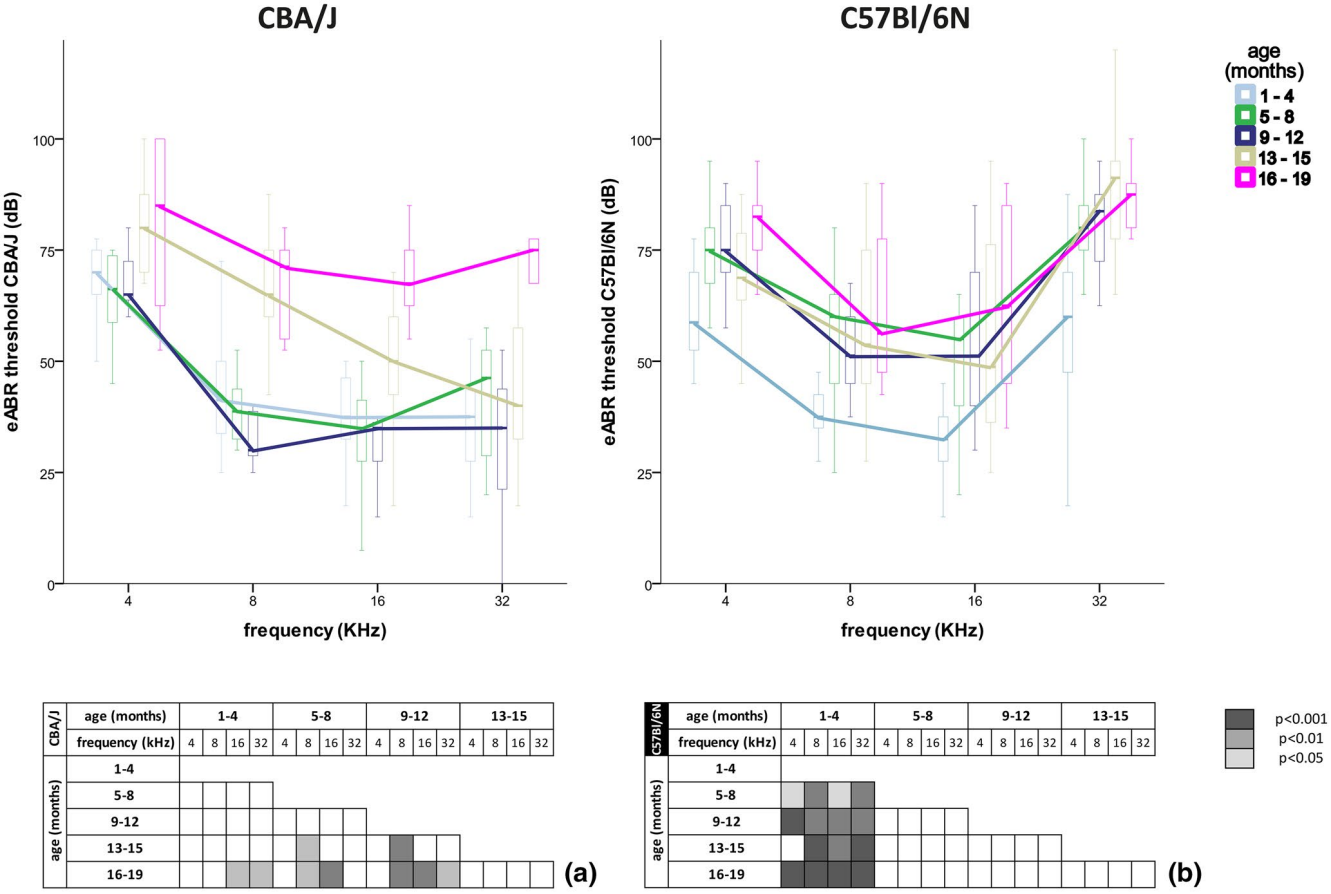
Hearing performance in C57Bl/6N and CBA/J with age. Evoked auditory brainstem responses (eABRs) thresholds of hearing for 4 kHz, 8 kHz, 16 kHz, and 32 kHz in CBA/J (a) and C57Bl/6N (b): tables underneath each figure represent the p‐value (Kruskal–Wallis test for nonparametric data and Dunn's post hoc test with Bonferroni correction, a, b).A value of*p* < 0.05 was considered as statistically significant, white represented no statistical difference

We confirmed the typical pattern of ARHL in our animals, with early elevated hearing thresholds of high frequencies in C57Bl/6N and of the low frequencies in CBA/J mice (Henry, 2002, 2004; Hunter & Willott, 1987; Sha et al., 2008). Significant differences are visualized in the tables below each graph (Figure 9). Hearing thresholds in CBA/J mice remained constant until 12 months before they increased at 8 kHz from 13 to 15 months and in 8–32 kHz in mice older than 15 months (Figure 9a). The 4‐kHz region did not show any statistically significant elevations of hearing thresholds across age groups. C57Bl/6N mice revealed high hearing thresholds from 8 to 32 kHz even in very young mice (1–4 months) (Figure 9c). Thresholds increased after 4 months across all frequencies, but were most prominent in the basal turn (Figure 9c). After 4 months, hearing thresholds remained relatively constant.

#### 3.4.2 HCN quantification

**FIGURE 10:**
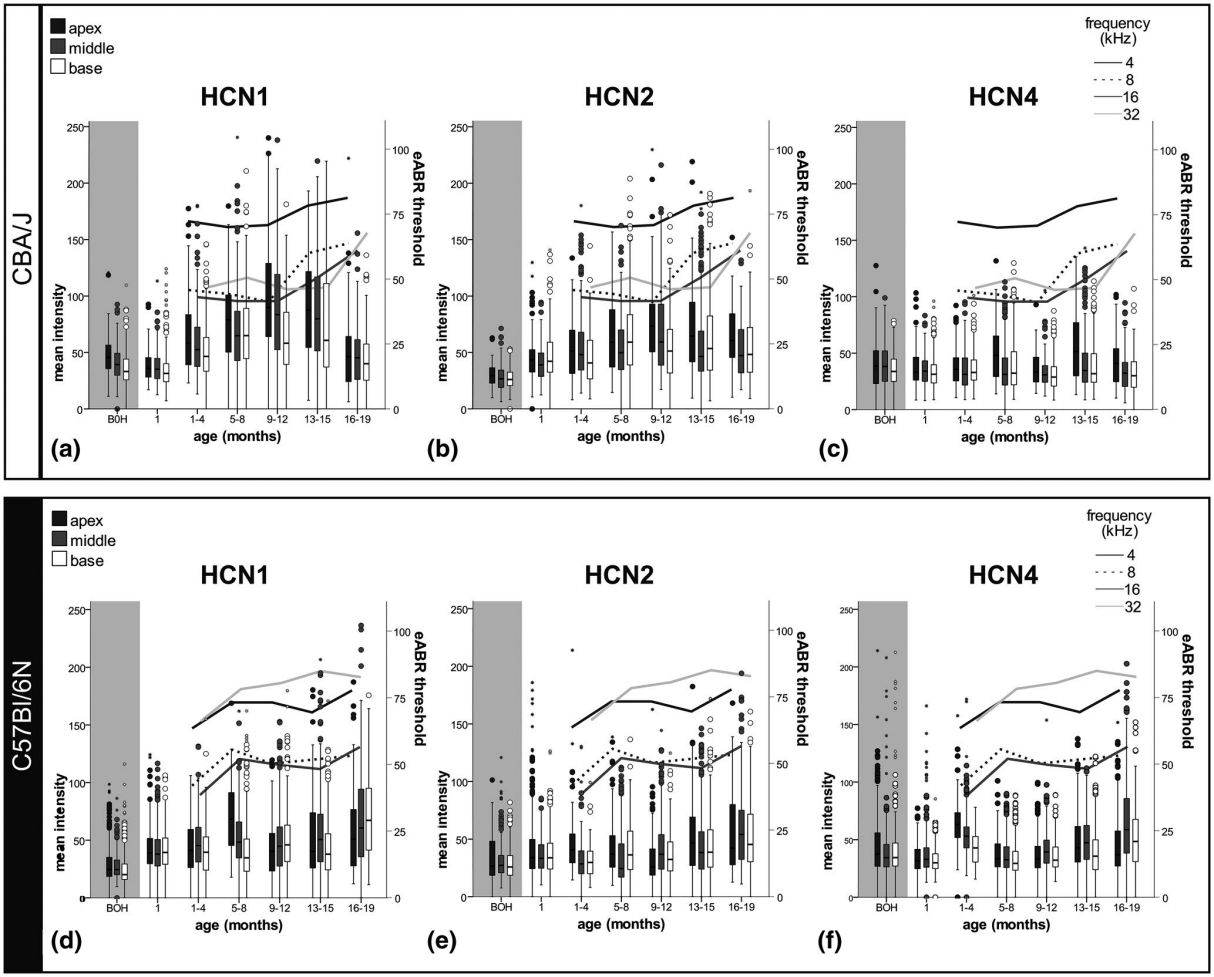
Differential expression of HCN channels in spiral ganglion neuron membrane with aging. HCN staining intensities at the perisomatic membrane of CBA/J and C57Bl/6N strains was semi‐quantified for HCN1, HCN2, and HCN4. Age groups spanned 3 months starting from the onset of hearing until 19‐month‐old mice. a–c, C57Bl/6N semi‐quantification of HCN1 (a), HCN2 (b), and HCN4 (c). d–g, CBA/J semi‐quantification of HCN1 (d), HCN2 (e), and HCN4 (f). Evoked auditory brainstem responses results are plotted as a second y axis representing pure‐tone hearing thresholds (compare to Figure 9). The nonparametric Kruskal–Wallis test and Dunn's post hoc test with Bonferroni correction was applied and*p* < 0.05 was considered statistically significant

**FIGURE 11:**
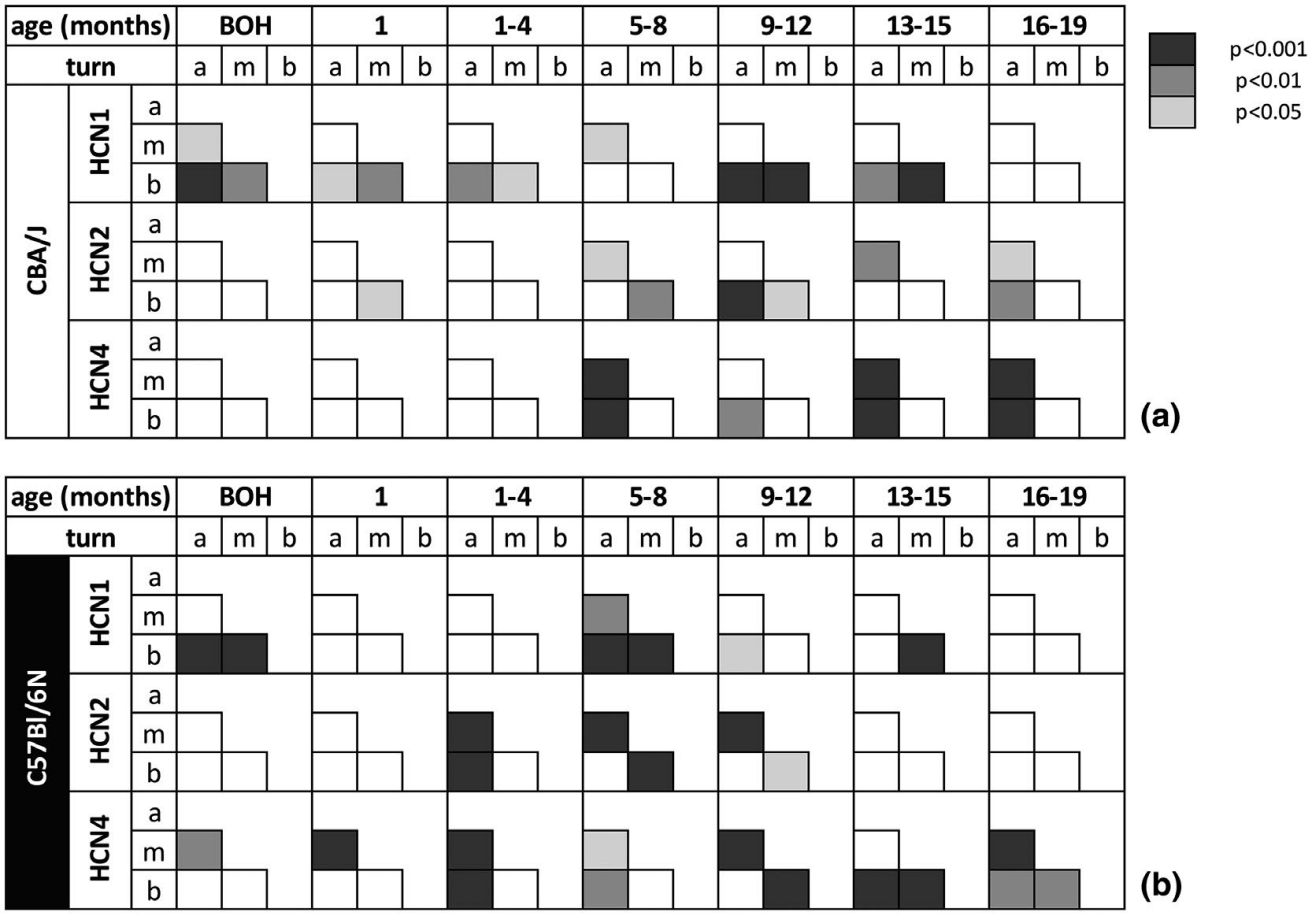
Graphical representation of statistical p‐value of HCN channel expression levels in SGN membrane in different cochlear turns for each age group. Representation of p‐values for CBA/J (a) and C57Bl/6N (b) of the significance of differences in HCN staining between the three cochlear turns (a, apex; m, middle; b, base). Kruskal–Wallis test was applied followed by Dunn's post hoc test with Bonferroni correction for pairwise comparison. BOH, before onset of hearing. P‐values are indicated in colors, white represents no statistical difference

**FIGURE 12:**
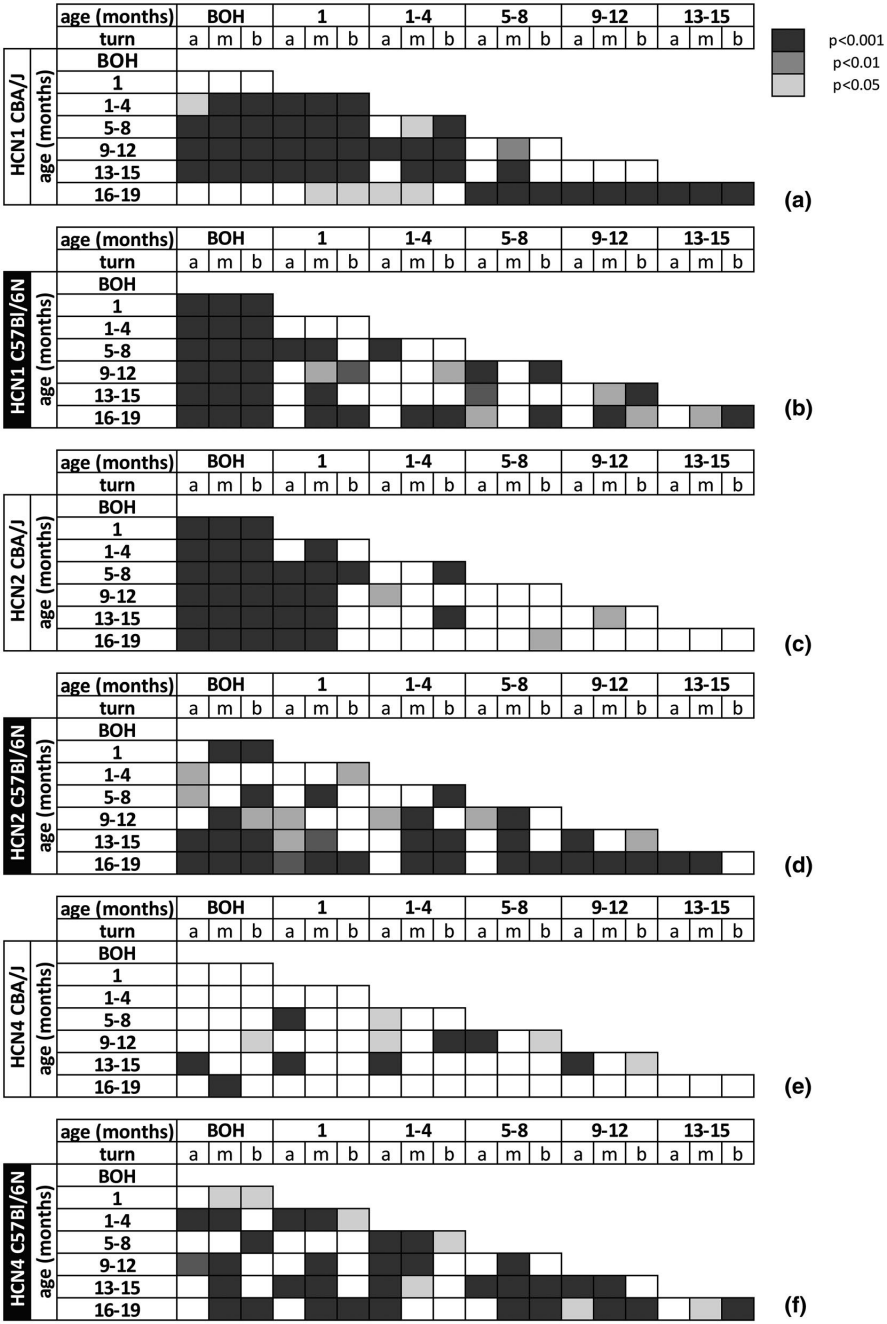
Graphical representation of statistical p‐value of HCN channel expression levels in SGN membrane with aging. Representation of HCN1 (a, b), HCN 2 (c, d), and HCN 4(e, f) p‐values of the significance of differences in HCN staining in different age groups of CBA/J (a, c, e) and C57Bl/6N mice (b, d, f) corresponding to Figure 10. The Kruskal–Wallis test was applied for comparison between the turns at different ages. Dunn's post hoc test with the Bonferroni correction was performed for pairwise comparison and p‐values are shown with a color code. White color code marked no statistical significance. BOH; before onset of hearing; a, apex; m, middle; b, base; p, p‐value

Semi‐quantitative evaluation of HCN1, −2, and −4 staining intensities from newborn to 19‐month‐old C57Bl/6N and CBA/J mice of individual SGNs revealed only a few corresponding trends with eABR recordings (Figure 10). Most noticeably, the intensity of immunoreactivity for HCN1 and HCN2 were higher in the “good hearing” CBA/J (Figure 10a,b) compared with C57Bl/6N (Figure 10d,e) mice, while HCN4 levels were similar. HCN1 was previously found to be expressed highest in the base and HCN4 in the apex in adult mice (Liu, Lee, et al., 2014). In contrast, our data showed that young adults up to 8 months of age present the highest levels of HCN1, −2, and −4 in low‐frequency neurons. SGN clusters were identified as hot spots of HCN expression and were frequent in the apical turn in C57Bl/6N but rarely found in CBA/J (Jyothi et al., 2010). Therefore, excluding clusters from evaluation would probably not have changed this gradient. Statistically significant differences are presented among the turns within one age group (Figure 11) and among the same turn at different ages (Figure 12).

##### HCN1

Across all age groups, HCN1 varied most in both mouse strains (Figure 10a,d). Variability of staining increased with HCN1‐LI upregulation, which was visible by the spreading of the boxplots with an increased data range (Figure 10a,d).

HCN1 presented in CBA/J a tonotopical gradient with higher levels in the apical turns previously in prehearing CBA/J mice (Figures 10a and 12a). After hearing onset, HCN1 raised expression until 9–12 months, remained stable until 15 months, and subsequently dropped (Figure 10a). The tonotopical gradient equalized in the eldest age group (Figure 11a). The marked ARHL in the 16‐ to 19‐month‐old group correspond to a massive loss of HCN1 immunostaining in CBA/J mice.

HCN1 peaked in C57Bl/6N mice in the apex in 5‐ to 8‐month‐old animals, a time point when the biggest threshold elevations appeared in the 4–8 kHz frequency range (Figure 9c) and subsequently remained constant until 15 months of age. However, contrary to CBA/J mice, the C57Bl/6N mice older than 15 months responded with an elevation of HCN1‐LI intensity in mid‐to‐high frequencies (Figures 10d and 12b). The tonotopic gradient of HCN1 expression in C57Bl/6N mice was less evident and even reverted in animals older than 8 months (Figure 11b).

##### HCN2

CBA/J mice reached an early plateau of intensity after 4 months of age (Figure 10b).

Immunostaining intensities in C57Bl/6N mice changed mainly in the middle and basal turns (Figures 10e and 11e). While there were no significant changes of HCN2 expression until 4 months, staining intensities tended to increase in mice older than 1 year of age. This coincided with an increase in hearing thresholds after 4 months of age. Diversity of individual expression levels of HCN2‐LI represented by the spread of data (Figure 10e) was less pronounced.

##### HCN4

HCN4 is the most stable expressed subtype and little influenced by age or hearing performance. We observed a few marked changes mainly in the apical turn in CBA/J mice (Figure 10c). Immunoreactivity peaked in 5‐ to 8‐ and 13‐ to 15‐month‐old groups. The drop of HCN4‐LI in the basal turn of 9–12‐month‐old‐mice was statistically significant compared with the flanking age groups (Figure 12e). C57Bl/6N individuals showed the most significant differences in the apical and middle turn (Figures 10f and 12f) but not any consistent tonotopical gradient across life span. HCN4‐LI intensity peaked in the 1‐ to 4‐month‐old group in all turns. After a decline in staining of the 5‐ to 8‐month‐old group, HCN4‐LI increased in the middle and basal turns.

In summary, HCN1 intensities varied the most with aging and showed a bell‐shaped rise–fall characteristic in CBA/J, while HCN1‐LI increased with advanced age in C57Bl/6N mice. HCN2 expression followed a sigmoidal curve and stabilized after 4 months in CBA/J mice. HCN2 immunoreactivity increased in C57Bl/6N mice similar to HCN1. HCN4 showed the lowest variability across all age groups, and oscillated expression levels in CBA/J mice in the apex, while it followed trends similar to those in HCN1 and HCN2 in C57Bl/6N mice.

This pattern of HCN expression presented only a weak correlation to the different ARHL types of the two mouse strains. Especially the opposing behavior of up‐ and downregulation of HCN1 and HCN2 intensities at very old age appeared to be obscure since both mouse strains exhibited marked hearing loss in the 16‐ to 19‐month‐old group. The data did not allow us to extract a common trend in HCN expression in aging mice, since the changes were too varied and showed little correlation with the pure‐tone threshold audiometry data. All immunostaining results were summarized in Table 3.

**TABLE 3:** Summary table of the HCN channel distribution in mouse (C57Bl/6N, CBA/J, and Ly5.1), guinea pig, cat, and human cochleae

HCN	Species	SGN I soma mb	SGN I cytop	Grad	pAIS	cAIS	SGN I terminals	SGN II soma mb	SGN II terminals	Efferents	Hair cells	Ageing
1	mouse	+	+	apical‐basal CBA, Ly5	–	+	–	−B6, −CBA, −Ly5	–	–	–	CBA ʌ
	gp	+	+	No grad B6	–	+	–	+*	–	–	–	
	Cat	+	+		–	+	–	+	–	–	–	
	human	+	+		–	+	–	+	–	–	–	B6 ↗
		++SGN cluster										
2	mouse	+	+	apical‐basal CBA, Ly5, B6	–	–	+	−B6, −CBA, −Ly5	−B6, −CBA, +Ly5	+	–	CBA ↗
	gp	+	+		–	–	+	+*	+	+	–	
	cat	+	+		–	–	+	+	+	+	–	
	human	+	+		–	–	+	+	+	+	–	B6 ↗
		++SGN cluster								MOC		
										other		
										LOC?		
3	mouse	–	+	apical‐basal B6	–	–	–	+B6, +CBA, +Ly5	–	–	–	Not quantified
	gp	–	+	CBA?, Ly5?	–	–	–	+*	–	–	+OHCs	
	cat	–	+		–	–	–	+	–	+OSL, CN	+OHCs	
	human	–	+		–	–	–	+	–	–	–	
4	mouse	+	+	apical‐basal B6	+	+	+	−B6, −CBA, +Ly5	+B6 & CBA only prehearing, Ly5?	–	–	CBA →
	gp	+	+	No grad CBA	+	+	+	+*	+	–	–	
	cat	+	+	Ly5?	+	+	+	+	+	–	–	
	human	+	+		+	+	+	+	+	–	–	B6 →
		++SGN cluster					++B6, CBA					
							Prehearing					

Abbreviations: B6, C57Bl/6N; cAIS, central axon initial segment; CBA, CBA/J; CN, central cochlear nerve; cytopl, cytoplasm; gp, guinea pig; grad; gradient; LOC, lateral olivocochlear efferents; Ly5, Ly5.1; mb, membrane; MOC, medial olivocochlear efferents; N/A, not available; other, other efferent innervation; pAIS, peripheral axon initial segment; term., terminal; –, no HCN staining; +, positive HCN staining; intensity profile during aging: ʌ rise‐fall, ↗increase, → constant.

* According to report of (Bakondi et al., 2009).

## 4 DISCUSSION

### 4.1 Are HCN channels in all mammalians distributed the same?

We found that HCN channel subunits were distributed unevenly among mammalian auditory neurons and showed characteristic changes of expression levels during postnatal development and aging. Expression patterns were not uniform among species or even within mouse inbred strains. This variational expression may represent evolutionary adaptions or loss of functions. Despite largely overlapping expression patterns, individual neurons were distinct based on the subcellular location of the four ion channel subunits which significantly determine HCN function (Nusser, 2012). Interspecies deviations of HCN localization may affect possible functions of HCN channels.

#### 4.1.1 HCN channels at the peripheral nerve endings

The combination of the highly specialized ribbon synapses with glutamatergic neurotransmission ensures quick and reliable sensory transduction to accurately preserve timing information of sound. Dendritic HCN channels shape excitatory postsynaptic currents (EPSCs) by avoiding summation of signals (Yi et al., 2010). This keeps EPSCs brief and allows the maintenance of high EPSC frequencies but is bought by a potential reduction in EPSC amplitudes. That may be one reason for the very strong currents initiated at IHC synapses (Grant et al., 2010), which has been supported by findings that large EPSCs enhance the precision of spike timing (Li et al., 2014). A fast instantaneous component of *I*_h_ observed with pulsed hyperpolarization was found to grow maximal in HCN3 and HCN4 channels that presented the slowest activation kinetics. This pre‐hyperpolarization enhancement was determined to be more significant at higher frequencies of hyperpolarization which may be caused by incomplete deactivation of HCN4 (Mistrik et al., 2006), representing one way to reduce AP rates. The loss of HCN4 in adult CBA/J and C57Bl/6N mice but not in Ly5.1 mice and other species likely represents a loss of this putative function.

#### 4.1.2 Somatic HCN channels

Most of the HCN channel protein content was located at the perisomatic membrane of SGNs. The main function of somatic HCN channels is to control the RMP. A fraction of HCN channels is tonically open at rest. In SGNs, 20% of *I*_h_ current was estimated to be active at the RMP (Kim & Holt, 2013). This actively stabilizes the RMP leading to bigger voltage responses through a decreased input resistance (He et al., 2014; Kim & Holt, 2013). The bigger the size of a SGN soma and the more HCN channels that are open at rest, the lower this input resistance will be. This membrane shunting effect supports fast‐rising and precisely timed synaptic events (Baumann et al., 2013; Khurana et al., 2012) and may facilitate auditory signal transmission across the big soma through a “depolarized RMP.” The soma of bipolar neurons increases the capacitance that theoretically decreases amplitudes and delay signal propagation between peripheral and central axon as we modeled previously (Rattay et al., 2013). Acute block of *I*_h_ resulted in RMP hyperpolarization (Kim & Holt, 2013). HCN channels generate an inward current and can even depolarize a neuron. Since there are no voltage‐gated sodium (Na_v_) channels located at the soma membrane to carry the depolarization current across the neuron cell body (Hossain et al., 2005), HCN channels may be the candidates to overcome this burden for APs. SGNs with highest HCN expression levels may be specialized to convey the most precise temporal information of sound. Our staining results showed that these most intense immunolabeled neurons were evenly distributed within a tonotopic region in all species we investigated. This indicates that there are no tonotopic hot spots for temporal acuity but many IHCs receive innervation from these high HCN level neurons. SGN clusters were the exception in this observation. Co‐expression of several mainly homomeric HCN subtypes with different gating characteristics may further contribute to SGN diversification at the level of the soma. Species such as human (and cat) revealed the most distinct patterns with neurons expressing only a certain HCN subtype. This suggests a higher grade of specialization of SGNs in humans compared to rodents.

#### 4.1.3 SGN somata clusters

Tight association of two to eight neuron cell bodies into SGN clusters are very typical in humans and are frequently seen in some strains of mice. This is almost exclusively associated with a lack of myelin around the soma. Missing somatic myelination is the rule in humans, as only a few neurons contain a thin myelin layer (Arnold, 1987; Potrusil et al., 2012). In contrast, most other mammals reveal fully myelinated auditory neurons. Our immunostaining identified auditory neuron clusters as HCN hot spots containing the highest amounts of HCN1, HCN2, and HCN4, with the most intense staining at adjoining membranes. Recently, the presence of HCN channels was found to alter the phase but not the amplitude of local field potentials (Sinha & Narayanan, 2015). Elevated amounts of these channels may boost ephaptic coupling between adjoining cells and are involved in synchronization of the AP firing in Purkinje neurons (Han et al., 2018). This ephaptic coupling may be favored by the absence of an insulating myelin sheet. In SGNs, a preprocessing of phase characteristics may be especially valuable for the low‐frequency phase‐locking coding of sound. Why some animals or mouse strains present these neuron clusters frequently and others do not is unclear and will require further comparative studies. A complete lack of somatic myelination in humans favors the importance of ephaptic phase synchronization and may represent more of an evolutionary adaption than something “pathological” as suggested for the Ly5.1 mouse strain.

#### 4.1.4 HCN channels in pre‐ and postsomatic segments

The AIS located within the postsomatic segment is regarded as the AP initiation site for neurons, containing a tightly ordered cytoskeletal structure with highest densities of Na_v_ and voltage‐gated potassium (K_v_) channels. It determines the lowest firing thresholds and improves the temporal precision of spike coding (Lazarov et al., 2018). Selective blocking of somatic HCN channels resulted in a decrease in spike probability while site‐selective HCN channel blocking in the AIS increased spike probability. We have found distinct patches of HCN1 and HCN4 in the pre‐ and postsomatic segments that do not exceed cAIS and pAIS distal endings. The fast kinetic HCN1 may prevent hyperexcitability and control the RMP at the cAIS, avoiding continuous firing in response to small depolarizations (Ko et al., 2016). HCN4 in the cAIS and pAIS could favor strong depolarizations and suppress lower AP frequencies through the effect of prehyperpolarization enhancement at higher frequency of hyperpolarization (Mistrik et al., 2006). We found HCN1 and HCN4 co‐expressed within the same postsomatic segment. The double action of very different timing and activation characteristics of HCN1 and HCN4 at the postsomatic segment likely shape the spike timing and pattern of SGNs in an individual way and may allow them to respond differently according to the input AP frequency. We found the longest and most pronounced HCN4 stainings of postsomatic segments in human SGNs especially in the apical turn. This provides further evidence for the advanced diversification of SGN subtypes in humans. Since timing information is most critical in phase locking coding of low frequency hearing, an AIS with marked HCN4 content may be important for extracting timing information of complex speech.

#### 4.1.5 HCN channels in type II SGNs

The small type II neurons with their thin nerve fibers differ considerably from the much bigger type I neurons in that they have a complete lack of myelination at the soma and axons. Type II afferent nerve endings of OHCs present the glutamatergic ribbon type synapses like in type I neurons, but OHCs typically release only single vesicles with low probability (Weisz et al., 2009). It is supposed that a single OHC does not trigger an AP but summation of at least six OHCs is necessary to reach the threshold (Weisz et al., 2014). Peripherin‐positive neurons showed different HCN subtype expression. We found all HCN subunits in human and cat, which is consistent with data in guinea pig (Bakondi et al., 2009). Adult C57Bl/6N and CBA/J mice only presented HCN3‐LI in type II neurons. These inbred mice strains may have lost substantial expression levels of HCN channels in type II neurons and should not be used to study the influence of HCN channels in these types of neurons. Dendritic type II location may function similar to type I neurons but at a “slower” level. Type II peripheral axons were reported to contain Na_v_ 1.6 within their course in the organ of Corti (Hossain et al., 2005) which corresponds to our HCN4 and HCN2 staining pattern. As a concerted stimulation of several OHCs follows a summation of EPSCs until threshold is reached, an AP would be triggered anywhere along the type II peripheral process within the sensory epithelium. HCN channels could open only at these AP initiation sites and avoid temporal summation. Also, modulatory factors such as cAMP could alter AP initiation and tune type II response.

Different subpopulations of peripherin‐positive type II neurons were previously found in humans including a few myelinated (Arnold, 1987; Rattay et al., 2013) that could convey APs up to 4.5 faster than the classical unmyelinated type II neurons (Rattay et al., 2013). The function of these fast type II neurons remains unknown. Higher heterogeneity in human type II SGNs may be associated with higher diversity of HCN subtype expression.

#### 4.1.6 HCN channels in efferent nerve fibers

We found only HCN2 in the efferent MOC fibers innervating the OHCs. *I*_h_ is partially activated at rest, and together with low threshold K_v_ (KLT) and high threshold K_v_ (KHT) channels, HCN channels may act in repolarizing the axonal membrane thereby shorting APs. This may enable a higher AP frequency for the very thin caliber efferent fibers. As HCN2 channels are sensitive to pro‐inflammatory molecules following a cAMP rise (Emery et al., 2012), these mediators may influence the maximum AP frequency and tune MOC response at the level of the sensory epithelium. A cAMP rise may lead to a reduced cochlear amplification and could thereby help to protect the sensory cells from further damage over longer time periods. All investigated species showed this expression pattern.

#### 4.1.7 HCN channels in hair cells

We located HCN channels mainly in neurons. HCN3 was solely present in the lateral membrane of OHCs in guinea pigs and cats and probably in humans. Membrane preservation may have been insufficient for a clear detection in human postmortal tissue. This location of an HCN channel was not reported previously. We could not confirm previous reports about the presence of HCN1 and HCN2 channels in auditory hair cell stereocilia (Ramakrishnan et al., 2009, 2012). However, other authors also did not find HCN subunits in hair bundles (Horwitz et al., 2010), but HCN1 was found at the basolateral membrane of utricular hair cells (Horwitz et al., 2011). Our findings identified HCN3 at the lateral cell membrane of OHCs overlapping with prestin location, the protein responsible for OHCs motility (Zheng et al., 2000). HCN3 could add a voltage‐gated cation conductance close to the prestin molecules. Chloride (Cl^−^), which is important for the conformational change and function of prestin, is also known to regulate HCN channels extracellularly (Frace et al., 1992). As mentioned above, the fast component of *I*_h_ grows maximally in slow HCN channels such as HCN3. Repeated hyperpolarization led to additive but quickly saturating enhancements of the fast component of *I*_h_. However, an increase of intracellular Cl^−^ concentration suppressed this effect (Mistrik et al., 2006). A lowering of the Cl^−^ concentration increased conductivity, thereby changing electrical properties at the lateral membrane of OHCs. How and if HCN3 directly interplays with prestin needs more functional studies, but the remarkable expression site overlap and common modulators suggested some involvement. Since a hyperpolarization is the trigger for HCN channels to open and increase conductivity across a cell membrane, an activation of HCN3 in the lateral membrane appears plausible. *I*_h_ may also actively counteract deviations of the membrane potential to support fast repolarizations. We could not detect HCN3 in C57Bl/6N, Ly5.1, or CBA/J inbred mouse strains, so the animal model to study HCN involvement in OHC function may be crucial.

### 4.2 HCN tonotopical gradients and co‐expression in neurons

HCN channels are not uniformly dispersed along the tonotopic axis. Gradients depend on HCN subtype, mouse strain, and age. In most situations, apical neurons displayed higher expression for HCN1 but also high levels of HCN2 and HCN4 channels, contrary to earlier research that indicated HCN1 was more expressed in basal neurons (Liu, Manis, et al., 2014). The authors described a more positive *V*_1/2_ for *I*_h_ in the apex. That could be explained by high levels of HCN1. Apical neurons with highest HCN protein expression and highest correlation for HCN1 and HCN2 may most strictly control timing precision to maintain their phase‐locked sound coding that sets formidable demands. Moreover, binaural cues that utilize a submillisecond interaural time difference for sound localization largely depend on lower frequency neurons (Ison et al., 2017; Kim & Holt, 2013).

The most densely innervated and most sensitive frequency region of the middle turn is characterized by an even composition of neuronal HCN subunits. A stricter control of HCN balance may be an adaption to communication skills as the middle frequencies often serve this purpose in mammals. Most neuron subtypes differ mainly in their HCN expression levels and not in the relative composition of subunits. The situation is different in the basal turn, where co‐expression experiments indicated that increasing levels of HCN1 were almost independent of the presence of HCN2 and HCN4. This qualifies HCN1 to be most dominant in high‐frequency neurons. Loss of HCN1 in mice resulted in elevated hearing thresholds only in high frequencies (24–48 kHz) (Ison et al., 2017) and indicated that HCN1 deficiency may not be compensated in basal neurons. The very patchy distribution of HCN subunit spots along the SGN somatic membrane may turn patch clamp studies on HCN channels into a lottery game if hundreds of neurons or patches are not analyzed. The dominance of single‐stained patches in double staining experiments allowed us to infer that HCN heteromers may not play a prevalent role compared to HCN homomeric channels. Only HCN2 and HCN 4 showed tighter spatial association and quantitatively the best correlation, so some heteromeric HCN2/HCN4 compositions may be present at auditory neuron somata. More heterogeneity is seen in humans, as several individual neurons predominantly expressed one of the subunits. This phenomenon was not restricted to a certain frequency region and may represent a higher grade of specialization of SGNs across the entire frequency spectrum of hearing.

### 4.3 Prehearing postnatal HCN channel expression in mice

At perisomatic membranes, HCN channels control the RMP (He et al., 2014) which is directly related to the rate of intrinsic firing in postnatal neurons (Lin & Chen, 2000). Spontaneous firing activity of SGNs promotes their survival and maturation (Babola et al., 2018). These APs are a result of spontaneous Ca^2+^ spikes in immature hair cells initiated by the release of ATP from the glia‐like inner supporting cells (ISCs) via the release of Cl^−^ ions (Tritsch & Bergles, 2010; Tritsch et al., 2007) and start around P10 (Ehret, 1976). Interestingly, HCN2 and HCN4 showed increased *I*_h_ conductance with increasing external Cl^−^ concentration (Frace et al., 1992). Kim and Holt (2013) demonstrated that HCN1 and HCN2 produce *I*_h_ at early postnatal stages. HCN4 could not be tested due to embryonic lethality of the HCN4^−/−^ mice, but was also suggested to be present. These data support the idea that HCN1, −2, and −4 are active components at neonatal stages. The most intense staining of HCN3 in P1 mice indicated a more relevant role of the channel prior to birth. Our data conform with previously reported expression ratios in early postnatal mice SGNs that identified HCN3 as the least expressed subtype (Kim & Holt, 2013). Recently, spontaneous activity was found to be related to HCN channels in neurons of the cochlear nucleus from prehearing mice (Yin et al., 2018). Spontaneous activity plays a major role in maturation and refinement of auditory circuit formation (Blankenship & Feller, 2010; Frank & Goodrich, 2018). As the slow kinetic HCN4 decreases in expression considerably in adults, this ion channel may contribute much to lower firing rates in prehearing mammals. Prehearing rodents showed spike rates (SRs) of few spikes/s (rat P0–P9, SR < 5 spikes/s (Tritsch & Bergles, 2010); rat P15–P17, SR < 16 spikes/s; P19–P21 SR < 44 spikes/s; and P29–P32 SR < 55 spikes/s (Wu et al., 2016) increasing in vivo up to 120 spikes/s in 4‐ to 12‐month‐old rats (el Barbary, 1991). Prominent intensifying of staining of HCN2 at P9 underneath the IHC may mark the transition from low to higher SR activity. Our knowledge about afferent–efferent circuit formation and refinement is still very patchy, but HCN channels could play a role in these activity‐dependent mechanisms. The transient expression of HCN2 and HCN4, underneath IHCs and OHCs, suggests at least a supportive function to maintain spontaneous “pacemaker” activity in an extensive remodeling period in mouse auditory development.

### 4.4 HCN channels in aging

The puzzling mixture of factors that are putatively causative for or associated with the aging process in neurons include a plethora of sources, such as elevated oxidative stress, homeostatic dysregulation, DNA damage, protein aggregation, and impaired metabolic function as well as many other factors together with genetic predispositions and environmental influences (Mattson & Magnus, 2006). HCN channels were recently identified to be upregulated with aging (Shen et al., 2018). Our C57Bl/6N data on the upregulation of HCN1 and HCN2 and a few changes for HCN4 in ARHL largely confirm their observations. Ups and downs in HCN1 and HCN2 expression levels and considerable differences between mouse strains exposed age‐related HCN expression to be much more complex and that selection of age groups may be critical. In contrast to Shen et al. (Shen et al., 2018), we could not find relevant expression levels of HCN3. In their study, elevated expression levels of HCN1 in C57Bl/6N mice resulted in increased AP numbers in the basal turn. The spike probability enhancement by HCN upregulation may be involved in a feedback mechanism to combat sensorineural decline especially in the high‐frequency region in C57Bl/6. There is a considerable loss of neurons at older ages in all turns (Shen et al., 2018), so HCN1/2 content is upregulated in the neurons that survive. Lowering the threshold of spiking through tonically open HCN channels and enabling fast RMP recovery after an AP may indicate that high sensitive SGNs survived better than low sensitive neurons and even increased their spiking rate.

The situation was very different for the “good hearing” CBA/J mice that retained better hearing performance with advanced age. The 15‐month‐old mice had a considerable threshold shift throughout all tested frequencies but showed a decrease in HCN1 and HCN2‐LI. CBA/J mice started losing hair cells in the apex accompanied with only a modest loss of neurons (Sha et al., 2008). Since CBA/J mice showed only a mild loss of cochlear neurons (Liu et al., 2019; Ohlemiller et al., 2010), the peripheral input may have been sufficient and an increase in hyperexcitability or enhanced spontaneous activity would not have been induced. HCN1 and HCN2 downregulation may result in a more hyperpolarized RMP, and that would decrease spike activity. This could break the vicious circle of upregulated activity with a loss of sensory input. Hair cell loss may account primarily for this reduced activity, but HCN downregulation could contribute. C57Bl/6N mice seemed to compensate for the loss of sensory cells with hyperexcitability of remaining SGNs that made these neurons likely to be more vulnerable to damage. Measurements of *I*_h_ in old and young C57Bl/6N mice did not show any changes in *V*_1/2_ or slope factors of *I*_h_ (Shen et al., 2018) suggesting that the overall proportions of the HCN channel set was unchanged. In both mouse strains, HCN1 and HCN2 channels may be important regulators of SGNs excitability with aging, whereas the constantly expressed HCN4 subtype does not seem to be very involved.

Although not quantified, Ly5.1 presented the highest intensity of the staining for all the HCN subunits, suggesting that this strain is suitable for further physiological examinations. The non‐myelinated SGNs also facilitated electrophysiological experiments, and the SGN anatomy was closer to the human situation.

### 4.5 HCN channels and electrical stimulation with cochlear implants

Cochlear implants (CIs) rely on excitable neurons that can be stimulated through a pulsed electrical field. Electrical stimulation (ES) of SGNs differs considerably from acoustic stimulation, as CIs directly excite voltage‐gated ion channels and bypass the IHCs. Under acoustic stimulation, SGNs reveal a wider dynamic range, show higher variability in firing rates, and undergo weaker phase locking (Boulet et al., 2016; Hartmann et al., 1984; Javel & Viemeister, 2000). Previous work highlighted the importance of considering temporal properties in order to improve CI performance (Boulet et al., 2016). Stimulus‐response phenomena produced in type I SGNs, such as refractoriness, facilitation, accommodation, and spike rate adaptation, cannot fully be explained with adapted Hodgkin–Huxley models (Mo & Davis, 1997; Negm & Bruce, 2014). Since HCN channels are considerably involved in shaping the timing of spiking, it seems likely that they also influence the neuronal responses evoked through ES, especially when the stimulation frequency is high. Recent modeling studies have suggested HCN channels to be actively involved in these phenomena, especially at high stimulation rates (Negm & Bruce, 2014). This was comprehensively reviewed recently (Boulet et al., 2016). However, the benefit of high stimulation rates of several thousand pulses/s to improve speech understanding is still under debate (Boulet et al., 2016; Kiefer et al., 2000; Loizou et al., 2000; Wilson et al., 1988). It is not yet fully understood why some CI recipients perform better with higher stimulation rates while others do not. Together with KLT channels, HCN activity can extend the neuron refractory period. Subthreshold pulse trains may reduce the number of open HCN channels and contribute to a reduced excitability (accommodation). HCN channels have also been proposed to be involved in spike rate adaptation through accumulating after hyperpolarization (Negm & Bruce, 2014). Pulsed hyperpolarization that grows maximal in HCN4 channels at higher frequencies (Mistrik et al., 2006) may account for spike rate adaptation with ES.

### 4.6 Conclusions

HCN channel spatiotemporal expression and localization, together with previously published physiological data, provide plenty of data to explain possible functions of this group of ion channels in the peripheral auditory system. HCN channels are present early in sensorineural circuit formation and possibly contribute to control neuronal spontaneous activity in a critical phase of hair cell innervation and refinement. HCN channels actively tune AP frequency and control summation effects in type I and type II neuron afferent nerve terminals but also influence efferent connections to hair cells and supporting cells. HCN activity very likely enhances ephaptic coupling of SGN clusters, thereby synchronizing groups of SGNs at the level of the cochlea. The presence of these voltage‐gated channels at efferent fibers may influence feedback loops and tune the response to pro‐inflammatory situations. Age‐related changes are diverse as HCN channels may be involved in increasing and decreasing excitability of neurons depending on animal species or type of hearing deterioration. The newly found unknown involvement of HCN channels in OHC function exposes the underestimated influence of HCN channels for auditory function. A better understanding of HCN action may open up new possibilities to tune neuronal responses evoked through ES by CIs.

## DECLARATION OF TRANSPARENCY

The authors, reviewers and editors affirm that in accordance to the policies set by the *Journal of Neuroscience Research*, this manuscript presents an accurate and transparent account of the study being reported and that all critical details describing the methods and results are present.

## CONFLICT OF INTEREST

There is no conflict of interest to declare.

## AUTHOR CONTRIBUTIONS

M.L, A.S‐F, and R.G had full access to all the data in the study and take responsibility for the integrity of the data and the accuracy of the data analysis. *Conceptualization,* M.L. and R.G.; *Methodology,* M.L., R.G. and J.D.; *Validation,* W.L. and H.R.‐A.; *Investigation,* M.L. and R.G.; *Formal Analysis,* M.L. and R.G.; *Resources,* A.S.‐F., E.P., E.B., H.R.‐A. and R.G.; *Writing – Original Draft,* M.L. and R.G., *Writing – Review & Editing,* M.L., A.S.‐F., H.R.‐A., J.D. and R.G.; *Visualization,* M.L. and R.G.; *Supervision and Project Administration,* R.G.; *Funding Acquisition,* R.G. and A.S.‐F.

### PEER REVIEW

The peer review history for this article is available at https://publons.com/publon/10.1002/jnr.24754.

## Supporting information

FIGURE S1 HCN1 and HCN3 specificity controls. Specificity controls were performed by incubating the antibody with the immunogenic peptide (control peptide, cp). HCN1 staining in the neurons (a, b) and organ of Corti (c, d) without (a, c) and with cp preincubation (b, d). e, Positive control tissue staining showing HCN1 in basket cells pinceaux in the cerebellum. For HCN3, different immunostaining detection techniques were applied to visualize low levels of HCN3 content. Cortical neurons in C57Bl/6N brain sections served as positive control tissue. Immunoreactivity was confirmed with DAB (f) and BCIP/NBT (g) stainings. Cp preincubation for HCN3 was screened with Purkinje cells in the cerebellum using DAB (h,j) and BCIP/NBT (j,k) visualization of immunostaining. HCN3 staining without (h, i) and with cp (j, k). Comparison of three methods of staining was done to assure specificity of antibody binding for different sensitivities of detection. DAB (l, o), AEC (m, p), and BCIP/NBT (n, q) visualization was assessed without (l, m, n) and with (o, p, q) cp preincubation of the HCN3 antibody. B6, C57Bl/6N; CBA, CBA/J; cp, control peptide antigen; P, postnatal day

Figure S2 HCN2 and HCN4 specificity controls. The specificity of HCN2 and HCN4 antibodies was tested using the control peptide (cp) antigen. No unspecific staining was observed for HCN2 in mouse (a, b) and human spiral ganglion neurons (SGNs, c, d). a, c, Control sections stained with HCN2. b, d, HCN2 staining preincubated with the cp antigen. For HCN4, different ages were used to test the specificity of the staining in mouse SGNs (e–j). e–h, CBA/J SGNs at P2 (e, f) and P16 (g, h) and in C57Bl/6N at P44 (i, j), without (e, g, i) and with (f, h, j) control peptide preincubation of the antibody. No unspecific staining was found at the organ of Corti for HCN2 (k‐n) and HCN4 (o–t). k,l, HCN2 staining of the organ of Corti of P44 C57Bl/6N mouse (k, l) and human (m, n), without (k, m) and with (l, n) control peptide preincubation. HCN4 staining at the sensory epithelia was performed in P2 (o,p) and P16 (q, r) CBA/J and P44 C57Bl/6N (s, t) mice without (o, q, s) and with (p, r, t) control peptide preincubation of the primary antibody. B6, C57Bl/6N; CBA, CBA/J; cp, control peptide antigen; P, postnatal day

Transparent Peer Review Report

Transparent Science Questionnaire for Authors

## Data Availability

All data are accessible upon request. Human data are restricted to privacy policies so information is limited as all the subjects were anonymized.
