# Supplementary figures and images for: HCN channels in the mammalian cochlea: Expression pattern, subcellular location, and age‐dependent changes
Source: J Neurosci Res. 2020 Nov 12;99(2):699–728. doi: 10.1002/jnr.24754 (PMC7839784; doi:10.1002/jnr.24754)

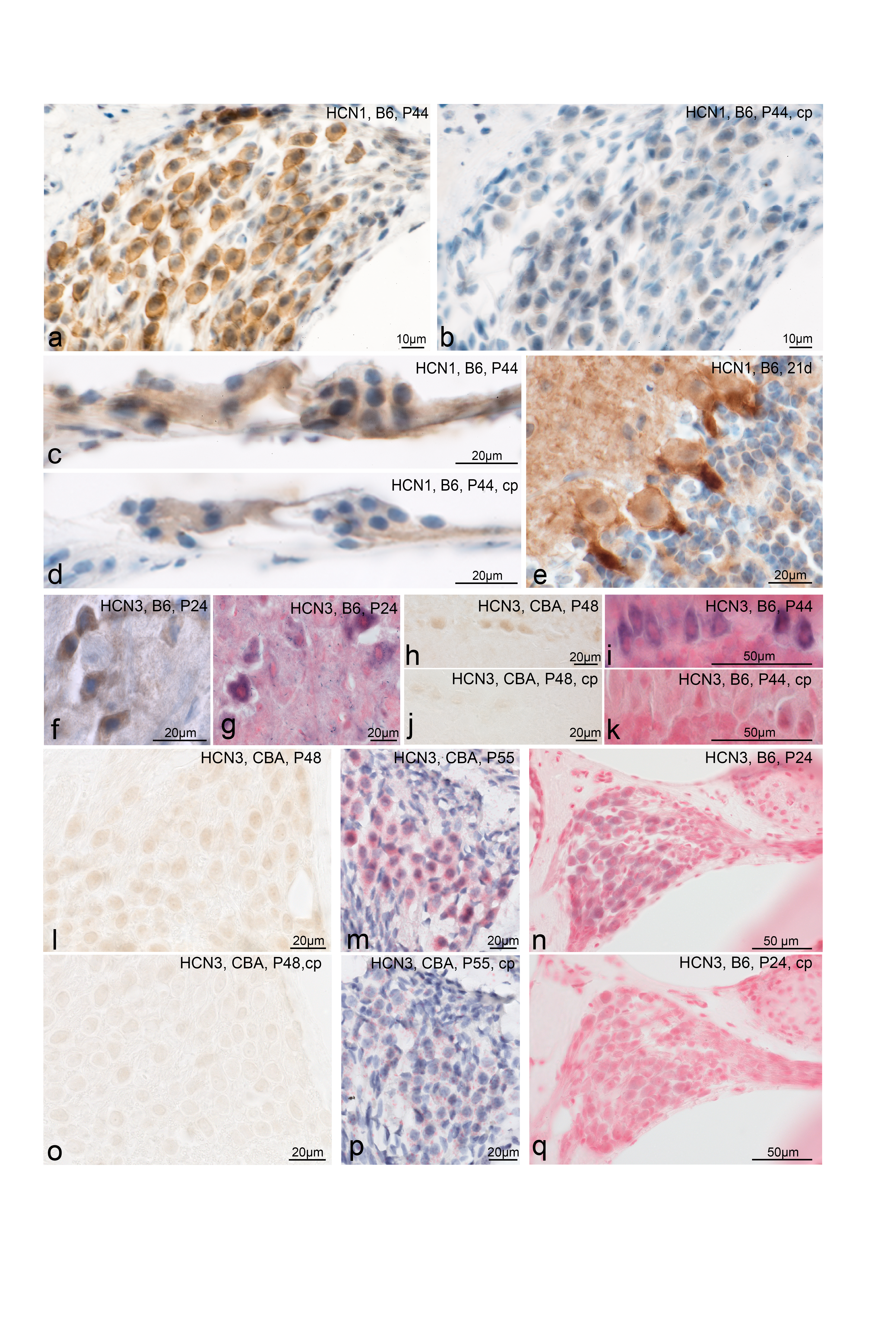

Supplement: Supplementary file 1 — FIGURE S1 HCN1 and HCN3 specificity controls. Specificity controls were performed by incubating the antibody with the immunogenic peptide (control peptide, cp). HCN1 staining in the neurons (a, b) and organ of Corti (c, d) without (a, c) and with cp preincubation (b, d). e, Positive control tissue staining showing HCN1 in basket cells pinceaux in the cerebellum. For HCN3, different immunostaining detection techniques were applied to visualize low levels of HCN3 content. Cortical neurons in C57Bl/6N brain sections served as positive control tissue. Immunoreactivity was confirmed with DAB (f) and BCIP/NBT (g) stainings. Cp preincubation for HCN3 was screened with Purkinje cells in the cerebellum using DAB (h,j) and BCIP/NBT (j,k) visualization of immunostaining. HCN3 staining without (h, i) and with cp (j, k). Comparison of three methods of staining was done to assure specificity of antibody binding for different sensitivities of detection. DAB (l, o), AEC (m, p), and BCIP/NBT (n, q) visualization was assessed without (l, m, n) and with (o, p, q) cp preincubation of the HCN3 antibody. B6, C57Bl/6N; CBA, CBA/J; cp, control peptide antigen; P, postnatal day [file JNR-99-699-s001.tif]

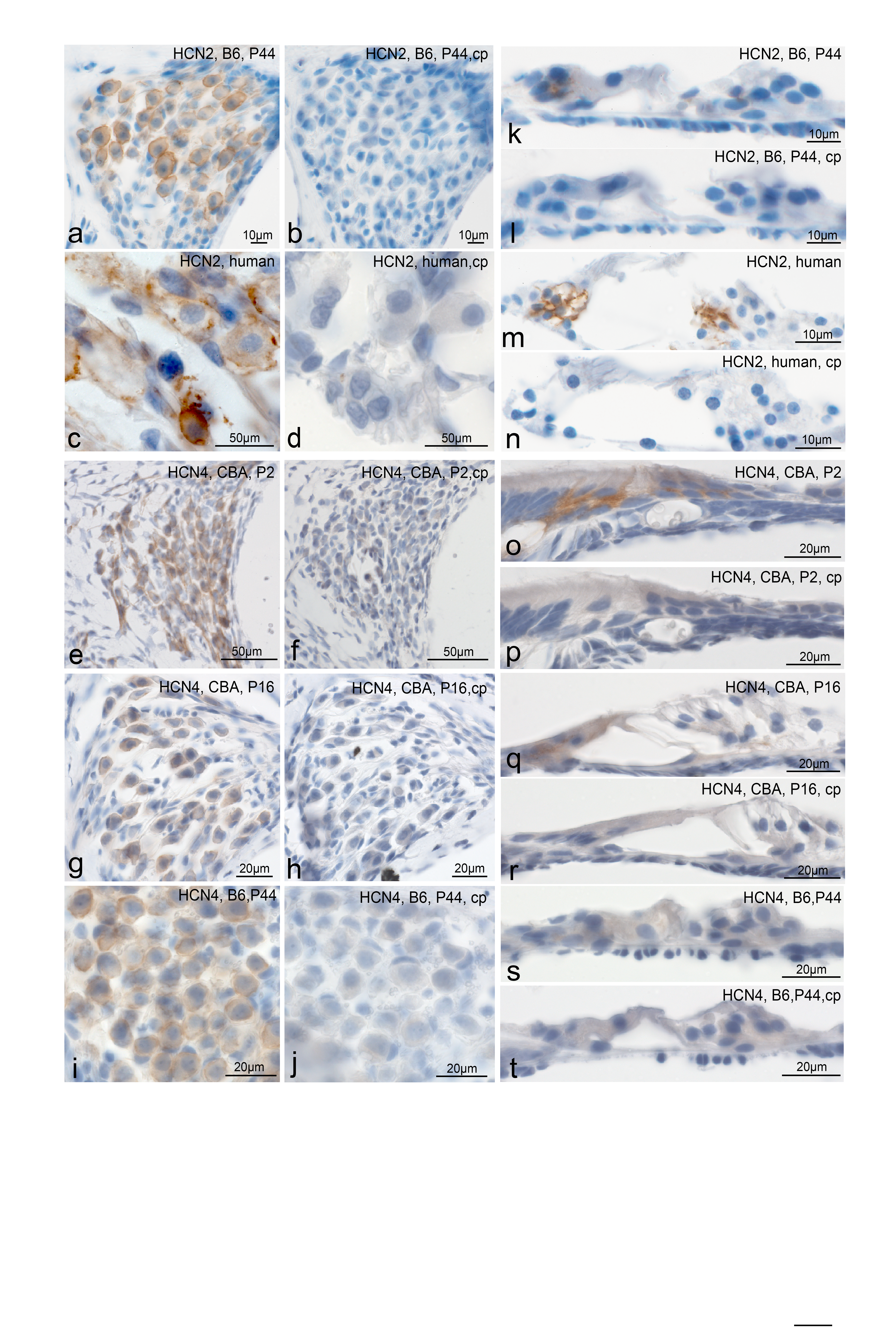

Supplement: Supplementary file 2 — Figure S2 HCN2 and HCN4 specificity controls. The specificity of HCN2 and HCN4 antibodies was tested using the control peptide (cp) antigen. No unspecific staining was observed for HCN2 in mouse (a, b) and human spiral ganglion neurons (SGNs, c, d). a, c, Control sections stained with HCN2. b, d, HCN2 staining preincubated with the cp antigen. For HCN4, different ages were used to test the specificity of the staining in mouse SGNs (e–j). e–h, CBA/J SGNs at P2 (e, f) and P16 (g, h) and in C57Bl/6N at P44 (i, j), without (e, g, i) and with (f, h, j) control peptide preincubation of the antibody. No unspecific staining was found at the organ of Corti for HCN2 (k‐n) and HCN4 (o–t). k,l, HCN2 staining of the organ of Corti of P44 C57Bl/6N mouse (k, l) and human (m, n), without (k, m) and with (l, n) control peptide preincubation. HCN4 staining at the sensory epithelia was performed in P2 (o,p) and P16 (q, r) CBA/J and P44 C57Bl/6N (s, t) mice without (o, q, s) and with (p, r, t) control peptide preincubation of the primary antibody. B6, C57Bl/6N; CBA, CBA/J; cp, control peptide antigen; P, postnatal day [file JNR-99-699-s002.tif]
