# Supplementary material for: HCN channels in the mammalian cochlea: Expression pattern, subcellular location, and age‐dependent changes
Source: J Neurosci Res. 2020 Nov 12;99(2):699–728. doi: 10.1002/jnr.24754 (PMC7839784; doi:10.1002/jnr.24754)
Supplement: Supplementary file 3 — Transparent Peer Review Report [file JNR-99-699-s003.docx]

**HCN Channels in the Mammalian Cochlea: Expression Pattern, Subcellular Location and Age Dependent Changes**

Glueckert, Rudolf (contact); Luque, Maria; schrott-fischer, Annelies; Dudas, Jozsef;

Pechriggl, Elisabeth; Brenner, Erich; Rask-Andersen, Helge; Liu, Wei

_____________________________________________________________________________________

| Review timeline: | Submission date: 15-Jan-2020  Editorial Decision: Major Modification (13-Mar-2020)  Revision Received: 22-Jun-2020  Editorial Decision: Minor Modification (09-Aug-2020)  Revision Received: 11-Sep-2020  Editorial Decision: Accept with Minor Edits (30-Sep-2020)  Revision Received: 08-Oct-2020  Editorial Decision: Accept with Minor Edits (12-Oct-2020)  Revision Received: 20-Oct-2020  Accepted: (ACC) (22-Oct-2020) |  |
| --- | --- | --- |
|  |  |  |

Editor:	Nolan, Lisa

Reviewer 1: Lv, Ping

Reviewer 2: Davis, Robin

Reviewer 3: Glowatzki, Elizabeth

_____________________________________________________________________________________

**1st Editorial Decision	13-Mar-2020**	____________________________________________________________________________________

Dear Dr Glueckert:

Thank you for submitting your manuscript to the Journal of Neuroscience Research. We've now received the reviewer feedback and have appended those reviews below. As you will see, the reviewers find the question addressed to be of potential interest. Yet, they do not find the manuscript suitable for publication in its current form.

If you feel that you can adequately address the concerns of the reviewers, you may revise and resubmit your paper within 60 days. It will require further review. Please explain in your cover letter how you have changed the present version. If you require longer than 60 days to make the revisions, please contact Dr Cristina Ghiani (cghiani@mednet.ucla.edu). You can submit your revised manuscript directly by clicking on the following link: *** PLEASE NOTE: This is a two-step process. After clicking on the link, you will be directed to a webpage to confirm. ***

https://mc.manuscriptcentral.com/jnr?URL_MASK=d7275637071648aea895c0e27006b69f

Thank you again for your submission to the Journal of Neuroscience Research; we look forward to reading your revised manuscript.

Best Wishes,

Dr Lisa Nolan

Associate Editor, Journal of Neuroscience Research

Dr Cristina Ghiani

Co-Editor-in-Chief, Journal of Neuroscience Research

Associate Editor: Nolan, Lisa

Comments to the Author:

This is a really interesting study, I read the manuscript with great interest. At present the spatiotemporal expression pattern of the HCN channel components in the cochlea is not clear. Given the role of these channels in auditory sensitivity, I strongly encourage the authors to consider the effect of sex as a biological variable (SABV) on the tonotopic expression pattern of these channels in the cochlea. The age-related loss of hearing in humans is modulated by SABV. Women have a tendency for a greater hearing sensitivity than men at the high-frequencies throughout the lifespan. In comparison, men have a tendency for a greater hearing sensitivity in low-frequencies compared to women. Therefore, more information on whether comparisons are sex-matched would be helpful – where this information is available. This could either be added to the figures/legends or discussed/acknowledged in the manuscript. One should also include somewhere if the methodologies employed will detect all isoforms of the various channel components.

At present the key messages of the manuscript are lost within the amount text. The text needs to be cut considerably – the manuscript is far too long and the discussion beyond the scope of the data. Please also note here the reviewer comments on readability. The methods section can be cut in half by citing standard methodologies. As also suggested by one of the reviewers, the authors should consider using parts of their interesting discussion for a mini-review on this topic.

The reviewers have also raised several concerns and these need to be addressed.Therefore, at present I cannot accept the manuscript for publication in JNR in its current form.

Additional points

Page 5, line 47. The authors should acknowledge that the frequencies used for the ABR recordings in the C57’s are lower than the frequencies used by Henry et al., (2002, 2004) when sex-differences in hearing thresholds were observed in this mouse strain.

Additional Editorial comments:

Please remove the list of abbreviations, this is not a format accepted by JNR, abbreviations must be provided and spelled out the first time they are used in both the abstract and in the main text.

In the figure legends, where appropriate please add the number of animals/group and the statistical test used.

Please provide the total number of animals/sex and strain used in this study.

GRAPHICAL ABSTRACT

Please upload a graphical abstract, which we are asking of all authors submitting original research articles. This is intended to provide readers with a visual representation of the conclusions and an additional way to access the contents and appreciate the main message of the work. What we require is a .tif image file and a .doc text file containing an abbreviated abstract. For the image, labels, although useful, must be kept to a minimum and the image should be 400 x 300, 300 x 400, or 400 x 400 pixels square and at a resolution of 72 dpi. This can be one of the figures from your article, or something slightly different, as long as it represents your study. Instructions for this can be found in our author guidelines online at http://onlinelibrary.wiley.com/journal/10.1002/(ISSN)1097-4547/homepage/ForAuthors.html

Please include a table where, in accord with JNR requirements, you provide additional information on each of the antibodies that you use. This must include the source (not just the manufacturer and catalog or lot number, but the species it was raised in, and the EXACT structure of the immunizing antigen - including the amino acid sequence for peptide antigens); characterization (e.g., what does it recognize on immunoblots); and appropriate controls (i.e. the effect of blocking peptides for immunohistotochemical localization). The RRID can be included here as well and removed from the methods.

SEX DIFFERENCES

The National Institutes of Health now mandates the inclusion of sex as a biological variable. To conform with this mandate, the Journal of Neuroscience Research has established new policy (please see our editorial: http://onlinelibrary.wiley.com/doi/10.1002/jnr.23979/full) requiring all authors to ensure proper consideration of sex as a biological variable. Please ensure that: 1) Any paper utilizing subjects of one sex state the sex of the sample in the title and abstract; 2) The number of samples/subjects of each sex used in the research must be clearly stated in the methods section; 3) The inability for any reason to study sex differences where they may exist should be discussed as a study limitation. We are also encouraging authors to report exploratory analyses of potential sex differences in studies not explicitly designed to address them

Reviewer: 1

Comments to the Author

This study by Rudolf, et.al is exploring the HCN channels expression pattern, subcellular location and age dependent changes in the mammalian cochlea. The authors compared the HCN expression in different mammalian species including mice (CBA, C57, and Ly5.1), guinea pigs, cats and human. The results showed that HCN channel subunits distributed unevenly among mammalian auditory neurons, and characteristic changes of expression levels were observed during postnatal development and ageing.

Although the authors present data that may be of interest and importance for hearing function, the manuscript was not adequately described to evaluate the role of HCN channel in hearing, and it need to be shortened to show a clear and concise storySince the data are rather preliminary and describable, the study needs more careful analysis and presentation of the data. Also, the author needs to explain the following question.

Major points

1 Please rewrite the manuscript in simple and clear sentences, including introduction, method, results, and discussion. Please shorten the manuscript, and make it readable.

2 I would remove the paragraph 2 (line 16-line38) in the result section to the discussion.

3 Please show the images of HCN expression along SGNs from apex to base in each mice strain, guinea pigs, cats and if possible human cochlea. Please also compare them to determine if there is any gradient of HCN expression.

4 Clarify if the HCN1 channel expresses in SGNs of guinea pig (figure 1). Please show a clear image to replace them in figure 1j.

5 In figure 2, the HCN2 and the peripherin double staining images in cat should be shown, otherwise, you can’t say HCN2 presence of type II SGNs in cat.

6 Whether HCN3 presents in OHC of mice should be stated in results.

7 Figure 10 should state the statistical test and the level of significance. Please double check that the legend age 15-19 in “C” should be 16-19.

8 The C57BL/6 strain as a model of age-related hearing loss, it displays the hearing loss by 12-15 month (Someya, et. al, 2009). Here, the onset of hearing loss is around 5-8 month, however it did not show any age-dependent hearing threshold changes from 5-8 mon to 16-19mon group. Please explain it (figure 10).

9 Please replace the figure 9j with a high-quality image.

Reviewer: 2

Comments to the Author

Summary

This is a potentially interesting paper presenting a comprehensive examination of HCN subunit

distribution patterns in the cochlea. Although the data could be quite nice if the antibodies are indeed

specific to the HCN1‐4 subunits, the paper is overly speculative, rife with overinterpretations, and, in

some cases, appears to misrepresent some of the data.

Major issues

1. There are many grammatical errors throughout the manuscript.

2. Significance: The roles of HCN channels outlined in the significance (i.e. spontaneous activity,

spiking acuity, tuning spiking frequency and control of summation effects) are not shown

directly in the study.

3. Abstract/Significance. The authors merely speculate that HCN location has something to do

with ephaptic coupling; nor has this mechanism been shown in the spiral ganglion. Therefore,

this finding should not be highlighted in the abstract or significance sections.

4. The first paragraph of the results section belongs either in the introduction or discussion

sections.

5. Comparisons in immunolabeling should not be made unless identical conditions have been

established.

6. The antibody controls specifically tested labeling after pre‐adsorption with the immunogenic

antigen peptide according to the manufacturer’s protocol. This is important, but the authors did

not take the next critical step to evaluate the specificity of the antibodies for each HCN subunit.

7. Figure 1. The localization of HCN1 to the AISC and not the AISP is an interesting finding but is

not convincingly presented.

8. Could the bright labeling in Fig. 1b be a staining artifact? A co‐label with a neuron‐specific

antibody would have resolved this issue.

9. How is HCN2 labeling distinguished between neuronal membrane labeling and myelin and/or

Schwann cell membrane labeling?

10. The authors should justify their correlations between electrophysiological assessments and HCN

channel expression given the likely scenario that more than just HCN channels change during

these time periods.

11. The potential role of HCN channels in ephaptic coupling is an interesting point for discussion,

placing this point as a finding in the abstract and significance is a bit of a stretch.

12. The discussion is overly long and highly speculative.

Reviewer: 3

Comments to the Author

This study examines the expression and localization of HCN channel subunits 1-4 in the spiral ganglion neurons and the organ of Corti across different ages in six animal models, with carefully performed anatomical studies, both regarding HCN channel labeling as well as statistical analysis of the data. This is important work because it is highly likely that HCN channels play an important role in auditory nerve fiber activity, and other neuronal elements in the inner ear. Secondly, there is controversy about HCN channel expression, localization and function in the auditory periphery. The authors report a plethora of findings that differ across ages and animal models in a truly staggering amount of work. The authors report the expression of antibody labeled subunits of the HCN channel in the type I and type II SGN somata, peripheral and central axonal initial segments, type I and type II afferent fibers and in MOC efferent fibers and hair cells. This is a tremendous effort to catalog HCN subunit expression in a generally well-written paper. The main results are 1) specific HCN subunit expression in the immature cochlea; 2) HCN2 and HCN4 channel expression is prominent in the mature cochlea; HCN3 is found in OHCs 4) HCN channel expression at different ages was not strongly correlated in mice with ABR thresholds. It is important to see that HCN channel expression varies between species.

Major concerns:

• It is very hard to read the manuscript and to comprehend what the significant message from this study is. The detail and exactness of the results is appreciated, however, the main message gets lost in the detail. Multiple approaches could be taken to improve the readability of the manuscript.

Detailed comments;

a) better state the specific goal of the study at the beginning; state the main results in abstract, which is not the case right now. The abstract trails of on details of channel physiology that do not represent the results presented here in the study

b) better annotate the figures:

As an example: in Fig. 10a,c the strain could be written into the figure, so one can read the meaning of the figure without the legend. Actually the legend is incorrect, and states that a,b show the different strains, but it’s a,c and b,d. Instead of threshold it could say eABR threshold, and instead of amplitude it could say Wave I amplitude. The supplement of Fig. 11 needs to be part of the manuscript, as the statistical analysis is key.

Table 1: One definitely expects a table to review the data, however the table as presented is still hard to read. Maybe for HCN 1,2,3,4, there need to be subcategories by species, so there is a + for every species if the expression is found, so we see clear boxes with either a “+” a “–“ or a blank. This will give the results that occur generally some weight visually. The second half of table 1 seems out of place in this study, as no physiology was presented, and the entries seem out of context and somehow random. This should be better just reviewed in a paragraph in discussion.

c) there are too many grammatical errors and typos in the manuscript

d) Detailed information and important results are mixed in a way that make it hard to get the main message. It would be helpful to have short summaries of the main results (not repeat of all results) at the end of the individual chapters. This is already implemented in some chapters

e)The discussion reads like a separate review article on HCN channel function in general and in the inner ear. It is comprehensive and insightful, but also highly speculative and trails off quite far from the data presented here and the discussion of the data in the context of the literature. Some readers may be grateful for this excellent summary of the state of the field, however, it is hard to find out again, what were the important results of the paper and what needs to be critically discussed regarding the data.

This may be more of a personal choice on how to embed one’s results into the field, however, these reviewers felt they would have been better served with a much shorter but concrete discussion that points to the main results. A critical assessment of the labeling, imaging and analysis methods used is provided throughout the paper, but could have been summarized in a concise way in the discussion. In our opinion, most parts of discussion should be separated from the paper and published as a review article. The insight, that the understanding of HCN channel function will help with the design of cochlear implants is appreciated and shared by the reviewers.

• The authors include functional audiometric data at different time points to correlate audiometric function with the expression of HCN channels. This part is weak, as it is just a descriptive comparison. As there are numerous other factors that contribute to an increase in audiometric threshold and a decrease in response amplitude with increasing age, so such a connection is tenuous at best.

• The ion channel co-expression analysis is unclear and needs to be better explained

• Were the animal species/strains raised under the same conditions? Different noise exposure in the animal facility may possibly affect the gene expression pattern in the inner ear. It might be helpful to allow comparison with other groups to measure the sound exposure and add this to methods, or at least to state if the different mouse models/ animal models were raised under the same conditions. This could account for expression differences besides species/strain differences.

• Page 17; Line 48: the statement that wave I amplitude indicates the number of SGNs firing is not completely correct. It would also indicate synchrony of firing, and the same number could provide different amplitudes with different levels of synchronous firing. It would also be helpful to have statistical test result annotated in the figure, so one can see which decline in amplitude is statistically significant.

• For the comparison of HCN channel expression and ABR threshold/wave I, plots should be presented with the same x axis (age), so one can visually compare trends. It is difficult to compare Figs. 10 and 11.

• Pge 18, HCN quantification. Although stated in methods, here the main points of the quantification method should be shortly introduced. It needs to be stated, which regions were quantified (SGN somata?). How were the data normalized between ages? And species? The suppl/ figure 1 needs to be explained and introduced in the text (it should not be suppl., but part of the main manuscript). What was tested here against what? It is confusing because one expects to see a test HCN expression versus ABR data.

*********IMPORTANT: Instructions and checklists follow*********

When finalized, please upload your complete revised manuscript onto our website, preferably as a word document. We require a clean version of the manuscript, in which the change-tracking feature is turned off and one using the track changes function to highlight the changes you make to the manuscript. Figures must be uploaded separately in .tif or eps format. Please review our submission checklist, which can be found in our author guidelines and also be sure to fill out the Transparent Science Questionnaire attached to this email.

To assist in our transparent peer review process, please upload the decision letter and response to reviewers using the attached template document. The first section should include the decision letter in full. In the Authors Response section, please provide the reviewer comments and your response in red.

JNR offers Open Science badges to qualifying authors. For more information please see the “Open Science initiatives” section of our author guidelines. If you would like to apply for one or more of the badges, please complete the included disclosure form and upload it as Supplemental Material Not for Review when submitting your final manuscript files.

To submit your revised manuscript:

*You have two ways to submit your revision. Either click on the link: *** PLEASE NOTE: This is a two-step process. After clicking on the link, you will be directed to a webpage to confirm. ***

https://mc.manuscriptcentral.com/jnr?URL_MASK=ad3b366a0101445da028dcbff890e88c or Log on to https://mc.manuscriptcentral.com/jnr using your case-sensitive User ID ((Person not available)).

For security purposes your password is not listed in this email. If you are unsure of your password you may click the link to set a new password.

(Person not available)

*Enter the Author Center, where you will find your manuscript title listed under “Manuscripts with Decisions”

* Under “Actions,” click on “Create a Revision”. Your manuscript number has been appended to denote a revision.

*Follow the prompts and replace existing files with revised ones, as necessary.

If you encounter any troubles in submitting your revised manuscript, please contact Dr Cristina Ghiani (cghiani@mednet.ucla.edu) or click on Get Help Now at the top right of any ScholarOne Manuscripts screen.

_____________________________________________________________________________________

**Authors’ Response	22-Jun-2020**

Response to Decision Letter:

Dear Prof. Nolan,

First of all, we are grateful to get the chance to revise our manuscript. We realized that the big amount of data was presented in a way that is hard to follow for the “non-authors”. We shortened and changed the manuscript fundamentally, addressed yours’s and the reviewer’s concerns and critics and hope we could considerably improve the text, figures and tables.

We add a highlighted version like requested instead of a “tracked changes” version of the document and hope it can be valuable for the revision process. In our answers to the reviewers we point out main changes and address questions and critics of the editor and reviewer point by point.

As some general critics arouse about our staining specificity, we added several supplemental figures of controls and referenced this also in the text. Whether these figures shall be part of this manuscript -in case it will be published in this Journal- or serve only as a supportive material for this review we leave over to the editor. The authors are fine with either case.

One more problem arose: In some of the checklists that were sent within this revision process a word limit for the introduction (<600 words) and discussion (<1600 word) was listed, in other checklists we received within this review process there was no such limit mentioned that conforms with the guidelines for research articles. This was very confusing for us. Since we cannot fulfil these requirements with the “new” word limitations, I leave the decision to the editor whether to continue with the review process of our manuscript.

Kind regards on behalf of the authors

**Reviewer 1**

Dear Reviewer 1,

First of all, we are grateful for your comments and critics of our manuscript and the time and effort you took to go through our very complex manuscript. We realized that the big amount of data was presented in a way that is hard to follow for the “non-authors”. We shortened and changed the manuscript fundamentally, addressed your concerns and critics and hope we could considerably improve the text, figures and tables.

We add a “tracked changes” version of the document and hope it can be valuable for the revision process. In our answers to the reviewer we point out main changes and address questions and critics point by point. As some general critics arouse about our staining specificity, we added several supplemental figures of immunostaining controls and referenced this also in the text.

Kind regards on behalf of the authors

Rudolf Glueckert & Maria Luque

**Reviewer: 1**

**Comments to the Author**

**This study by Rudolf, et.al is exploring the HCN channels expression pattern, subcellular location and age dependent changes in the mammalian cochlea. The authors compared the HCN expression in different mammalian species including mice (CBA, C57, and Ly5.1), guinea pigs, cats and human. The results showed that HCN channel subunits distributed unevenly among mammalian auditory neurons, and characteristic changes of expression levels were observed during postnatal development and ageing.**

**Although the authors present data that may be of interest and importance for hearing function, the manuscript was not adequately described to evaluate the role of HCN channel in hearing, and it need to be shortened to show a clear and concise story. Since the data are rather preliminary and describable, the study needs more careful analysis and presentation of the data. Also, the author needs to explain the following question.**

**Major points**

**1 Please rewrite the manuscript in simple and clear sentences, including introduction, method, results, and discussion. Please shorten the manuscript, and make it readable.**

The authors cut the text considerably! All sections have been shortened and several details on functional implication removed. We are aware that the manuscript is still big. As we did not want to divide this manuscript into a developmental, ageing and immunolocalization paper, we left everything together to provide a comprehensive summary of mammalian HCN channel location and added a more limited discussion on functional implications.

We added 3 hypotheses in the beginning of the manuscript to clearly point out the aim of this paper (page 5, lines 14-18):

*A detailed map of HCN location and expression profiles shall give us answers on:*

*I.)	whether we find differences in HCN expression among mammalian species,*

*II.)	if we can correlate the HCN expression pattern with hearing performance during ageing in mouse strains with different types of age-related hearing loss (ARHL)*

*III.)	track the onset and differential expression of HCN channels during mouse developmental stages to correlate with known data on auditory function.*

**2 I would remove the paragraph 2 (line 16-line38) in the result section to the discussion.**

The second paragraph of the original result section was rearranged and moved to the introduction (page 5, lines 7-12 and lines 21-26 and page 6, lines 1-5).

**3 Please show the images of HCN expression along SGNs from apex to base in each mice strain, guinea pigs, cats and if possible human cochlea. Please also compare them to determine if there is any gradient of HCN expression.**

A visual determination of any tonotopic gradient with a membrane staining is not easy to accomplish-we tried! But our ability to do that visually on some sections were exposed to be incorrect when we compared with user independent segmentation and quantitative evaluation. We re-checked the method with semiautomatic segmentation in ImageJ and realized that in the case with heterogenous membrane staining the computer does a better job than even decades of histologic experience can do. It is not only the neuron clusters with highest immunoreactivity that biased our visual observations, but mainly this heavily and faintly stained cells next to each other were patterns of staining that fooled our visual perception. The authors agree with the reviewer that with more evenly distributed immunostainings this would be much easier to assess in a rapid way. So, that is the reason why we quantified the distribution in two different mouse strains -C57BL and CBA-at single neuron level with image analysis tools.

To get reliable data on any tonotopic gradient a considerable number of individuals with documented sex, age, weight etc. and enough sections needed to be immunostained and quantified for a statistical analysis. These prerequisites were available for our mouse inbred strains but not for cat, guinea pig and human where we aimed a qualitative but not quantitative approach. Any description on gradients in species with limited amount of histologic material or presentation of images from limited number of individuals was therefore not reliable enough for us. What we could offer and added in the document is a new graphical representation of statistical p-value of HCN channels expression levels in the SGNs membranes in different cochlear turns for each age group.

That figure together with our revised figure 10,

provides the possibility to see statistically significant gradients in each turn of each age group in C57Bl/6N as well as CBA/J mice. The main result of this work turned out that we cannot find ONE gradient in either of the two examined mouse strains, moreover the tonotopical gradient is dependent on the mouse strain and the age and can even revert along the lifetime of a certain mouse strain! This shall exemplify that a simple description of HCN gradients with the use of a couple of images from other species is not reliable.

We thank the reviewer for this input! We address this in the text of the manuscript and implement more information and discussion of HCN gradients along the tonotopic axis.

We did not show representative images of the gradients in our examined mouse strains of each turn and each HCN channel subunit since we think a quantitative representation showing also the variability within a neuron subpopulation is more robust and informative. So we keep with the overview images that we presented previously and hope we could address the GRADIENT topic adequately.

In section 3.4.2 HCN Quantification we added information on gradients and in the discussion, we changed the title of section 4.2 to **4.2 HCN tonotopical gradients and co-expression in neurons.**

*HCN channels are not uniformly dispersed along the tonotopic axis. Gradients depend on HCN subtype, mouse strain and even age. In most situations, apical neurons displayed higher expression for HCN1 but also high levels of HCN2 and HCN4 channels, contrary to earlier reports that assigned HCN1 to be more expressed in basal neurons (Liu et al., 2014). The authors described a more positive V_½_ for I_h_ in the apex. That could be explained by high levels of HCN1.*

In the legend to figures we added comments on tonotopical gradients (**highlighted blue**):

***Figure 1. HCN1 expression in the adult mammalian cochlea.***

*HCN1 channel subunits are highly expressed in neurons with intense somatic- and perisomatic staining.* ***a****, CBA/J SGNs staining of neuron somata with DAB immunohistochemistry. There is no visible gradient along SGNs of the middle (SGm) and basal (SGb) turns*

***Figure 4. HCN3 expression in the adult mammalian cochlea.***

*HCN3 is weakly expressed in SGNs of mice (****a-d****), guinea pig (****i****), cat (****k****) and human (****o****). In guinea pig and cat HCN3 was detected at the level of the hair cells (****e-f*** *and* ***j****).* ***a****, midmodiolar view of C57Bl/6N without hematoxylin staining. SGNs present lightly staining. A tonotopic gradient is observed from apex to base.*

***Figure 5. HCN4 expression in the adult mammalian cochlea.***

***a,*** *midmodiolar view of section from a C57Bl/6N mouse* *exemplified the tonotopical gradient apex to base.*

***Figure 7. HCN1 and HCN3 postnatal expression until the onset of hearing.***

*HCN1 and HCN3 were only expressed at the neuronal level. Differences in the expression of these channels were evaluated during maturation from the first postnatal day until the onset of hearing at 16 days in C57Bl/6N.* ***a-h,*** *HCN1 expression in 1, 7, 9 and 16 days old mice.* ***a,b:*** *HCN1 in* *P1 C57Bl/6N mice* *showed an apical-basal gradient regulation (****a****). Staining in neurons is present already at the membrane and very intense at the soma (****b****).* ***c,d:*** *apical-basal gradient is seen at P7 (****c****), staining intensity decreases at the soma and HCN1 is found mainly at the membrane (****d****).* ***e,f:*** *At P9, the gradient of staining between different turns fades (****e****), with HCN1 mainly present at the neuronal membrane (****f****).* ***g,h:*** *at the onset of hearing, the apical turn presented high expression of the channel at the neuronal clusters (****g****) and overall intensity increased in the membrane and cytoplasm (****h****).*

***i-p:*** *HCN3 expression at 1, 7, 9 and 16 days old C57Bl/6N.* ***i,j:*** *at P1 no gradient for HCN3 between turns was observed (****i****), but high expression of HCN3 at the neuronal soma (****j****).* ***k-p:*** *no visible gradient was detected during postnatal development at P7 (****k****), P9 (****m****) until onset of hearing (****o****) and cytoplasmic staining decreased (P7,* ***l****; P9,* ***n****; P16,* ***p****).*

***Figure 8. HCN2 and HCN4 postnatal expression until the onset of hearing.***

*HCN2 and HCN4 were expressed at SGNs and the organ of Corti in C57Bl/6N as early as P1 and are differentially regulated during maturation until onset of hearing. a-l: HCN2 expression at 1, 7, 9 and 16 days after birth. a-c: HCN2 is highly expressed at P1 without any visible tonotopical gradient (a). Staining located at the soma (b) as well as in the organ of Corti at type I afferent fibers underneath the IHC (c). d-f, at P7 the staining decreases in neurons (d,e) as well as sensory epithelium (f). g-i, neurons present similar staining to P7 (g, h) but the intensity underneath the inner hair cell increased and faint staining underneath the OHCs appears (i). j-l, at the onset of hearing, SGNs presented more intense staining at the perisomatic neuron membranes compared to P9 and P7 (j, k). The functional organ of Corti expressed HCN2 in type I afferents as well as in the efferent fibers underneath the OHCs (l). m-x: HCN4 expression in 1, 7, 9 and 16 days old C57Bl/6N. m-o: High intensity staining of HCN4 is seen at P1 at the soma membrane of the SGNs in each turn (m-n). Prominent staining is visible underneath the IHC and OHCs corresponding to afferent fibers (o). p-r: at P7 a tonotopical gradient is detected from apex to base and higher expression is observed in apical clusters (p). Staining intensity decrease at the neuron membrane in single neurons (q) but prominent staining is still present in the afferent fibers at the organ of Corti (r). s-u, similar staining pattern is present in P9 neurons (s, t) and organ of Corti (u). v-w, after the onset of hearing, the tonotopical gradient fades with similar levels of staining in all turns (v), while overall staining in single neurons remain constant (w). No visible specific staining is detectable at the organ of Corti (x).*

**4 Clarify if the HCN1 channel expresses in SGNs of guinea pig (figure 1). Please show a clear image to replace them in figure 1j.**

We tested several antibodies raised against HCN1. The only antibody that gave a membrane staining with our techniques and thereby was able to immunolocate HCN1 on its functional site in a membrane was an antibody produced in guinea pig (RRID:AB_2756625, table 1). The known problems with working on guinea pig sections with a guinea pig antibody resulted in an enormous background that we were not able to suppress enough. For this reason, we have not included guinea pig images in HCN1 localization figure (Fig. 1) and cited the excellent study of previous work by Bakondi (Bakondi et al., 2009).

Among other we tested a rabbit anti-HCN1 antibody (RRID: AB_2039900, table 1). In our hands there was no specific staining with this antibody. Our control slides pre-incubated with the available control peptide antigen resulted in equal staining than without epitope saturation. We added supplemental files where we present also these results in mice (Supplementary Fig.1).

**5 In figure 2, the HCN2 and the peripherin double staining images in cat should be shown, otherwise, you can’t say HCN2 presence of type II SGNs in cat.**

Type II cells are relatively easy to identify in cat and human with morphological criteria, even at light microscopic level. The founder of the Inner Ear Innsbruck Lab was Heinrich Spoendlin who worked a lot on the importance of type II neurons and gave us his expertise to differentiate them. Safe identification is much more difficult in mice at light microscopic level that is why we always included an anti-peripherin immunostaining. Anyhow, **we added an anti-peripherin/HCN2 double staining with cat and add it to the revised figure 2 (figure 2k-m).**

HCN1, HCN2, HCN3 and HCN4 were present in cat in peripherin positive type II neurons. In figure 2, HCN2 staining in cat image was replaced by the image with double staining HCN2 and peripherin staining. For the other HCN channels we added an image **for HCN1 (new figure 1l-n) figure 4 (p-r for HCN3) and figure 5 (HCN4-figure h-j).** That should provide convincing data that cat as well as human type II cells show a much greater variability than in mouse.

**6 Whether HCN3 presents in OHC of mice should be stated in results**.

HCN3 was not present in OHCs in mice. Even with highly sensitive immunohistochemical techniques there was not specific signal at this location. Results for every specie and localization are **now clearer summarized in table 3.** This statement was also included in the text in:

Result section (Page 16, lines 7-8).

*We could not find a clear membrane staining of HCN3 in any strain of mice studied or human OHCs.*

Discussion section (page 34, lines 20-22)

*We could not detect HCN3 in C57Bl/6N, Ly5.1 and CBA/J inbred mouse strains, so the animal model to study HCN involvement in OHC function may be crucial. Expression levels in inbred mice may have dropped below detection level and in human material, we were limited by good preservation of hair cell membranes for reliable data.*

**7 Figure 10 should state the statistical test and the level of significance. Please double check that the legend age 15-19 in “C” should be 16-19.**

Labelling in figure 10 (now **new figure 9**) is corrected now. Figure 9c states now 16-19 in the legend. The y-axis was also changed to: eABR threshold (dB) vs frequency (KHz) in figures a and c. Figures b and d show wave I amplitude (V) vs age (months). In the y-axis for each figure now the strain of mice is written. Also, as a title in each graph so the figure can be easily understood without looking at the legend. In addition, we have incorporated the statistics to the figure as a summary table below each graph. As there are many variants and statistical significances, we cannot incorporate it directly into the graph and chose this kind of format. The legend from figure 9 is corrected (page 59, lines 12-21) and the statistical test applied and significances were also written.

**8 The C57BL/6 strain as a model of age-related hearing loss, it displays the hearing loss by 12-15 month (Someya, et. al, 2009). Here, the onset of hearing loss is around 5-8 month, however it did not show any age-dependent hearing threshold changes from 5-8 mon to 16-19mon group. Please explain it (figure 10).**

Reproducibility of animal work is always a big problem in science-we are absolutely aware of this fact. In previous publications scientists described the onset of hearing loss (HL) in C57BL/6 at 3-5 months with an increase in thresholds and bigger changes were found at high frequencies (Hunter and Willott, 1987; Park et al., 2010). There could be differences in HL between Someya, et. al, 2009 and our results because of the different background in this strain of mice. In our studies we are using C57Bl/6N (purchased from Charles River Germany) instead of C57Bl/6J (directly from the Jackson laboratories) used in many other studies especially from the US. We regularly refresh our strain with new individuals for breeding to avoid a genetic drift and directly purchase from Charles River Germany. (Kane et al., 2012) also described no differences in HL between C57Bl/6N and C57Bl/6J, but we cannot exclude some differences. Breeding conditions may further add a variable that is underestimated anyway.

Kane et al., 2012 described HL at 32 KHz starting already at 6 months, and for 16 KHz to be significative different at 12 months. The biggest changes in hearing thresholds appear after 5 months, afterwards thresholds are stable until 15 months when a next increase in hearing thresholds is observed. In this study thresholds significantly increased from 3 to 6 months and afterwards continued to increase but at a lower level. The reason could be a higher loss of hair cells by that age especially in the basal turn and also spiral ganglion neurons (Park et al., 2010). Maybe the biggest loss occurs at this age, followed by steady state were compensatory mechanisms are acting until older ages.

We have chosen to test our own cohort of animals and did our own eABR testing due to the high variability of eABR data in the literature. This we regarded as a prerequisite to be able to compare two mouse strains with different phenotype of hearing loss. Housing and different environmental noise can severely affect mice hearing loss apart from other nonauditory effects elsewhere described (Turner et al., 2005). In our study all the animals were exposed to the same conditions and both strains were kept at the same room in the animal facility in Innsbruck, so the environmental variable between mouse strain is exactly equal. Also, animals with considerable different weight from age matched individuals or mice with middle ear infection were immediately discarded and never evaluated or considered. The hearing deterioration of our C57Bl/6N mice is within the range of previously published data but may not absolutely conform with a certain result as the reviewer mentioned. This underline the necessity to test own animals for comparative work and regular fresh up the genotype.

**9 Please replace the figure 9j with a high-quality image**.

A new image was added to figure 9j (now **new figure 8**). Heat induced antigen retrieval sometimes leads to a detachment of bony tissue. Other tissue resides to its original site on the slide so overview section may sometimes show these detaches bone tissue.

References cited in the text:

Bakondi, G., Por, A., Kovacs, I., Szucs, G., and Rusznak, Z., 2009, Hyperpolarization-activated, cyclic nucleotide-gated, cation non-selective channel subunit expression pattern of guinea-pig spiral ganglion cells: Neuroscience, v. 158, no. 4, p. 1469-1477.

Hunter, K. P., and Willott, J. F., 1987, Aging and the auditory brainstem response in mice with severe or minimal presbycusis: Hear Res, v. 30, no. 2-3, p. 207-218.

Kane, K. L., Longo-Guess, C. M., Gagnon, L. H., Ding, D., Salvi, R. J., and Johnson, K. R., 2012, Genetic background effects on age-related hearing loss associated with Cdh23 variants in mice: Hear Res, v. 283, no. 1-2, p. 80-88.

Park, S. N., Back, S. A., Park, K. H., Kim, D. K., Park, S. Y., Oh, J. H., Park, Y. S., and Yeo, S. W., 2010, Comparison of cochlear morphology and apoptosis in mouse models of presbycusis: Clin Exp Otorhinolaryngol, v. 3, no. 3, p. 126-135.

Turner, J. G., Parrish, J. L., Hughes, L. F., Toth, L. A., and Caspary, D. M., 2005, Hearing in laboratory animals: strain differences and nonauditory effects of noise: Comp Med, v. 55, no. 1, p. 12-23.

Bakondi, G., Por, A., Kovacs, I., Szucs, G., and Rusznak, Z., 2009, Hyperpolarization-activated, cyclic nucleotide-gated, cation non-selective channel subunit expression pattern of guinea-pig spiral ganglion cells: Neuroscience, v. 158, no. 4, p. 1469-1477.

Hunter, K. P., and Willott, J. F., 1987, Aging and the auditory brainstem response in mice with severe or minimal presbycusis: Hear Res, v. 30, no. 2-3, p. 207-218.

Kane, K. L., Longo-Guess, C. M., Gagnon, L. H., Ding, D., Salvi, R. J., and Johnson, K. R., 2012, Genetic background effects on age-related hearing loss associated with Cdh23 variants in mice: Hear Res, v. 283, no. 1-2, p. 80-88.

Liu, Q., Manis, P. B., and Davis, R. L., 2014, I h and HCN channels in murine spiral ganglion neurons: tonotopic variation, local heterogeneity, and kinetic model: J Assoc Res Otolaryngol, v. 15, no. 4, p. 585-599.

Park, S. N., Back, S. A., Park, K. H., Kim, D. K., Park, S. Y., Oh, J. H., Park, Y. S., and Yeo, S. W., 2010, Comparison of cochlear morphology and apoptosis in mouse models of presbycusis: Clin Exp Otorhinolaryngol, v. 3, no. 3, p. 126-135.

Turner, J. G., Parrish, J. L., Hughes, L. F., Toth, L. A., and Caspary, D. M., 2005, Hearing in laboratory animals: strain differences and nonauditory effects of noise: Comp Med, v. 55, no. 1, p. 12-23.

**Reviewer: 2**

Dear Reviewer 2,

We wish to thank for the time and effort the reviewer spent on our manuscript. We realized that the big amount of data was presented in a way that is hard to follow for the “non-authors”. We shortened and changed the manuscript fundamentally, addressed your concerns and critics and hope we could considerably improve the text, figures and tables.

We removed many of our interpretations and moved our interpretation of functional implications to the discussion.

We add a “tracked changes” version of the document and hope it can be valuable for the revision process. In our answers to the reviewers we point out main changes and address questions and critics of the editor and reviewer point by point.

As the reviewer expressed general critics about our staining specificity, we added several supplemental figures of immunostaining controls and referenced this also in the text. Whether these figures shall be part of this manuscript -in case it will be published in this Journal- or serve only as a supportive material for this review we leave over to the editor. The authors are fine with either case.

Kind regards on behalf of the authors

Rudolf Glueckert & Maria Luque

**Comments to the Author**

**Summary**

**This is a potentially interesting paper presenting a comprehensive examination of HCN subunit distribution patterns in the cochlea. Although the data could be quite nice if the antibodies are indeed specific to the HCN1‐4 subunits, the paper is overly speculative, rife with overinterpretations, and, in some cases, appears to misrepresent some of the data.**

The authors cut the text considerably! All sections have been shortened and several details on functional implication removed. We are aware that the manuscript is still big. As we did not want to divide this manuscript into a developmental, ageing and immunolocalization paper, we left everything together to provide a comprehensive summary of mammalian HCN channel location and added a more limited discussion on functional implications.

We added 3 hypotheses in the beginning of the manuscript to clearly point out the aim of this paper (page 5, lines 12-18):

*A detailed map of HCN location and expression profiles shall give us answers on:*

*I.)	whether we find differences in HCN expression among mammalian species,*

*II.)	if we can correlate the HCN expression pattern with hearing performance during ageing in mouse strains with different types of age-related hearing loss (ARHL)*

*III.)	track the onset and differential expression of HCN channels during mouse developmental stages to correlate with known data on auditory function.*

**Major issues**

1. **There are many grammatical errors throughout the manuscript.**

We corrected many grammar and spelling errors.

**2. Significance: The roles of HCN channels outlined in the significance (i.e. spontaneous activity, spiking acuity, tuning spiking frequency and control of summation effects) are not shown directly in the study.**

The aim of this study is to corelate previous electrophysiological and functional data already published with a description of HCN localization by different immunohistochemical techniques. Therefore, it is important to outstand possible functions for HCN depending on the localization. We agree with the reviewer that the significance statement should just show our own results so it was changed as the reviewer suggested.

Page 3, lines 2-7: *Spiral ganglion neuron heterogeneity contributes to the fantastic dynamic range and timing precision of auditory perception. We provide a detailed description of subcellular HCN subunit location in a comparative analysis across mammalian species including human. Spatiotemporal expression during postnatal development and ageing suggests HCN channels to be involved as an active component. Presence in outer hair cells and neuron clusters highlights the importance. HCN channels require consideration for new electrical stimulation strategies.*

**3. Abstract/Significance. The authors merely speculate that HCN location has something to do with ephaptic coupling; nor has this mechanism been shown in the spiral ganglion. Therefore, this finding should not be highlighted in the abstract or significance sections.**

The connection between ephaptic coupling and more HCN channel expression in adjacent membranes from neurons in a cluster is removed from the abstract (tracked document page 5, line 15) and significant statement (tracked document page 4, line 5).

**4. The first paragraph of the results section belongs either in the introduction or discussion sections.**

The first paragraph of the result sections was removed. The second paragraph of the original result section was rearranged and moved to the introduction (page 5, lines 7-12 and lines 21-26 and page 6, lines 1-5).

**5. Comparisons in immunolabeling should not be made unless identical conditions have been established**.

Exactly that was always our aim and we are aware of the problems in comparing and quantify immunohistochemical staining! All the direct comparison of the staining intensities were done between sections stained with the same antibody, same lot and same conditions. With manual techniques this is absolutely impossible, that is why we implemented highly reproducible automated techniques in our lab many years ago. Our fully automated immunostaining system (Ventana® Discovery Immunostainer, Roche® Medical Systems inc. RRID:SCR_013652) allowed us to reproduce exactly the same conditions in each staining run. Also, the concentration for the primary and secondary antibody was always the same. The extraction of the ears, fixation and embedding protocols are also standard for all the mice specimens and differ from cat and human where no comparison in staining intensities were done in this study. Moreover, the animals were bred in the same room under identical conditions at the same period of time, reagents for fixing and embedding were equal and all sectioning were performed by the same person with identical equipment. Fully automated imaging was done with one microscope, all segmentation of nuclei and immunostaining was performed with an optimized algorithm in a user independent way to avoid bias. The authors regard this high grade of standardization and automatization as a strength in this work! That is why we dared to perform a comparison of genetical homogenous inbred mice that we refreshed continuously to avoid any genetic drifts.

**6. The antibody controls specifically tested labeling after pre‐adsorption with the immunogenic antigen peptide according to the manufacturer’s protocol.** **This is important, but the authors did not take the next critical step to evaluate the specificity of the antibodies for each HCN subunit.**

To test the specificity of our antibodies and the quality of the results we have made different positive and negative controls for HCN1, HCN2, HCN3 extracellular and HCN4 antibodies.

**We added 4 sets of supplemental figures: supplementary figures for HCN1 (Sup. Fig. 1), HCN2 (Sup. Fig. 2), HCN3 (Sup. Fig. 3) and HCN4 (Sup. Fig. 4) to show our most important specificity tests.**

There were several primary HCN antibodies that we bought from companies and did not fulfil our needs and were not further

*Positive control tissue:*

Brain, cerebellum and other tissues (kidney, olfactory epithelium, liver, skeletal muscle and glands) to compare stainings with the data from literature.

*Specifity and Negative controls:*

*A: Control peptide antigen preincubation:* A preincubation of the primary antibody with the antigen (peptide) that served as epitope to produce the primary antibody in at least 20x excess shall block specific binding sites. Any other binding that results in a positive immunostaining is unspecific.

*B: Control peptide antigen preincubation with centrifugation prior slide application:*

A centrifugation step for 10 minutes at 15.000g after control peptide antigen preincubation was added in those cases where there was unspecific signal after pre-incubation of the primary antibody with the control peptide antigen. Originally used for western blots to prove specificity of protein fractions, this step helps the antibody-peptide complex to settle on the sedimented fraction. We keep the supernatant that in theory should not have any antibody and discard the precipitate. Unspecific binding arises then from fractions in the antibody serum not directed against the epitope.

*C: Omitting the primary antibody:* Slides were incubated with the secondary antibody without applying the primary antibody before. In all cases, the sections were negative and did not present any background staining (data not shown) that would have arisen from the secondary antibodies or visualization system.

*D: isotype matched controls:* In addition, substituting the primary antibodies with isotype matched controls did not give any unspecific staining, concluding that there was background in the staining produced by the primary antibody isotype and the different mammalian tissues.

*E: Negative control tissue:* We performed immunostainings on mouse tissues that do not express these channels (kidney, olfactory epithelium, liver, skeletal muscle and glands).

In addition, Western blot experiments were done by the manufacturer and can be seen at the allomone website ([www.alomone.com](http://www.alomone.com)). Several publications are using these antibodies too:

- guinea pig anti-HCN1 AGP-203 RRID:AB_2756625 (<https://www.alomone.com/p/anti-hcn1-2/AGP-203>). There is one citation using this antibody (Lopez-Gonzalez et al., 2016).

- rabbit anti-HCN2 APC-030 RRID:AB_2313726 (<https://www.alomone.com/p/anti-hcn2/APC-030>). There are 20 citations with this antibody, including KO validations, western blots, immunoprecipitations and immunohistochemistry experiments. For the cochlea previous publications have used it in mouse and rat tissue (Horwitz et al., 2010; Horwitz et al., 2011; Ramakrishnan et al., 2012; Shen et al., 2018).

-rabbit anti-HCN4 APC-056 RRID:AB_2039906 (<https://www.alomone.com/p/anti-hcn4-2/APC-052>).There are 26 citations with this antibody, including KO validations, western blots, immunoprecipitations and immunohistochemistry experiments. For the cochlea previous publications described it in rat and mouse tissue (Horwitz et al., 2011; Shen et al., 2018; Yi et al., 2010)

Since we were advised to reduce our Material & Methods by the editor description, we cannot list all these data here. We add the four sets of supplemental figures for each HCN subunit to highlight the specificity of our stainings and show only the most important specific experiments.

**7. Figure 1. The localization of HCN1 to the AISC and not the AISP is an interesting finding but is not convincingly presented.**

We stay with our description, what we observed in our stainings but agree that interpretation from some section requires correction and relativize this also in the description. In the discussion we therefor state:

page 32, line 2-7: *The AISP is much more difficult to identify, shorter and associated with a thinner peripheral axon. This hampers accessibility for observation. We found only HCN4 in well pronounced AISPs, especially visible in human and guinea pig material. Since we did not examine the AISC and AISP systematically with identifying co-stainings, we cannot provide robust data. The observed HCN4 subunits in long AISPs could act as a pre-filter to favor summation effects with high AP frequencies in some specialized neurons.*

**8. Could the bright labeling in Fig. 1b be a staining artifact? A co‐label with a neuron‐specific antibody would have resolved this issue.**

We have not seen this pattern of staining only once with a single staining technique and a single antibody. A co-staining with other markers was performed such as in the new figure 2b with calretinin but did not change our observation result.

Several points support our observation that neurons in clusters are more stained than more isolated neurons.

1. Several species show the same result. This is clearly visible also in Fig. 1h in human where membranes facing other neurons appear more stained.
2. Changing the antibody concentrations did not change the high staining intensity at adjacent clustered membranes.
3. HCN1, HCN2, HCN4 show the same picture.
4. HCN channels are only present in the neuronal membrane and not in any glia cells. This observation is the same for all subtypes and confirms published data. At electron microscopic level the delicate satellite glia can clearly been identified and was previously shown in several of our publications and from other authors at light- and electron microscopic level.
5. Further, the myelinated neurons in C57Bl/6N showed the same pattern in clusters than unmyelinated neurons in Ly5.1 and human-so any unspecific aggregation due to myelin can be excluded.
6. Light microscopy with DAB chromophore and secondary rabbit-biotinylated antibodies (Figure 1f) results the same at clusters as the confocal image (Figure 1b).
7. Control experiment never exposed unspecific staining in clusters!

In all cases, neuronal membranes in clusters appeared to be more intensely stained than single neurons.

We add some further image here for the reviewer. Together with our new 4 supplemental control set, we hope that we could convince the reviewer that we are not looking at artefacts.

Image 1. HCN1 in Ly 5.1 mice (P23) present high number of clusters and neurons are not myelinated. Neuronal clusters present higher staining at the membrane between adjacent neurons compared to single neurons

Image 2. C57Bl/6N (P16) neurons are already myelinated and present the same pattern of staining with higher intensities for HCN1 at the neuronal clusters compared to single stained neurons. In single neurons the staining is more heterogeneous with weak and strong positive neurons in HCN1 stainings.

-Negative controls using the pre-adsorption peptide did not show any unspecific staining in clusters that could increase the intensity in these neuronal aggregations.

Image 3. C57Bl/6N (P44) staining with the control peptide antigen. No staining is detected in any turn, neither in apical clusters

-Different mice and ages showed these patterns.

Image 4. HCN1 in Ly5.1 (P276) with same dilution as image 1 (Ly 5.1, P23)

Image 5. HCN1 in Ly5.1 (P645) with same dilution as images 1 and 3 (Ly 5.1, P23 and P276).

Image 6. HCN1 in C57Bl/6N (P7) show higher intensity of HN1 in clusters, while single neurons are less stained compared to older ages (Image 2). At this stage of development myelination of soma is in process but not yet fully accomplished.

**9. How is HCN2 labeling distinguished between neuronal membrane labeling and myelin and/or Schwann cell membrane labeling?**

In order to try to convince the reviewer, we add the results from a new experiment. We used directly conjugated HCN2 antibody and combined with a neuronal specific anti-calretinin antibody. With this slide we went to a confocal superresolution microscope (Zeiss 980 airyscan2) and performed z-stacks of plenty of neurons. We were not able to find any reactivity in the Schwann cells, nor in satellite cells, solely the neuronal membrane was positive. The directly conjugated HCN2 antibody further contributed to an exact localization. **We add this image set in the new figure 2,** describe the method of imaging.

We are aware of the fact that DAB development of immunoreactivity may lead to misinterpretation such as the described ion channel is present in neuronal membrane. Confocal imaging can solve this problem but DAB development has some big advantages, such as higher sensitivity (that is why we can see HCN2 in the fibers that requires axonal transport to its destination in the organ of Corti) and stability of staining product. So, sections from many experiments (that were performed with highly standardized methods and automatized imaging and quantification) can be collected and evaluated in one run. This is not possible with fluorescent techniques because stainings fade and stability of aqueously mounted sections is much more limited than with the dehydrated and permanently stable mounted sections of our DAB processes immunostainings.

There is further evidence supporting our results:

1. In myelinated neurons, with interference contrast microscopy the myelin sheath can be visually distinguished from the membrane and was always negative.
2. We support our data in previous publications where HCN channels are described at the neuronal membrane in guinea pig (Bakondi et al., 2009) and mouse (Liu et al., 2014) and our own results in myelinated neurons from guinea pig, cat, C57Bl/6N and CBA/J.
3. Glial cells, satellite glial cell nuclei were always negative in animals and also in human (Fig. 2m).

Some additional images for the reviewer are added here:

Images 7 and 8 show negative Schwann cells in human and image 9 at mouse.

I

I

Image 7. HCN2 staining in SGNs in human. HCN2 staining is located at the membrane of type I neurons (I) and type II neurons (arrow head). Myelinated Schwann cells are negative (arrows) in human.

Image 8. HCN2 staining only at neurons, the myelinated fibers in human representing axonal transport, but not at the Schwann cells nuclei (arrows).

Image 9. HCN2 staining in CBA/J (P48). Positive staining in neuronal membrane, with higher signals in clusters (asterisk). Schwann cells on the nerve fibers are negative (arrows). Satellite glial cells at the neurons are also negative (arrow heads).

^*^

^*^

**10. The authors should justify their correlations between electrophysiological assessments and HCN channel expression given the likely scenario that more than just HCN channels change during these time periods.**

We pointed out also in our initially submitted version of the manuscript that there is a plethora of other factors that influence and cause hearing loss in animals and man (original manuscript, page 29, line 27-33).

In the original manuscript we also pointed out that direct trends are weak for HCN1 (page 18 line 40-41: The age dependent behavior of HCN1 expression showed only weak trends in accordance to eABR recording results.) and HCN4 (page 19, line38-39: Correlation with hearing performance in the two mouse strains is weak). So we did not claim to have found a correlation but we tried to answer what we better expressed now with our 3 hypothesis:

I.) *whether we find differences in HCN expression among mammalian species,*
II.) ***if we can correlate the HCN expression pattern with hearing performance during ageing in mouse strains with different types of age-related hearing loss (ARHL)***
III.) *track the onset and differential expression of HCN channels during mouse developmental stages to correlate with known data on auditory function.*

We more clearly state this in the revised version of the manuscript: page 39, line 4-7.

*Among many other factors, HCN1 and HCN2 channels may be important regulators of SGNs excitability with ageing, whereas the constantly expressed HCN4 subtype does not seem to be involved. We could not find a direct correlation of HCN channel expression level and hearing deterioration that is reflected by different modes of ARHL in C57BL/6 and CBA/J mice.*

So we do not intend to justify something that we have not found. Not each hypothesis proves to be right and stable. But results showed us different things such as up or downregulation of HCN expression dependent on the mouse strain towards the end of their life span.

We removed all functional implications from the abstract and summarize only findings of our stainings. We state also that we found no correlation of hearing performance and HCN expression level in the abstract (page 2, line 14):

*Neuronal diversity in the cochlea is largely shaped by ion channels that control excitability and neuron response. Among voltage gated channels, hyperpolarization-activated cyclic nucleotide-gated (HCN) channels open with hyperpolarization and depolarize the cell until the resting membrane potential. Functions for hearing are not well elucidated and knowledge about localization controversial. We provide a detailed map of subcellular location and co-expression of all four HCN subunits across different mammalian species including CBA/J, C57Bl/6N, Ly5.1 mice, guinea pigs, cats and human subjects and correlate age related hearing deterioration in CBA/J and C57Bl/6N with single cell expression levels of HCN1,2&4 in the same cohort. Spatiotemporal expression exposed HCN2 and HCN4 involvement in a critical phase of hair cell innervation. The huge variety of subunit composition along the perisomatic membrane and axon initial segments highlights an active role at the bipolar spiral ganglion cell bodies especially in neuron clusters where we found staining. Age related changes are diverse as we found HCN channels to be up- or downregulated depending on mouse strain or type of hearing deterioration* ***but no direct correlation with hearing performance****. HCN channels were also found in the organ of Corti in afferent and efferent fibers. The unexpected involvement of HCN channels in outer hair cell function where HCN3 follows prestin location, emphasizes the importance for auditory function. A better understanding may open up new possibilities to tune neuronal responses evoked through electrical stimulation by cochlear implants.*

**11. The potential role of HCN channels in ephaptic coupling is an interesting point for discussion, placing this point as a finding in the abstract and significance is a bit of a stretch.**

The connection between ephaptic coupling and more HCN channel expression in adjacent membranes from neurons in a cluster is removed from the abstract (tracked document page 5, line 15) and significant statement (tracked document page 4, line 5).

**12. The discussion is overly long and highly speculative.**

The discussion was shortened and a summary paragraphs of the results and conclusions added in each section.

In some chapters from the discussion the text was considerably reduced and a short summary included at the end of each chapter when it was necessary. For example in section 4.1.2 Somatic localization of HCN channels (page 29, lines 10-15), 4.1.4 HCN channels in axon initial segments (page 32, lines 8-9), 4.1.5 HCN channels in type II SGNs (page 33, lines 10-11), 4.3 Prehearing, postnatal HCN channels expression in mice (page 37, lines 11-15) and 4.4 HCN channels in ageing (page 39, lines 4-7) and 4.5 HCN channels and electrical stimulation with cochlear implants (page 40, line 23).

References cited in the text:

Bakondi, G., Por, A., Kovacs, I., Szucs, G., and Rusznak, Z., 2009, Hyperpolarization-activated, cyclic nucleotide-gated, cation non-selective channel subunit expression pattern of guinea-pig spiral ganglion cells: Neuroscience, v. 158, no. 4, p. 1469-1477.

Liu, Q., Manis, P. B., and Davis, R. L., 2014, I h and HCN channels in murine spiral ganglion neurons: tonotopic variation, local heterogeneity, and kinetic model: J Assoc Res Otolaryngol, v. 15, no. 4, p. 585-599.

Lopez-Gonzalez, Z., Ayala-Aguilera, C., Martinez-Morales, F., Galicia-Cruz, O., Salvador-Hernandez, C., Pedraza-Chaverri, J., Medeiros, M., Hernandez, A. M., and Escobar, L. I., 2016, Immunolocalization of hyperpolarization-activated cationic HCN1 and HCN3 channels in the rat nephron: regulation of HCN3 by potassium diets: Histochem Cell Biol, v. 145, no. 1, p. 25-40.

Horwitz, G. C., Lelli, A., Geleoc, G. S., and Holt, J. R., 2010, HCN channels are not required for mechanotransduction in sensory hair cells of the mouse inner ear: PLoS One, v. 5, no. 1, p. e8627.

Horwitz, G. C., Risner-Janiczek, J. R., Jones, S. M., and Holt, J. R., 2011, HCN channels expressed in the inner ear are necessary for normal balance function: J Neurosci, v. 31, no. 46, p. 16814-16825.

Ramakrishnan, N. A., Drescher, M. J., Khan, K. M., Hatfield, J. S., and Drescher, D. G., 2012, HCN1 and HCN2 proteins are expressed in cochlear hair cells: HCN1 can form a ternary complex with protocadherin 15 CD3 and F-actin-binding filamin A or can interact with HCN2: J Biol Chem, v. 287, no. 45, p. 37628-37646.

Shen, H., Liu, W., Geng, Q., Li, H., Lu, M., Liang, P., Zhang, B., Yamoah, E. N., and Lv, P., 2018, Age-Dependent Up-Regulation of HCN Channels in Spiral Ganglion Neurons Coincide With Hearing Loss in Mice: Front Aging Neurosci, v. 10, p. 353.

Yi, E., Roux, I., and Glowatzki, E., 2010, Dendritic HCN channels shape excitatory postsynaptic potentials at the inner hair cell afferent synapse in the mammalian cochlea: J Neurophysiol, v. 103, no. 5, p. 2532-2543.

**Reviewer: 3**

Dear Reviewer 3,

We are especially grateful for your comments and critics of our manuscript and the time and effort you took to go through our very complex manuscript-it was a big help for us!

We realized that the big amount of data was presented in a way that is hard to follow for the “non-authors”. We shortened and changed the manuscript fundamentally, addressed your concerns and critics and hope we could considerably improve the text, figures and tables.

We add a “tracked changes” version of the document and hope it can be valuable for the revision process. In our answers to the reviewers we point out main changes and address questions and critics of the editor and reviewer point by point.

As some general critics arouse about our staining specificity, we added several supplemental figures of controls and referenced this also in the text.

Kind regards on behalf of the authors

Rudolf Glueckert & Maria Luque

**Comments to the Author**

**This study examines the expression and localization of HCN channel subunits 1-4 in the spiral ganglion neurons and the organ of Corti across different ages in six animal models, with carefully performed anatomical studies, both regarding HCN channel labeling as well as statistical analysis of the data. This is important work because it is highly likely that HCN channels play an important role in auditory nerve fiber activity, and other neuronal elements in the inner ear. Secondly, there is controversy about HCN channel expression, localization and function in the auditory periphery. The authors report a plethora of findings that differ across ages and animal models in a truly staggering amount of work. The authors report the expression of antibody labeled subunits of the HCN channel in the type I and type II SGN somata, peripheral and central axonal initial segments, type I and type II afferent fibers and in MOC efferent fibers and hair cells. This is a tremendous effort to catalog HCN subunit expression in a generally well-written paper. The main results are 1) specific HCN subunit expression in the immature cochlea; 2)  HCN2 and HCN4 channel expression is prominent in the mature cochlea; HCN3 is found in OHCs 4) HCN channel expression at different ages was not strongly correlated in mice with ABR thresholds. It is important to see that HCN channel expression varies between species.**

**Major concerns:**

**• It is very hard to read the manuscript and to comprehend what the significant message from this study is. The detail and exactness of the results is appreciated, however, the main message gets lost in the detail. Multiple approaches could be taken to improve the readability of the manuscript.**

**Detailed comments;**

**better state the specific goal of the study at the beginning; state the main results in abstract, which is not the case right now. The abstract  trails of on details of channel physiology that do not represent the results presented here in the study**

We removed all functional implications from the abstract and summarize only findings of our stainings (page 2, lines 1-18):

*Neuronal diversity in the cochlea is largely shaped by ion channels that control excitability and neuron response. Among voltage gated channels, hyperpolarization-activated cyclic nucleotide-gated (HCN) channels open with hyperpolarization and depolarize the cell until the resting membrane potential. Functions for hearing are not well elucidated and knowledge about localization controversial. We provide a detailed map of subcellular location and co-expression of all four HCN subunits across different mammalian species including CBA/J, C57Bl/6N, Ly5.1 mice, guinea pigs, cats and human subjects and correlate age related hearing deterioration in CBA/J and C57Bl/6N with single cell expression levels of HCN1,2&4 in the same cohort. Spatiotemporal expression exposed HCN2 and HCN4 involvement in a critical phase of hair cell innervation. The huge variety of subunit composition along the perisomatic membrane and axon initial segments highlights an active role at the bipolar spiral ganglion cell bodies especially in neuron clusters where we found staining. Age related changes are diverse as we found HCN channels to be up- or downregulated depending on mouse strain or type of hearing deterioration but no direct correlation with hearing performance. HCN channels were also found in the organ of Corti in afferent and efferent fibers. The unexpected involvement of HCN channels in outer hair cell function where HCN3 follows prestin location, emphasizes the importance for auditory function. A better understanding may open up new possibilities to tune neuronal responses evoked through electrical stimulation by cochlear implants.*

The authors cut the text considerably! All sections have been shortened and several details on functional implication removed. We are aware that the manuscript is still big. As we did not want to divide this manuscript into a developmental, ageing and immunolocalization paper, we left everything together to provide a comprehensive summary of mammalian HCN channel location and added a more limited discussion on functional implications.

Especially encouraged by the reviewer we expressed 3 hypotheses in the beginning of the manuscript to clearly point out the aim of this paper (page 5 lines 12-18):

*A detailed map of HCN location and expression profiles shall give us answers on:*

*I.)	whether we find differences in HCN expression among mammalian species,*

*II.)	if we can correlate the HCN expression pattern with hearing performance during ageing in mouse strains with different types of age-related hearing loss (ARHL)*

*III.)	track the onset and differential expression of HCN channels during mouse developmental stages to correlate with known data on auditory function.*

The channel physiology summary was removed from the abstract (page 2, lines 1-18): and significance statement (page 3, lines 1-7).

**b) better annotate the figures:**

**As an example: in Fig. 10a,c the strain could be written into the figure, so one can read the meaning of the figure without the legend. Actually the legend is incorrect, and states that a,b show the different strains, but it’s a,c and b,d. Instead of threshold it could say eABR threshold, and instead of amplitude it could say Wave I amplitude. The supplement of Fig. 11 needs to be part of the manuscript, as the statistical analysis is key.**

Figure 6 and 7 were merged together and more labelling added. Titles were written in the upper part of the image with the mouse strain and human specimen, so the reader can catch the data better directly at the figure directly without looking at the legend.

Figure 10 is changed-now **new figure 9.**

Figures a and c represent eABR threshold (dB) vs frequency (KHz) and figures b and d show wave I amplitude (V) vs age (months). In the y-axis for each figure now the strain of mice is written. The legend from figure 10 (**new figure 9**)is corrected (page 59, lines 12-21).

Supplementary figure 1 was added as a **new figure 12** in the manuscript. It was better labelled with letters so we can refer to one of the graphs specifically within the text.

A **new figure 11** was added to show significant different HCN stainings between the turns to expose tonotopical gradients.

Figure legends were also updated: figure 10 (page 59, line 23 and page 60, lines 1-8), figure 11 (page 60, lines 10-16) and figure 12 (page 60, lines 18-24 and page 61, 1-4).

With all the changes and readjustment of figures now the manuscript contains the following figures:

- Figure 1: HCN1 localization.
- Figure 2: HCN2 localization in SGNs
- Figure 3: HCN2 localization in the organ of Corti
- Figure 4: HCN3 localization
- Figure 5: HCN4 localization
- Figure 6: HCN co-expression
- Figure 7: HCN1 and HCN3 during development
- Figure 8: HCN2 and HCN4 during development
- Figure 9: eABR threshold vs frequencies and wave I amplitude vs age in CBA/J and C57Bl/6N.
- Figure 10: HCN quantification of the neuronal staining.
- Figure 11: Statistical significances of the staining between the three turns at each age group calculated from figure 10 (HCN quantification of the neuronal staining).
- Figure 12: Statistical significances between ages in the same turn calculated from figure 10 (HCN quantification of the neuronal staining).

Supplementary figures:

- Figure 1: HCN1 positive and negative controls.
- Figure 2: HCN2 positive and negative controls.
- Figure 3: HCN3 positive and negative controls.
- Figure 4: HCN4 positive and negative controls.

Tables:

- Table 1: primary antibodies used for this study.
- Table 2: secondary antibodies used for this study.
- Table 3: Summary table with the results for HCN stainings in the mammalian cochlea.

**Table  1: One definitely expects a table to review the data, however the table as presented is still hard to read.  Maybe for HCN 1,2,3,4, there need to be subcategories by species, so there is a + for every species if the expression is found, so we see clear boxes with either a “+” a “–“ or a blank. This will give the results that occur generally some weight visually. The second half of table 1 seems out of place in this study, as no physiology was presented, and the entries seem out of context and somehow random. This should be better just reviewed in a paragraph in discussion.**

Table 1 was changed as suggested by the reviewer with all the species included and ++, - or NA (when something was not evaluated) to summarize the expression levels of each HCN subunit in each specie tested.

Table 2 was removed from the manuscript as the authors share the opinion of better summarize the possible functions in the discussion section.

**c) there are too many grammatical errors and typos in the manuscript**

We corrected many errors.

**d) Detailed information and important results are mixed in a way that make it hard to get the main message. It would be helpful to have short summaries of the main results (not repeat of all results) at the end of the individual chapters. This is already implemented in some chapters**

Summaries in result section were improved or added: 3.1.1 HCN1 localization (page 14, lines 1-3), HCN2 localization (page 15, lines 13-19), HCN3 localization (page 16, lines 17-20), HCN4 localization (page 17, lines 22-26 and page 18, lines 1-2), 3.2 HCN subunit co-expression and correlative distribution in SGNs of mice and man (page 20, 2-7), 3.3 Distinct pattern of HCN channel expression in mouse postnatal development until the onset of hearing (page 21, lines 25-26 and page 22, lines 1-5), 3.4.2 HCN Quantification (page 26, lines 10-20)

In all chapters from the discussion the text was considerably reduced and a short summary included at the end of each chapter when it was necessary. For example in section 4.1.2 Somatic localization of HCN channels (page 29, lines 10-15), 4.1.4 HCN channels in axon initial segments (page 32, lines 8-9), 4.1.5 HCN channels in type II SGNs (page 33, lines 10-11), 4.3 Prehearing, postnatal HCN channels expression in mice (page 37, lines 11-15) and 4.4 HCN channels in ageing (page 39, lines 4-7) and 4.5 HCN channels and electrical stimulation with cochlear implants (page 40, line 23).

**e)The discussion reads like a separate review article on HCN channel function in general and in the inner ear. It is comprehensive and insightful, but also highly speculative and trails off quite far from the data presented here and the discussion of the data in the context of the literature. Some readers may be grateful for this excellent summary of the state of the field, however, it is hard to find out again, what were the important results of the paper and what needs to be critically discussed regarding the data.**

We cut the whole discussion considerably! And added short summaries of the conclusions when necessary (section d) review questions).

The result section is also modified including number of figures according to the changes made during the revision process and following the suggestions of the reviewer.

**This may be more of a personal choice on how to embed one’s results into the field, however, these reviewers felt they would have been better served with a much shorter but concrete discussion that points to the main results. A critical assessment of the labeling, imaging and analysis methods used is provided throughout the paper, but could have been summarized in a concise way in the discussion. In our opinion, most parts of discussion should be separated from the paper and published as a review article. The insight, that the understanding of HCN channel function will help with the design of cochlear implants is appreciated and shared by the reviewers.**

Discussion was shortened and re-structured.

We cut also the description of the material and methods considerable and think it is still conclusive and detailed enough to grant transparency of getting our data.

• **The authors include functional audiometric data at different time points to correlate audiometric function with the expression of HCN channels. This part is weak, as it is just a descriptive comparison. As there are numerous other factors that contribute to an increase in audiometric threshold and a decrease in response amplitude with increasing age, so such a connection is tenuous at best.**

We pointed out also in our initially submitted version of the manuscript that there is a plethora of other factors that influence and cause hearing loss in animals and man (original manuscript, page 29, lines 27-33). In the new revised manuscript we maintain this sentence (page 37, lines 18-21).

In the original manuscript we also pointed out that direct trends are weak for HCN1 (page 18 line 40-41: The age dependent behaviour of HCN1 expression showed only weak trends in accordance to eABR recording results.) and HCN4 (page 19, line38-39: Correlation with hearing performance in the two mouse strains is weak). So we did not claim to have found a correlation but we tried to answer what we better expressed now with our 3 hypothesis in page 5 line 14-18 (Thanks to you!):

I.) *whether we find differences in HCN expression among mammalian species,*
II.) ***if we can correlate the HCN expression pattern with hearing performance during ageing in mouse strains with different types of age-related hearing loss (ARHL)***
III.) *track the onset and differential expression of HCN channels during mouse developmental stages to correlate with known data on auditory function.*

We more clearly state this in the revised version of the manuscript: page 39, lines 4-7.

*Among many other factors, HCN1 and HCN2 channels may be important regulators of SGNs excitability with ageing, whereas the constantly expressed HCN4 subtype does not seem to be involved. We could not find a direct correlation of HCN channel expression level and hearing deterioration that is reflected by different modes of ARHL in C57BL/6 and CBA/J mice.*

So we do not intend to justify something that we have not found. Not each hypothesis proves to be right and stable. But results showed us different things such as up or downregulation of HCN expression dependent on the mouse strain towards the end of their life span.

We removed all functional implications from the abstract and summarize only findings of our stainings. We state also that we found no correlation of hearing performance and HCN expression level in the abstract (page 2, line 14):

*Neuronal diversity in the cochlea is largely shaped by ion channels that control excitability and neuron response. Among voltage gated channels, hyperpolarization-activated cyclic nucleotide-gated (HCN) channels open with hyperpolarization and depolarize the cell until the resting membrane potential. Functions for hearing are not well elucidated and knowledge about localization controversial. We provide a detailed map of subcellular location and co-expression of all four HCN subunits across different mammalian species including CBA/J, C57Bl/6N, Ly5.1 mice, guinea pigs, cats and human subjects and correlate age related hearing deterioration in CBA/J and C57Bl/6N with single cell expression levels of HCN1,2&4 in the same cohort. Spatiotemporal expression exposed HCN2 and HCN4 involvement in a critical phase of hair cell innervation. The huge variety of subunit composition along the perisomatic membrane and axon initial segments highlights an active role at the bipolar spiral ganglion cell bodies especially in neuron clusters where we found staining. Age related changes are diverse as we found HCN channels to be up- or downregulated depending on mouse strain or type of hearing deterioration* ***but no direct correlation with hearing performance****. HCN channels were also found in the organ of Corti in afferent and efferent fibers. The unexpected involvement of HCN channels in outer hair cell function where HCN3 follows prestin location, emphasizes the importance for auditory function. A better understanding may open up new possibilities to tune neuronal responses evoked through electrical stimulation by cochlear implants.*

• **The ion channel co-expression analysis is unclear and needs to be better explained**

The figure was modified.

revised figure 6

We have joined figure 6 and 7 as we thought that maybe is more intuitive to look at the image at the same time as the marginal histograms representing the % of intensity for each channel at the neuronal membrane (**new Figure 6**).

The text for the result section 3.2 HCN co-expression in SGNs (original document page 14, line 42 to page 16, line 10) and figures legend 6 and 7 (original document, page 43) was also updated and written clearer. We introduced a new headline and section **3.2 HCN subunit co-expression and correlative distribution in SGNs of mice and man** (page 18, lines 4-26 to page 20, lines 1-7) and figures legend also updated and written clearer in a new legend for new figure 6 (page 57, 4-25).

**• Were the animal species/strains raised under the same conditions? Different noise exposure in the animal facility may possibly affect the gene expression pattern in the inner ear. It might be helpful to allow comparison with other groups to measure the sound exposure and add this to methods, or at least to state if the different mouse models/ animal models were raised under the same conditions. This could account for expression differences besides species/strain differences.**

This is a very good point to address. Animals were raised exactly under the same conditions. The two different strains shared the same room and they are located at the same structure along different racks, so there should not be any difference in noise exposure or other parameters. Comparing our data with other groups, we conclude the trends are the same. Hearing loss in C57Bl/6N is seen after 4 months of age

We added this to the text:

(page 7, lines 1-2): All mice used here were bred in the same room at the animal facility in Innsbruck with a 12 h dark, 12 h light cycle and unlimited access to food and water.

And in (page 23, line 15-17): Since breeding of both animal strains was done in the same room, a difference in noise exposure can be excluded to bias our measurements.

• **Page 17; Line 48: the statement that wave I amplitude indicates the number of SGNs firing is not completely correct. It would also indicate synchrony of firing, and the same number could provide different amplitudes with different levels of synchronous firing. It would also be helpful to have statistical test result annotated in the figure, so one can see which decline in amplitude is statistically significant.**

The text from the original document was changed (page 17, line 48) and the information given by the reviewer added in the new document (page 22, line 24). Citation about synchronous firing was also incorporated.

Old figure 10 was changed and statistical significances included in a table were the reader can see different statistical significances represented by a color code in each frequency for different ages. The eABR thresholds and wave I amplitudes graphs for both strains present now underneath a new summary table with the same format as the old supplementary figure 1.

revised figure 10

• **For the comparison of HCN channel expression and ABR threshold/wave I, plots should be presented with the same x axis (age), so one can visually compare trends. It is difficult to compare Figs. 10 and 11.**

We combined the hearing thresholds with HCN quantification to provide a better way to see possible correlations. However, we did not include the wave I amplitudes there because we would lose a clear arrangement of data. Since wave I amplitude do not change significantly in C57Bl/6N mice and only between young and eldest group we regard this as acceptable.

• **Pge 18, HCN quantification. Although stated in methods, here the main points of the quantification method should be shortly introduced. It needs to be stated, which regions were quantified (SGN somata?). How were the data normalized between ages? And species? The suppl/ figure 1 needs to be explained and introduced in the text (it should not be suppl., but part of the main manuscript). What was tested here against what? It is confusing because one expects to see a test HCN expression versus ABR data.**

All steps for quantification were highly standardized and many steps automatized. Mice were exposed bred under the same conditions during their lifespan. Tissue was collected and embedded following the same protocols. Staining experiments were performed in an automatized VENTANA device immunostainer and antibody dilution was the same too. Segmentation and data extraction were highly automatized and user independent. Also, consecutive sections from the same animal were used in each quantification for each strain.

Therefore, we did not normalize the data as the idea was to also show the differences in staining intensities between the two different strains of mice.

The trends obtained for the three HCN channels antibodies were different in the two mouse strains and also staining levels differed for HCN1 and HCN2, but not for HCN4.

In the abstract we state:

page 2: lines 5-9: We provide a detailed map of subcellular location and co-expression of all four HCN subunits across different mammalian species including CBA/J, C57Bl/6N, Ly5.1 mice, guinea pigs, cats and human subjects and **correlate age related hearing deterioration in CBA/J and C57Bl/6N with expression levels of HCN1,2&4 in the same cohort**

In the first line of the description of methods we stated:

page 10: lines 10-12: The **membrane staining around the SGN somata was quantified** using an automated and calibrated microscope system (TisseFaxPlus®) with a dedicated image analysis software (Histoquest®, RRID:SCR_014823, Tissuegnostics®, Vienna, Austria).

Negative controls were also performed in the same sections for three different groups including pre-hearing mouse specimens (from 0 to 14 days old), normal adult age (from 15 to 29 days, less than 1 month) and older ages (from 15 to 19 months). The controls were done with the control peptide antigen to quantify possible background staining coming from the tissue. Images were taken with automated and calibrated microscope system (TisseFaxPlus®) and analyzed with the image analysis software Histoquest®, using always the same parameters as the ones applied for the quantification of the neuronal staining. In all cases the background staining was subtracted with these controls we could be sure that differences of the staining was not due to unspecific recognition of the antibody in different aged specimens.

Supplementary figure 1 was added as a new figure 12 in the main text. It is also explained now in the text and in the figure legend (page 60, lines 18-24 and page 61, lines 1-4).

**ANSWERS to the EDITOR**

**Associate Editor: Nolan, Lisa**

**Comments to the Author:**

**This is a really interesting study, I read the manuscript with great interest. At present the spatiotemporal expression pattern of the HCN channel components in the cochlea is not clear. Given the role of these channels in auditory sensitivity, I strongly encourage the authors to consider the effect of sex as a biological variable (SABV) on the tonotopic expression pattern of these channels in the cochlea. The age-related loss of hearing in humans is modulated by SABV. Women have a tendency for a greater hearing sensitivity than men at the high-frequencies throughout the lifespan. In comparison, men have a tendency for a greater hearing sensitivity in low-frequencies compared to women. Therefore, more information on whether comparisons are sex-matched would be helpful – where this information is available. This could either be added to the figures/legends or discussed/acknowledged in the manuscript. One should also include somewhere if the methodologies employed will detect all isoforms of the various channel components.**

The effect of sex as a biological variable (SABV) is a fundamental necessity since we gain more and more knowledge about sex difference as a significant variable in the hearing field. We were aware of that fact and checked our initial data but we could not find any indication within our animal and age groups that we tested systematically (C57Bl/6N and CBA/J mouse strains). Also, in a retrospective way we tested our data with the same result (page 6, lines 22-25 and page 7, line 1).

This may be due to the fact that we did not test frequency ranges above 32kHz where these effects become evident. Our free-field measurements may imply some limitations but provide robust results in the range that we measured. Doing any kind of acoustic stimulation in the frequency range above 40kHz sets formidably demands on the orientation of the source of sound and pinna orientation. Since we did not cut the pinnae and use stereotactic equipment to guarantee reproducible data, we limited ourselves to a frequency range that covers some high frequency regions but does not cover the whole basal turn of a mouse. We address this in the text with a rough allocation of the 32kHz to the upper basal turn. We further added some citations that support our decision not to consider sex effects at page 6 in section 2.1 of the manuscript:

Page 6, line 22-25: There were no gender differences in age related hearing performance found in C57Bl/6N strains until 32 kHz (Hunter and Willott, 1987). In CBA/J no difference in hearing deficits were previously found until 18 months of age, when females showed a faster deterioration of hearing than males (Ohlemiller et al., 2010).

**At present the key messages of the manuscript are lost within the amount text. The text needs to be cut considerably – the manuscript is far too long and the discussion beyond the scope of the data. Please also note here the reviewer comments on readability. The methods section can be cut in half by citing standard methodologies. As also suggested by one of the reviewers, the authors should consider using parts of their interesting discussion for a mini-review on this topic.**

The authors cut the text considerably! All sections have been shortened and several details on functional implication removed. We are aware that the manuscript is still big. As we did not want to divide this manuscript into a developmental, ageing and immunolocalization paper, we left everything together to provide a comprehensive summary of mammalian HCN channel location and functional implications.

**The reviewers have also raised several concerns and these need to be addressed. Therefore, at present I cannot accept the manuscript for publication in JNR in its current form.**

**Additional points**

**Page 5, line 47. The authors should acknowledge that the frequencies used for the ABR recordings in the C57’s are lower than the frequencies used by Henry et al., (2002, 2004) when sex-differences in hearing thresholds were observed in this mouse strain.**

The ABRs done by Henry et al. are extraordinary in a way to reproducibly record in very high frequencies using stereotactic equipment to grant very exact positioning. We are aware of the challenge to exactly and reproducibly position the sound source and ears since slightest variations change the outcome dramatically. This is even more challenging in a free field stimulation like we do. Pinna position and exact orientation of the loudspeaker is very critical in the high frequencies and we did not remove the pinna like some scientist do. This is why we did not get higher than 32kHz with measurements in our custom-made system where we use only one speaker that we change in position when we measure the other ear of a mouse. Our more than 10 years’ experience with our custom-made system let us conclude that we can safely and reproducible measure not higher than 40 kHz and stayed on the safe side with 32 kHz.

Going to higher frequencies with our ABR system was not recommended for our custom-made system because there are more factors that can influence threshold measurement and bias the data. For this reason, we have just performed measurements until high frequencies of 32 KHz, lower than in the paper of Henry et al, 2002 where 56 kHz frequencies were also tested.

A clarifying sentence about the frequencies used in this study compared to previous ones was added in page 6, lines 22-25.

**Additional Editorial comments:**

**Please remove the list of abbreviations, this is not a format accepted by JNR, abbreviations must be provided and spelled out the first time they are used in both the abstract and in the main text.**

The list of abbreviation was removed from the final text. Abbreviations are always explained at the first time the word appears in the main text and used throughout the text.

**In the figure legends, where appropriate please add the number of animals/group and the statistical test used.**

Figure 9 legend (old figure 10): the minimum number of animals used in each group of age is included (page 59, line 15). Statistic tests applied are also included (page 59, lines 17-21).

Figure 10 legend (old figure 11): the total number of mice used for each strain is included (page 60, line 3). Statistic tests applied are also included (page 60, lines 6-8).

**Please provide the total number of animals/sex and strain used in this study.**

Total number of animals for CBA/J and C57Bl/6N used is listed in page 7, line 2 (material and methods section, 2.1 Animals). In total, 65 CBA/J and 84 C57Bl6/N were used for this study.

**GRAPHICAL ABSTRACT**

**Please upload a graphical abstract, which we are asking of all authors submitting original research articles. This is intended to provide readers with a visual representation of the conclusions and an additional way to access the contents and appreciate the main message of the work. What we require is a .tif image file and a .doc text file containing an abbreviated abstract. For the image, labels, although useful, must be kept to a minimum and the image should be 400 x 300, 300 x 400, or 400 x 400 pixels square and at a resolution of 72 dpi. This can be one of the figures from your article, or something slightly different, as long as it represents your study.  Instructions for this can be found in our author guidelines online at** <http://onlinelibrary.wiley.com/journal/10.1002/(ISSN)1097-4547/homepage/ForAuthors.html>

We added a graphical abstract and present it here:

All HCN channel subtypes were located at subcellular level in human and compared with other mammals such as guinea pig, cat and 3 mouse strains. Changes in HCN expression levels were quantified in ageing in mice and postnatal development. The widespread expression and distinct localization emphasize the importance of HCN channels for sound coding and electrical stimulation with cochlear implants.

**Please include a table where, in accord with JNR requirements, you provide additional information on each of the antibodies that you use. This must include the source (not just the manufacturer and catalog or lot number, but the species it was raised in, and the EXACT structure of the immunizing antigen - including the amino acid sequence for peptide antigens); characterization (e.g., what does it recognize on immunoblots); and appropriate controls (i.e. the effect of blocking peptides for immunohistotochemical localization).  The RRID can be included here as well and removed from the methods**.

We added a new table 1 and table 2 to summarize the antibodies and their characteristics used in this study. Table 1 summarized the data concerning the primary antibodies including immunogen peptide sequence, manufacturer, RRID, catalogue number, lot number, host species, clone number and working dilution. Table 2 summarized the data for the secondary antibodies employed with the target specie recognized by the antibody, host specie where the antibody is raised, antibody isotype specificity, manufacturer, RRID, catalog number, lot number, number of clones and working dilution.

**SEX DIFFERENCES**

**The National Institutes of Health now mandates the inclusion of sex as a biological variable. To conform with this mandate, the Journal of Neuroscience Research has established new policy (please see our editorial:** [**http://onlinelibrary.wiley.com/doi/10.1002/jnr.23979/full**](http://onlinelibrary.wiley.com/doi/10.1002/jnr.23979/full)**) requiring all authors to ensure proper consideration of sex as a biological variable. Please ensure that: 1) Any paper utilizing subjects of one sex state the sex of the sample in the title and abstract; 2) The number of samples/subjects of each sex used in the research must be clearly stated in the methods section; 3) The inability for any reason to study sex differences where they may exist should be discussed as a study limitation. We are also encouraging authors to report exploratory analyses of potential sex differences in studies not explicitly designed to address them.**

For the ABR data the number and sex of mice used was:

-CBA: 28 mice (15 males and 13 females).

-C57Bl/6N: 49 mice (6 males and 10 females)

Mice are distributed unevenly regarding the gender among groups since we did not find any indication for any gender differences regarding our study. This decision was supported by already published data that described sex differences in a frequency range that we did not cover with our electrophysiological measurements.

For the quantification data the number and sex of mice used was:

-CBA: 37 mice (20 males, 14 females and 3 mice not identifiable due to very young ages-pre-hearing animals distributed unevenly in the different age groups).

-C57Bl/6N: 36 mice (19 males and 17 females distributed unevenly in the different age groups)

We have not considered sex differences as for the aim of this study we regarded it as not relevant. In the literature only very old ages above 18 months for CBA/J (Ohlemiller et al., 2010) were reported to uncover sex differences in hearing deterioration. Some authors have found sex differences only in very high frequencies up to 56 KHz. Since acoustic stimulation in this frequency range requires very sophisticated equipment to grant reproducibility, we stimulated at 32 kHz as the maximum. There were no gender differences in age related hearing performance found in C57Bl/6N strains until 32 kHz (Hunter and Willott, 1987). Differences between sex were tried to address between mice with a direct comparison female-male with Kendall tau co-relation and data was not conclusive due to intrinsic differences between individual mice (above all at older ages). This provided for us data not to considerably rise the number of mice used in this study. To consider the 3Rs of animal experimentation we limited the number to a minimum without considering gender effects.

References cited in the text:

Hunter, K. P., and Willott, J. F., 1987, Aging and the auditory brainstem response in mice with severe or minimal presbycusis: Hear Res, v. 30, no. 2-3, p. 207-218.

Ohlemiller, K. K., Dahl, A. R., and Gagnon, P. M., 2010, Divergent aging characteristics in CBA/J and CBA/CaJ mouse cochleae: J Assoc Res Otolaryngol, v. 11, no. 4, p. 605-623.

_____________________________________________________________________________________

**2^nd^ Editorial Decision	09-Aug-2020**

Dear Dr Glueckert:

Thank you for re-submitting your manuscript to the Journal of Neuroscience Research. We've now received the reviewer feedback and have appended those reviews below. While reviewers and the editors are overall very enthusiastic and supportive of the study, there are still some concerns, most importantly the manuscript will greatly benefit from a thourough revision of the language by a native English speaker, and some more trimming of the lenghty test is necessary. JNR does not have words limits, except for the abstract and the significance statement, still the manuscript is too verbose and the main message is often lost. The points listed below should be relatively straightforward to address. If there are any questions or points that are problematic, please feel free to contact me. I am glad to discuss.

We ask that you return your manuscript within 30 days. Please explain in your cover letter how you have changed the present version. If you require longer than 30 days to make the revisions, please contact Dr Cristina Ghiani (cghiani@mednet.ucla.edu). You can submit your revised manuscript directly by clicking on the following link: *** PLEASE NOTE: This is a two-step process. After clicking on the link, you will be directed to a webpage to confirm. ***

https://mc.manuscriptcentral.com/jnr?URL_MASK=a456f54cb0d14fba8ce959dccd0ccae5

Thank you again for your submission to the Journal of Neuroscience Research; we look forward to reading your revised manuscript.

Best Wishes,

Dr Lisa Nolan

Associate Editor, Journal of Neuroscience Research

Dr Cristina Ghiani

Co-Editor-in-Chief, Journal of Neuroscience Research

Associate Editor: Nolan, Lisa

Comments to the Author:

I would like to thank the authors for their attempt to address the concerns of the reviewers and I greatly appreciate the effort to which the authors have gone to address these concerns. However, at present many ambiguous sentences and grammatical errors remain and these unfortunately make the manuscript very hard to read with clarity in places. I strongly recommend that the authors have the manuscript proof-read by somebody fluent in English Grammar and edit the manuscript aggressively before submitting any further revision to JNR. Additionally, although I appreciate that the authors have cut the text of the manuscript, further aggressive trimming of the text would also improve the readability.

Minor points

Age related - where it appears in the manuscript should be written as age-related.

Abstract

Line 9: ….single cell expression levels HCN1,2&4……

 The manuscript does not contain single-cell qPCR data / single-cell RNA-seq data etc; therefore, please refrain from using the term single-cell expression in the current context as it is confusing.

Significance statement

Line 6: ….suggests HCN channels to be involved as an active component.

 Active component of what? Please re-phrase.

Introduction

Line 6: -820 of these…

 Rewrite this sentence so you do not begin the sentence with 820 in numeric form as it is currently written.

Line 14-20

 These 3 aims would fit better at the very end of the introduction. They would also benefit from being written more precisely. For example: Here, we provide a detailed map of HCN channel subunit expression in the auditory system, and address whether:

o i) HCN channel subunit expression in the cochlea varies among different mammalian species,

o ii) etc etc

Page 43, Line 21-26 & page 44, Line 1-5

 This section would fit better before the 3 aims are listed.

Sex as a biological variable (SABV)

Vital information on SABV is still missing or not addressed.

 The data from humans, cats and guinea-pigs although interesting, appears limited as the age and sex is not reported. Granted the authors have explained that this was not known for the human subjects, but what about the cats and guinea-pigs? Consequently, I am not sure the comparisons to HCN channel subunit expression in mice are helpful as it is difficult to draw clear conclusions across various species when this information is not reported / not known for the other species. If this information is not known, then please kindly address and discuss this limitation of the study in the discussion.

 Care should be taken to consider sex as a biological variable when performing wave l comparisons. In humans, females display larger wave l amplitudes compared to males. There is also some evidence that female mice exhibit larger wave l amplitudes compared to males. See the following papers:

Letter to the editor: Examination of potential sex influences in. Auditory function in normal-hearing, noise-exposed human ears, ear hear, 36, 172–184. Ear and Hearing, 36(6), 738–740. https://doi.org/10.1097/AUD.00000 00000 000228

The impact of biological sex on the response to noise and otoprotective therapies against acoustic injury in mice. Biology of Sex Differences, 9(1), 12.

 The sex breakdown for the mice used in this study as given in the response to reviewers needs to be incorporated into the methods section of the paper. Ideally with the sex breakdown for the cats and guinea-pigs too – if known?

Additional comments

 Please keep the extra control images as supplementary data.

Kindly mark the changes made to the text using a red and/or bold font, please do not use the red highlight.

As suggested/requested in the instructions to the authors kindly change the red/green images to magenta/green, so that "the work accessible to the approximately 6-10% of male readers who have red-green color blindness."

Reviewer: 4

Comments to the Author

The manuscript by Luque & colleagues has been resubmitted following apparent extensive changes to the text and figures. I have not seen the original submission, and so I am restricted to comments on the current version.

The study is an interesting one, that describes the distribution of HCN channel family members in the cochlea of various species (including human temporal bone samples), and mice at of several strains at various ages. As such it includes a huge volume of data, and while this will represent an important resource for the field, it is confusing in places due to the amount of descriptive data and the lack of similarities between the species/strains.

Attempts have been made to provide controls to address questions on antibody specificity. Positive controls on other tissues are suitable, as are negative controls using pre-absorption using antigenic peptides and omitting primary antibodies. Ideally, the antibodies would also be validated on knockout tissue, and on cell-lines expressing the individual channel types. These additional controls would allow the reader to appreciate how specific the antibodies are, and whether they truly distinguish between the channel family members.

At the end of the study there are attempts to relate relative expression to hearing ability. This adds to the confusion as there are no clear relationships on show. Although semi-quantitative analysis of staining intensity has been carried out, I’m not convinced that “statistical significance” can always be translated directly into “biological significance”, i.e. whether measureable differences in staining intensity for a single channel type can be seen to have influence on complex electrophysiological or behavioural outputs.

Major concerns

1. The emphasis of antibody staining at axon initial segments is unconvincing and somewhat arbitrary. It is impossible to determine where these regions are without counterstains. A number of antibodies for nodes (e.g. Nav1.6, ankyrin G, Kv3.1), paranodes (Caspr) or juxtaparanodes (Kv1) are available to allow these specialized regions to be identified (Hossain et al, 2005; Kim & Rutherford, J Neuroscience 2016).

These parts of the manuscript should be deleted.

2. The attempt to relate staining intensity to hearing thresholds should be removed. The graphical representation of the staining intensities may be helpful to some readers, and they emphasise the amount of work within the study, but they are over-complicated by the overlays of eABRs.

Minor concerns

Page 5, line 7: typo “several”

Page 6, line 17: Ministry of Science

Page 13, line 20: I don’t understand the statement “Tonotopical gradients are not easily determinable since even within different mouse strains and age, gradients may change (semi-quantified in Fig. 10 and 11).” Please rewrite.

Page 15, line 6 & 8: typo “caliber” not caliper

Page 16, line 15: typo “void” not “avoid”

Page 29, line 23: typo “In contrast”

Reviewer: 3

Comments to the Author

This study has a great scientific merit and greatly contributes to our understanding about HCN channel expression in the auditory periphery and the authors have done much to improve the manuscript. The scientific approach used in this manuscript is solid and the data presented appear sound. The inclusion of goals in the introduction, summaries at the end of sections, and a graphical abstract improves the understanding and impact of this manuscript. However, the manuscript could still be improved markedly on two points. Some minor comments are included that would further streamline the manuscript but these comments are left to the discretion of the authors.

Main comments:

1) The authors should carefully look over their word usage throughout the manuscript. The meaning of some words is either not specific enough for the meaning of the statement the authors wish to convey or seems that sometimes the wrong word is used for the context. A few examples are given below but there are more throughout the manuscript. By making these key words more specific the authors will avoid confusion in the statements they make about their science.

a) Line 1 pg. 28 (pg. 66 of the document): “The level of dendritic HCN4 expression varied considerably among species and partially faded after hearing onset while HCN2 persisted.”

b) Same page line 6: “A fast instantaneous component of Ih observed by pulsed hyperpolarization turned out to grow maximal in the slow HCN3 and HCN4 channels.”

c) Same page line 25: “The soma of bipolar neurons increases the capacity that theoretically decrease amplitudes and delay signal propagation between peripheral and central axon as we modeled previously (Rattay et al., 2013).”

2) The authors should clarify where the data for their tonotopic expression analysis comes from. The authors have done a great job in including and clarifying an analysis on the tonotopic expression of the HCN channels as can be seen in figures 10-11-12. However, it is unclear where this data comes from. Possibly from figure 7 and 8? Please clarify and show how the tonotopic expression data for the various HCN channels is obtained.

Minor suggestions that the authors could consider including:

The panels in the figures are rather small which makes it difficult to see details described in the text by the authors.

The way that figures 11&12 are presented only indicates whether there is a significant difference but not which direction the difference is in. It would be beneficial to either show examples of histochemical images or indicate in the current figures which of the HCN channels (fig 11) or which tonotopic region (fig12) is the largest.

Several times the authors mention a stronger labeling in Ly5.1 mice but it is not sufficiently discussed why that would be the case or if this is important.

For some statements it is unclear for which species or strain of mouse the statement is made. This leads to confusion especially when the authors wish to present a point about the general function of a certain ion channel in a certain location.

For figure 6 it is unclear how the analysis of the fluorescence intensity contributes to quantifying colocalization of the various HCN channels.

*********IMPORTANT: Instructions and checklists follow*********

When finalized, please upload your complete revised manuscript onto our website, preferably as a word document. Please ensure to upload a highlighted/marked version of your manuscript along with the clean version. The marked version should have the revised text or any other changes made to the manuscript in a red font. The clean version should have no marked sentences, strike-through words, or comments in margins. Kindly avoid submitting a document with tracked changes. Figures must be uploaded separately in .tif or eps format. Please review our submission checklist, which can be found in our author guidelines and also be sure to fill out the Transparent Science Questionnaire attached to this email.

JNR offers Open Science badges to qualifying authors. For more information please see the “Open Science initiatives” section of our author guidelines. If you would like to apply for one or more of the badges, please complete the included disclosure form and upload it as Supplemental Material Not for Review when submitting your final manuscript files.

To submit your revised manuscript:

*You have two ways to submit your revision. Either click on the link: *** PLEASE NOTE: This is a two-step process. After clicking on the link, you will be directed to a webpage to confirm. ***

https://mc.manuscriptcentral.com/jnr?URL_MASK=78727a0569eb4bee8806e57a90ad685b or Log on to https://mc.manuscriptcentral.com/jnr using your case-sensitive User ID ((Person not available)).

For security purposes your password is not listed in this email. If you are unsure of your password you may click the link to set a new password.

(Person not available)

*Enter the Author Center, where you will find your manuscript title listed under “Manuscripts with Decisions”

* Under “Actions,” click on “Create a Revision”. Your manuscript number has been appended to denote a revision.

*Follow the prompts and replace existing files with revised ones, as necessary.

If you encounter any troubles in submitting your revised manuscript, please contact Dr Cristina Ghiani (cghiani@mednet.ucla.edu) or click on Get Help Now at the top right of any ScholarOne Manuscripts screen.

_____________________________________________________________________________________

**Authors’ Response	11-Sep-2020**

Reviewer: 3

Dear Reviewer 3,

First of all, thank you again for your effort to read and comment our manuscript. The authors´ are grateful for the new ideas brought in. We appreciate your contribution that shall increase the quality of the manuscript. The suggested changes were applied and addressed point by point below.

Kind regards on behalf of the authors,

Rudolf Glueckert & Maria Luque Calvo

Comments to the Author

This study has a great scientific merit and greatly contributes to our understanding about HCN channel expression in the auditory periphery and the authors have done much to improve the manuscript. The scientific approach used in this manuscript is solid and the data presented appear sound. The inclusion of goals in the introduction, summaries at the end of sections, and a graphical abstract improves the understanding and impact of this manuscript. However, the manuscript could still be improved markedly on two points. Some minor comments are included that would further streamline the manuscript but these comments are left to the discretion of the authors.

Main comments:

1) The authors should carefully look over their word usage throughout the manuscript. The meaning of some words is either not specific enough for the meaning of the statement the authors wish to convey or seems that sometimes the wrong word is used for the context. A few examples are given below but there are more throughout the manuscript. By making these key words more specific the authors will avoid confusion in the statements they make about their science.

We have carefully checked the manuscript and changed plenty of sentences to make it more precise and readable (changes are marked in red font in the text). In the end we sent the main text to a professional language editing company (Scribendi, Canada) to correct for the English grammar, comprehension, spelling and style.

Sentences listed by the reviewer were also re-worded or written new:

a)	Line 1 pg. 28 (pg. 66 of the document): “The level of dendritic HCN4 expression varied considerably among species and partially faded after hearing onset while HCN2 persisted.”

This statement was removed when shortening the text.

b)	Same page line 6: “A fast instantaneous component of Ih observed by pulsed hyperpolarization turned out to grow maximal in the slow HCN3 and HCN4 channels.”

“A fast instantaneous component of Ih observed with pulsed hyperpolarization was found to grow maximal in HCN3 and HCN4 channels that presented the slowest activation kinetics.” (page 23, lines 1-3)

c)	Same page line 25: “The soma of bipolar neurons increases the capacity that theoretically decrease amplitudes and delay signal propagation between peripheral and central axon as we modeled previously (Rattay et al., 2013).”

“The soma of bipolar neurons increases the capacitance that theoretically decreases amplitudes and delay signal propagation between peripheral and central axon as we modelled previously (Rattay et al., 2013).” (page 23, line 17)

2) The authors should clarify where the data for their tonotopic expression analysis comes from. The authors have done a great job in including and clarifying an analysis on the tonotopic expression of the HCN channels as can be seen in figures 10-11-12. However, it is unclear where this data comes from. Possibly from figure 7 and 8? Please clarify and show how the tonotopic expression data for the various HCN channels is obtained.

The data for the tonotopic expression were extracted from the semi-quantification of the neuronal staining at different age groups in figure 10. We have rewritten this section and add a sentence from where the statistic was performed:

Page 20, lines 8-18: “Semi-quantitative evaluation of HCN1, -2, and -4 staining intensities from newborn to 19 months old C57Bl/6N and CBA/J mice of individual SGNs revealed only a few corresponding trends with eABR recordings (Figure 10)…. Statistically significant differences are presented among the turns within one age group (Figure 11) and among the same turn at different ages (Figure 12).”

We also cited the figure when we talked about tonotopic gradients in other sections, for example:

Page 14, lines 19-21: “There was a tonotopical gradient visible in young C57Bl/6N (Figures 5a, 10f, 11b) but semi-quantification of the staining exposed that this grading was not constant (Figures 10f, 11b) throughout lifetime.”

Page 25, lines 5-18: ¨Semi-quantitative evaluation of HCN1, -2 & -4 staining intensities from newborn to 19 months old C57Bl/6N and CBA/J mice revealed only few corresponding trends with eABR results (Fig. 10)…. Kruskal- Wallis non-parametric method was applied to study differences between the turns within one age group (Fig. 11) and between the same turn at different ages (Fig. 12)¨.

Minor suggestions that the authors could consider including:

The panels in the figures are rather small which makes it difficult to see details described in the text by the authors.

We changes several figures due to new data that we included. Some other figures were removed. The PDF version generated for the review process is rather low resolved, so zooming in smaller sized images may be not improve visibility of staining. In the final version of the manuscript these limitations should be eliminated. Some for the images we even magnified more in this revision. The amount of images is quite big in this work, but the comparative aspect did not allow us to reduce this amount. We hope that the current version better meets the demands for both visibility and completeness of result presentation.

The way that figures 11&12 are presented only indicates whether there is a significant difference but not which direction the difference is in. It would be beneficial to either show examples of histochemical images or indicate in the current figures which of the HCN channels (fig 11) or which tonotopic region (fig12) is the largest.

The authors agree that showing examples of histochemical images would be a very valuable idea, but the restriction of space and the shortening of the text didn’t allow us to introduce more information. We didn’t find a solution to add the requested information in a simple way in the tables, but we discuss most prominent changes in the text. The authors appreciate the contribution from this reviewer to the manuscript.

Several times the authors mention a stronger labeling in Ly5.1 mice but it is not sufficiently discussed why that would be the case or if this is important.

A new statement about Ly5.1 differences compared to other mice strains was included, Why Ly5.1 present a higher intensities of the staining, could be only discussed or hypothesized.

- Page 31, lines 13-16: “Although not quantified, Ly5.1 presented the highest intensity of the staining for all the HCN subunits, suggesting that this strain is suitable for further physiological examinations. The non-myelinated SGNs also facilitated electrophysiological experiments, and the SGN anatomy was closer to the human situation.

For some statements it is unclear for which species or strain of mouse the statement is made. This leads to confusion especially when the authors wish to present a point about the general function of a certain ion channel in a certain location.

Other statements to compare and explain different results between mouse strains were also distributed throughout the text:

-Page 24, lines 19-23: “Why some animals or mouse strains present these neuron clusters frequently and others do not is unclear and will require further comparative studies. A complete lack of somatic myelination in humans favors the importance of ephaptic phase synchronization and may represent more of an evolutionary adaption than something “pathological” as suggested for the Ly5.1 mouse strain.”

-Page 27, lines 21-23: “We could not detect HCN3 in C57Bl/6N, Ly5.1, or CBA/J inbred mouse strains, so the animal model to study HCN involvement in OHC function may be crucial.”

For figure 6 it is unclear how the analysis of the fluorescence intensity contributes to quantifying colocalization of the various HCN channels.

The text was changed and we tried to explain our attempt more clearly

page 16, lines 2-8:

“Resolution was not sufficient to detect a co-localization of the different HCN subunits in a heteromeric channel configuration. However, a lack of fluorescent overlap with conventional confocal imaging techniques provided information about the presence of non-paired HCN combinations that appeared as single stained sites in double immunolabelling stainings. To assess proportional differences of HCN subunits in individual SGNs, the relative staining intensities in double staining combinations of HCN1-HCN2, HCN1-HCN4, and HCN2-HCN4 were plotted as a combined XY-histogram (Figure 6v–x).” We also removed the marginal histograms in order to simplify our presentation.

Although resolution is limited with conventional light microscopes we made the attempt to get some information of fluorescent signal co-localization within our resolution limits. To reliably unravel molecular pairing of 4 subunits with a size of ~110 kDa other techniques for structural chemistry are to be applied. The HCN 1 channel is ~100Å in length perpendicular to the membrane and ~75 Å in width when viewed from within the plane of the membrane (Lee, C. H., & MacKinnon, R. ,2017). Since best super-resolution microscopes are limited to resolve not more than 40nm a pairing of 10nm subunits is impossible to be resolved with light microscopic techniques. Anyway, our study could exclude most valid channel combinations in spiral ganglion neurons. This information may be useful for further modelling strategies and therefore we regard it as useful to be presented in a scientific journal.

Lee, C. H., & MacKinnon, R. (2017). Structures of the Human HCN1 Hyperpolarization-Activated Channel. Cell, 168(1-2), 111–120.e11. <https://doi.org/10.1016/j.cell.2016.12.023>

**Reviewer: 4**

Dear Reviewer 4,

First of all, thanks for the time and effort you spent on this manuscript. Your comments and the plenty of suggestions will help us to improve our manuscript. Recommended changes were done accordingly and addressed point by point.

Kind regards on behalf of the authors

Rudolf Glueckert & Maria Calvo

**Comments to the Author**

**The manuscript by Luque & colleagues has been resubmitted following apparent extensive changes to the text and figures. I have not seen the original submission, and so I am restricted to comments on the current version.
The study is an interesting one, that describes the distribution of HCN channel family members in the cochlea of various species (including human temporal bone samples), and mice at of several strains at various ages. As such it includes a huge volume of data, and while this will represent an important resource for the field, it is confusing in places due to the amount of descriptive data and the lack of similarities between the species/strains.
Attempts have been made to provide controls to address questions on antibody specificity. Positive controls on other tissues are suitable, as are negative controls using pre-absorption using antigenic peptides and omitting primary antibodies. Ideally, the antibodies would also be validated on knockout tissue, and on cell-lines expressing the individual channel types. These additional controls would allow the reader to appreciate how specific the antibodies are, and whether they truly distinguish between the channel family members.
At the end of the study there are attempts to relate relative expression to hearing ability. This adds to the confusion as there are no clear relationships on show. Although semi-quantitative analysis of staining intensity has been carried out, I’m not convinced that “statistical significance” can always be translated directly into “biological significance”, i.e. whether measureable differences in staining intensity for a single channel type can be seen to have influence on complex electrophysiological or behavioural outputs.**

The specificity of the antibodies was extensively studied here and in previous published work. Positive as well as negative controls were carried out in our sections with the long-term well stablish protocols in the laboratory. The same antibodies were also used before in hearing studies. We agree with the reviewer that validation in knockout tissue may be the best proof for specificity (depending also on the strategy of gene knock outs). Some global HCN knock outs are not viable anyway. Most HCN antibodies used here were KO validated by the manufacturer (listed on the Alomone website). We added this information in the text (Page 28, lines 21-23). We also added supplementary figures with some of the controls that were performed to grant transparency of the study and confirm the specific recognition of the different HCN channels subunits in different strains of mice (Supplementary figure 2, HCN4 in C57Bl/6N and CBA/J mice) and human (Supplementary figure 2, HCN2), different ages (Supplementary figure 2, HCN4 at P2, P16 and P44) and using different staining methods (Supplementary figure 2, HCN3). We added also positive control tissue to replicate known staining patterns of certain HCN subtypes.

Here we add a summary of the controls performed and literature using the same antibodies:

Positive control tissue:

Brain, cerebellum and other tissues (kidney, olfactory epithelium, liver, skeletal muscle and glands) to compare stainings with the data from literature.

Specifity- and negative controls:

A: Control peptide antigen preincubation of primaries: A preincubation of the primary antibody with the immunogenic peptide that served as epitope to produce the primary antibody in at least 20x excess shall block all specific binding sites. Any other binding that results in a positive immunostaining is unspecific.

B: Control peptide antigen preincubation with centrifugation prior slide application:

A centrifugation step for 10 minutes at 15000g after control peptide antigen preincubation was added in those cases where there was any unspecific signal after pre-incubation of the primary antibody with the control peptide antigen. Originally used for western blots to prove specificity of protein fractions, this step helps the antibody-peptide complex to settle on the sedimented fraction. We keep the supernatant that in theory should not have any antibody and discard the precipitate. Unspecific binding arises then from fractions in the antibody serum is not directed against the epitope.

C: Omitting the primary antibody: Slides were incubated with the secondary antibody without applying the primary antibody before. In all cases, the sections were negative and did not present any background staining (data not shown) that would have arisen from the secondary antibodies or visualization system.

D: isotype matched controls: In addition, substituting the primary antibodies with isotype matched controls did not give any unspecific staining, concluding that there was background in the staining produced by the primary antibody isotype and the different mammalian tissues.

E: Negative control tissue: We performed immunostainings on mouse tissues that do not express these channels (kidney, olfactory epithelium, liver, skeletal muscle and glands).

In addition, Western blot experiments were done by the manufacturer and can be seen at the allomone website (www.alomone.com). Several publications are using these antibodies too:

- guinea pig anti-HCN1 AGP-203 RRID:AB_2756625 (https://www.alomone.com/p/anti-hcn1-2/AGP-203). There is one citation using this antibody (Lopez-Gonzalez et al., 2016).

- rabbit anti-HCN2 APC-030 RRID:AB_2313726 (https://www.alomone.com/p/anti-hcn2/APC-030). There are 20 citations with this antibody, including KO validations, western blots, immunoprecipitations and immunohistochemistry experiments. For the cochlea previous publications have used it in mouse and rat tissue (Horwitz et al., 2010; Horwitz et al., 2011; Ramakrishnan et al., 2012; Shen et al., 2018).

-rabbit anti-HCN4 APC-056 RRID:AB_2039906 (https://www.alomone.com/p/anti-hcn4-2/APC-052).There are 26 citations with this antibody, including KO validations, western blots, immunoprecipitations and immunohistochemistry experiments. For the cochlea previous publications described it in rat and mouse tissue (Horwitz et al., 2011; Shen et al., 2018; Yi et al., 2010).

References:

*Bakondi, G., Por, A., Kovacs, I., Szucs, G., and Rusznak, Z., 2009, Hyperpolarization-activated, cyclic nucleotide-gated, cation non-selective channel subunit expression pattern of guinea-pig spiral ganglion cells: Neuroscience, v. 158, no. 4, p. 1469-1477.*

*Liu, Q., Manis, P. B., and Davis, R. L., 2014, I h and HCN channels in murine spiral ganglion neurons: tonotopic variation, local heterogeneity, and kinetic model: J Assoc Res Otolaryngol, v. 15, no. 4, p. 585-599.*

*Lopez-Gonzalez, Z., Ayala-Aguilera, C., Martinez-Morales, F., Galicia-Cruz, O., Salvador-Hernandez, C., Pedraza-Chaverri, J., Medeiros, M., Hernandez, A. M., and Escobar, L. I., 2016, Immunolocalization of hyperpolarization-activated cationic HCN1 and HCN3 channels in the rat nephron: regulation of HCN3 by potassium diets: Histochem Cell Biol, v. 145, no. 1, p. 25-40.*

*Horwitz, G. C., Lelli, A., Geleoc, G. S., and Holt, J. R., 2010, HCN channels are not required for mechanotransduction in sensory hair cells of the mouse inner ear: PLoS One, v. 5, no. 1, p. e8627.*

*Horwitz, G. C., Risner-Janiczek, J. R., Jones, S. M., and Holt, J. R., 2011, HCN channels expressed in the inner ear are necessary for normal balance function: J Neurosci, v. 31, no. 46, p. 16814-16825.*

*Ramakrishnan, N. A., Drescher, M. J., Khan, K. M., Hatfield, J. S., and Drescher, D. G., 2012, HCN1 and HCN2 proteins are expressed in cochlear hair cells: HCN1 can form a ternary complex with protocadherin 15 CD3 and F-actin-binding filamin A or can interact with HCN2: J Biol Chem, v. 287, no. 45, p. 37628-37646.*

*Shen, H., Liu, W., Geng, Q., Li, H., Lu, M., Liang, P., Zhang, B., Yamoah, E. N., and Lv, P., 2018, Age-Dependent Up-Regulation of HCN Channels in Spiral Ganglion Neurons Coincide With Hearing Loss in Mice: Front Aging Neurosci, v. 10, p. 353.*

*Yi, E., Roux, I., and Glowatzki, E., 2010, Dendritic HCN channels shape excitatory postsynaptic potentials at the inner hair cell afferent synapse in the mammalian cochlea: J Neurophysiol, v. 103, no. 5, p. 2532-2543.*

**Major concerns**

**1. The emphasis of antibody staining at axon initial segments is unconvincing and somewhat arbitrary. It is impossible to determine where these regions are without counterstains. A number of antibodies for nodes (e.g. Nav1.6, ankyrin G, Kv3.1), paranodes (Caspr) or juxtaparanodes (Kv1) are available to allow these specialized regions to be identified (Hossain et al, 2005; Kim & Rutherford, J Neuroscience 2016).
These parts of the manuscript should be deleted.**

New stainings were performed and added to the figures to proof staining specifically at the axon initial segments.

- Figure 1: HCN1 localization

Double stainings with CASPR 1 were performed in the C57Bl/6N mouse as kindly suggested. Colorimetric images are now included in figure 1: f, f1 and f2. CASPR 1 show specific staining and marks the distal end of axon initial segments ((Duflocq et al, 2011). HCN1 does not exceed more distal to CASPR one staining. We thoroughly screened several immunostainings and present data. These new results were incorporated in the text as well as part of the figures presenting subcellular localisation:

- Page 11, lines 20-23: results section
- Page 39, lines 10-11: Figure 1 legend

-Figure 5: HCN4 localization

Double stainings with CASPR 1 were performed in guinea pig as kindly suggested. New confocal images f, g and h show the whole central and peripheral AIS stained for HCN4 limited by CASPR 1 staining towards more distal portions of peripheral as well as central axon of bipolar auditory neurons. Images g and h show the different lengths of the AIS in guinea pig. These new results were incorporated in the text as well in pages:

- Page 15 lines 3-7: results section
- Page 42, lines 19-23: Figure 5 legend

These representative stainings provide us more certainty that HCN 1 as well as HCN4 are components of axon initial segments in auditory neurons.

**2. The attempt to relate staining intensity to hearing thresholds should be removed. The graphical representation of the staining intensities may be helpful to some readers, and they emphasise the amount of work within the study, but they are over-complicated by the overlays.**

In the first draft of the manuscript, staining intensities were presented solely in one graph. Due to the valuable suggestions from a previous reviewer we added this information also in this graphical representation. This reviewer was of the opinion that it is important to visualize the expression of HCN channels across age together with ABR thresholds in this figures. Although we already stated in the text that correlations are weak, this reviewer suggested this presents clearer in this correlative view. Visualisation of statistical significant differences in our data in the figures 11 and 12 further serve this purpose. Since this reviewer is still part of this revision we do not want to remove something that was demanded in the previous review.

**Minor concerns**

**Page 5, line 7: typo “several”** (corrected: page 5, line 8)

**Page 6, line 17: Ministry of Science** (corrected: page 6, line 13)

**Page 13, line 20: I don’t understand the statement “Tonotopical gradients are not easily determinable since even within different mouse strains and age, gradients may change (semi-quantified in Fig. 10 and 11).” Please rewrite.**

All the typo mistakes were corrected. The sentence was reworded (page 14, lines 9-10):

*“Tonotopical gradients of HCN1 expression were not static but changed among different mouse strains and within different age groups (semi-quantified in Figures 10 and 11).”*

**Page 15, line 6 & 8: typo “caliber” not caliper.** Corrected throughout all the text.

**Page 16, line 15: typo “void” not “avoid”** (corrected: page 13, line 25)

**Page 29, line 23: typo “In contrast”** (corrected: page 24, line 11)

Dear Prof Nolan,

We are grateful for the chance to have a second review of our manuscript. The authors´ regard the comments from you and the reviewers as very useful to improve the quality of the manuscript. We did our best to answer all questions from the editor and the reviewers and address all concerns adequately. We performed additional stainings as one reviewer requested and added these to the results.

Following your suggestion to correct grammar and spelling, we have sent the manuscript to the language editing service *Scribendi*. The text was considerably shortened, and some changes made to improve the readability and strengthen the take home message (changes are highlighted in the text with red font). The following table shows our attempt to shorten the manuscript listing a word count comparing this second review of the manuscript with the previous revised version (first revision). Especially the discussion was cut a lot as well as results were reduced. Material & Methods were also reduced as the editor suggested.

Please find below our point by point answers addressing you questions and concerns.

Kind regards on behalf of the authors

Rudolf Glueckert

**Associate Editor: Nolan,Lisa**

**Comments to the Author: I would like to thank the authors for their attempt to address the concerns of the reviewers and I greatly appreciate the effort to which the authors have gone to address these concerns. However, at present many ambiguous sentences and grammatical errors remain and these unfortunately make the manuscript very hard to read with clarity in places. I strongly recommend that the authors have the manuscript proof-read by somebody fluent in English Grammar and edit the manuscript aggressively before submitting any further revision to JNR. Additionally, although I appreciate that the authors have cut the text of the manuscript, further aggressive trimming of the text would also improve the readability.**

**Minor points
Age related - where it appears in the manuscript should be written as age-related.**

The text was changed accordingly (Page 2, line 8 and 15; page 5, lines 16-17; page 6, line 16; page 30, line 17; page 33, lines 6).

The following sentences were changed as suggested. The revised text and changes made to the manuscript are marked in a red font.

**Abstract

Line 9: ….single cell expression levels HCN1,2&4……
¬    The manuscript does not contain single-cell qPCR data / single-cell RNA-seq data etc; therefore, please refrain from using the term single-cell expression in the current context as it is confusing.**

Our data show quantification of HCN immunoreactivity in single cells. Since we can undoubtedly differentiate single spiral ganglion neurons in our sections and perform image segmentation based on cell type specific criteria and a following image analysis, we regard it as justified to term the analysis a “single cell analysis”. Single-cell qPCR data, single-cell RNA-seq data, etc or cell culture based methods are not the only strategies to obtain data from a single cell, especially in the complex arrangement of a tissue compound. In order to avoid confusion of terminology we change “single-cell” to “expression of individual spiral ganglion neurons” (page 9, line 9):

*“We correlated age-related hearing deterioration in CBA/J and C57Bl/6N with expression levels of HCN1, -2, and -4 in individual auditory neurons from the same cohort.”*

**Significance statement
Line 6: ….suggests HCN channels to be involved as an active component.
¬    Active component of what? Please re-phrase.**

Page 3, line 4: *“Spatiotemporal expression during postnatal development suggests its involvement as an active element in hair cell innervation”*. **Introduction
Line 6: -820 of these…
¬    Rewrite this sentence so you do not begin the sentence with 820 in numeric form as it is currently written.**

Page 4, line 6: “*From 8 to 20 of these bipolar neurons innervate a single inner hair cell (IHC)*”.

**Line 14-20
¬    These 3 aims would fit better at the very end of the introduction. They would also benefit from being written more precisely. For example: Here, we provide a detailed map of HCN channel subunit expression in the auditory system, and address whether:
i) HCN channel subunit expression in the cochlea varies among different mammalian species,ii) etc, etc.**

The introduction was re-organised and the 3 aims were moved to the end of the introduction (from page 5, lines 20-25 to page 6, lines 1-2) and rephrase as suggested:

*“With our results, we aimed to address the following questions:*

*I.)	Are there marked differences in HCN expression among mammalian species?*

*II.)	Do HCN expression levels correlate with hearing performance during ageing in mouse strains with different types of ARHL?*

*III.)	How is the spatiotemporal HCN expression pattern during mouse postnatal development associated with known data on auditory function?*

*The findings of this study shall provide a robust basis upon which to assess possible functions and better estimate HCN involvement in neuronal coding of sound.”*

**Page 43, Line 21-26 & page 44, Line 1-5**

**¬ This section would fit better before the 3 aims are listed.**

The introduction was changed and reorganized as suggested (from page 4 to 6).
 **Sex as a biological variable (SABV). Vital information on SABV is still missing or not addressed.

¬    The data from humans, cats and guinea-pigs although interesting, appears limited as the age and sex is not reported. Granted the authors have explained that this was not known for the human subjects, but what about the cats and guinea-pigs? Consequently, I am not sure the comparisons to HCN channel subunit expression in mice are helpful as it is difficult to draw clear conclusions across various species when this information is not reported / not known for the other species. If this information is not known, then please kindly address and discuss this limitation of the study in the discussion.**

Cat and guinea pig material was used in previous studies and data from the sex is not known. We have included this information in material and methods section, page 6, lines 24-25 and page 7, line 1. As we did not find any gender-related difference in the subcellular location of HCN channels among mice strains with different sexes, we did not consider sex as a variable in guinea pigs or cats.

**¬    Care should be taken to consider sex as a biological variable when performing wave l comparisons. In humans, females display larger wave l amplitudes compared to males. There is also some evidence that female mice exhibit larger wave l amplitudes compared to males. See the following papers:**

**Letter to the editor: Examination of potential sex influences in. Auditory function in normal-hearing, noise-exposed human ears, ear hear, 36, 172–184. Ear and Hearing, 36(6), 738–740.** [**https://doi.org/10.1097/AUD.00000**](https://doi.org/10.1097/AUD.00000) **00000 000228**

**The impact of biological sex on the response to noise and otoprotective therapies against acoustic injury in mice. Biology of Sex Differences, 9(1), 12.**

We agree with the editor that sex should be considered when talking about hearing loss. Due to the limitations to perform ABRs in aged mice (many die before you can do an eABR) and availability of guinea pig and cat samples we have not considered gender effects in our study. It was previously described that there are differences between male and females in very high frequencies for C57Bl/6N and at 18 months for CBA/J in models of aging hearing loss. We have already discussed previously the limitations in our study to describe these differences in these strains of mice. For this reason we have not included higher frequencies than 32 kHz in C57Bl/6N and neither mice older than 19 months in CBA/J. We are also aware that all studies listed above concentrated on models for noise induced hearing loss and compared young and old individuals.

Many causes of hearing loss that were previously described unravelled certain molecular pathologies in relation between age and noise hearing loss (Yang et al., 2015). Noise is challenging the peripheral auditory system at all ages but older individuals are often more vulnerable. Cochlear synapthophaty could be induced by noise, drugs or appear at advanced age. If they share the same mechanism is still not known (Liberman, 2017). Structural changes that compromise hearing functions are different from noise and age-related hearing loss (Kujawa and Liberman, 2019).

In our study we did not challenge the mice with noise or ototoxic drugs but intended to evaluate solely the factor ageing. Anyhow, we agree with the editor that evaluation of wave I amplitudes should have been tested for gender differences. Therefore, results about wave I amplitudes in C57Bl/6N and CBA/J were removed from this study. Figure 9 now only show the pure tone thresholds of our eABR results for the two mice strains.

**¬    The sex breakdown for the mice used in this study as given in the response to reviewers needs to be incorporated into the methods section of the paper. Ideally with the sex breakdown for the cats and guinea-pigs too – if known?**

The sex from the mice used in this study was added in the material and method section:

2.2 Auditory brainstem responses (page 7, line 20): “*A total of 77 mice (4–10 in each group) were used (28 males, 49 females) for audiometric testing.*”

2.5 Quantification of neuronal staining (page 10, lines 2-3): *“We stained sections from 37 different mice in CBA/J (22 males, 18 females) and 36 C57Bl/6N (19 males, 17 females).”*

**Additional comments**

¬ **Please keep the extra control images as supplementary data.**

Control images are kept as supplementary data (Supplementary figures 1 and 2). We reduced the number of images and concentrated on most relevant results of our control experiments.
 **Kindly mark the changes made to the text using a red and/or bold font, please do not use the red highlight.**

The changes made at the text were marked using a red font.

**As suggested/requested in the instructions to the authors kindly change the red/green images to magenta/green, so that "the work accessible to the approximately 6-10% of male readers who have red-green color blindness."**

Red-green colours were change to green-magenta in figure 9: Hearing performance in C57Bl/6N and CBA/J with age.

References:

*Kujawa, S. G., and Liberman, M. C., 2019, Translating animal models to human therapeutics in noise-induced and age-related hearing loss: Hear Res, v. 377, p. 44-52.*

*Liberman, M. C., 2017, Noise-induced and age-related hearing loss: new perspectives and potential therapies: F1000Res, v. 6, p. 927.*

*Yang, C. H., Schrepfer, T., and Schacht, J., 2015, Age-related hearing impairment and the triad of acquired hearing loss: Front Cell Neurosci, v. 9, p. 276.*

_____________________________________________________________________________________

**3^rd^ Editorial Decision	30-Sep-2020**

Dear Dr Glueckert:

Thank you for submitting your manuscript to the Journal of Neuroscience Research. We've now received the reviewer feedback and have appended those reviews below. I'm glad to say that the reviewers are overall very enthusiastic and supportive of the study, and your manuscript has been accepted pending minor changes suggested by the editor and the reviewers.

I expect that these points should be relatively straightforward to address. If there are any questions or points that are problematic, please feel free to contact me. I am glad to discuss.

We ask that you return your manuscript within 15 days. Please explain in your cover letter how you have changed the present version. If you require longer than 15 days to make the revisions, please contact Dr Cristina Ghiani (cghiani@mednet.ucla.edu). To submit your revised manuscript: Log in by clicking on the link below

(If the above link space is blank, it is because you submitted your original manuscript through our old submission site. Therefore, to return your revision, please go to our new submission site here (submission.wiley.com/jnr) and submit your revision as a new manuscript; answer yes to the question “Are you returning a revision for a manuscript originally submitted to our former submission site (ScholarOne Manuscripts)? If you indicate yes, please enter your original manuscript’s Manuscript ID number in the space below” and including your original submission's Manuscript ID number (jnr-2020-Jan-8540.R2) where indicated. This will help us to link your revision to your original submission.)

Thank you again for your submission to the Journal of Neuroscience Research; we look forward to reading your revised manuscript.

Best Wishes,

Dr Lisa Nolan

Associate Editor, Journal of Neuroscience Research

Dr Cristina Ghiani

Editor-in-Chief, Journal of Neuroscience Research

Associate Editor: Nolan, Lisa

Comments to the Author:

The authors have done a comprehensive job addressing all concerns raised by the reviewers and I appreciate the further trimming and structuring of the manuscript. The study makes for a very nice, novel contribution to the auditory field. I recommend this manuscript for publication in JNR.

Outstanding point.

Not all figures have yet been amended to comply with JNR's policy on colour blindness.

Reviewer: 4

Comments to the Author

The manuscript is much improved following the extensive re-write and pruning.

The additional experiments have clarified some elements of the story.

In the Significance Statement I suggest a change of wording: "Lateral membrane staining in outer hair cells suggests association with cochlear amplification".

Reviewer: 3

Comments to the Author

The authors have adequately responded to the comments of the reviewers.

This is a comprehensive study on HCN channel expression/function in the inner ear that will make a significant contribution to the field.

*********IMPORTANT: Instructions and checklists follow*********

When finalized, please upload your complete revised manuscript onto our website, preferably as a word document. Please ensure to upload a highlighted version of your manuscript along with the clean version. The highlighted version should highlight the revised text or any other changes made to the manuscript. The clean version should have no highlighted sentences, strike-through words, or comments in margins. Kindly avoid submitting a document with tracked changes. Figures must be uploaded separately in .tif or eps format. Please review our submission checklist, which can be found in our author guidelines and also be sure to fill out the Transparent Science Questionnaire attached to this email.

JNR offers Open Science badges to qualifying authors. For more information please see the “Open Science initiatives” section of our author guidelines. If you would like to apply for one or more of the badges, please complete the included disclosure form and upload it as Supplemental Material Not for Review when submitting your final manuscript files.

If you encounter any troubles in submitting your revised manuscript, please contact Dr Cristina Ghiani

_____________________________________________________________________________________

**Authors’ Response	08-Oct-2020**

Innsbruck, 08.10.2020

Dear Prof Nolan,

We are very grateful and happy to have our manuscript accepted in this outstanding and well established journal in the field of neuroscience.

The authors´ regard the comments from you and the reviewers as a big help to improve our manuscript. It was a challenge to present our plentiful results in an understandable way for the reader and we very much appreciated the guidance from the editor and reviewers. As the editor pointed out, the graphical abstract figure did not comply with JNR's policy on colour blindness. Red colour was therefore changed now to magenta and the new figure uploaded. Minor changes were done to the document and are highlighted in red. The suggestion from reviewer 4 was added into the significance section in the text. Some figure labelling required corrections, and replaced on the journal website, as well as the tables due to minor spelling corrections and style shaping.

If you have further questions, comments or suggestions do not hesitate to contact us.

Kind regards on behalf of the authors,

Rudolf Glueckert & Maria Luque Calvo

**4^th^	Editorial Decision	12-Oct-2020**

Dear Dr Glueckert and Dr Calvo:

Thank-you for adjusting the graphical abstract as per JNR author guidelines on Red-Green colour blindness. However, the confocal images have not been adjusted.

The author guidelines for JNR request that confocal figures for red-green colour blindness are amended to magenta-green or magenta-green versions can be uploaded as supporting information. I have attached the specific instructions as detailed on JNR's website and have listed the figures in question below, kindly, change the following panels to magenta-green.

Fig. 1b, 1c

Fig. 2b; 2b1; 2b2

Fig. 5f; 5f1; 5f2; 5g; 5h; 5h1; 5h2

Fig. 6a, 6b, 6c, 6d, 6h, 6i, 6j, 6k, 6o, 6p, 6q, 6r

We ask that you return your manuscript within 15 days. Please explain in your cover letter how you have changed the present version. If you require longer than 15 days to make the revisions, please contact Dr Cristina Ghiani (cghiani@mednet.ucla.edu). To submit your revised manuscript: Log in by clicking on the link below

(If the above link space is blank, it is because you submitted your original manuscript through our old submission site. Therefore, to return your revision, please go to our new submission site here (submission.wiley.com/jnr) and submit your revision as a new manuscript; answer yes to the question “Are you returning a revision for a manuscript originally submitted to our former submission site (ScholarOne Manuscripts)? If you indicate yes, please enter your original manuscript’s Manuscript ID number in the space below” and including your original submission's Manuscript ID number (jnr-2020-Jan-8540.R3) where indicated. This will help us to link your revision to your original submission.)

Thank you again for your submission to the Journal of Neuroscience Research; we look forward to reading your revised manuscript.

Best Wishes,

Dr Lisa Nolan

Associate Editor, Journal of Neuroscience Research

Dr Cristina Ghiani

Editor-in-Chief, Journal of Neuroscience Research

*********IMPORTANT: Instructions and checklists follow*********

When finalized, please upload your complete revised manuscript onto our website, preferably as a word document. Please ensure to upload a highlighted version of your manuscript along with the clean version. The highlighted version should highlight the revised text or any other changes made to the manuscript. The clean version should have no highlighted sentences, strike-through words, or comments in margins. Kindly avoid submitting a document with tracked changes. Figures must be uploaded separately in .tif or eps format. Please review our submission checklist, which can be found in our author guidelines and also be sure to fill out the Transparent Science Questionnaire attached to this email.

JNR offers Open Science badges to qualifying authors. For more information please see the “Open Science initiatives” section of our author guidelines. If you would like to apply for one or more of the badges, please complete the included disclosure form and upload it as Supplemental Material Not for Review when submitting your final manuscript files.

If you encounter any troubles in submitting your revised manuscript, please contact Dr Cristina Ghiani ([cghiani@mednet.ucla.edu](mailto:cghiani@mednet.ucla.edu)).

_____________________________________________________________________________________

**Authors’ Response	20-Oct-2020**

Dear Prof Nolan,

We are very grateful again and happy to have our manuscript accepted in this reputed journal.

As the editor advised, all figures containing a double labelling presented as red-green confocal microscopy images were changed to magenta-green. New figures are now uploaded to the journal website. The content in the figures was not modified, but only the colour red was exchanged by magenta and the document updated accordingly. This minor modifications are highlighted in red.

If you have further questions, comments or suggestions do not hesitate to contact us.

Kind regards on behalf of the authors,

Rudolf Glueckert & Maria Luque Calvo

**5^th^ Editorial Decision	22-Oct-2020**

Dear Dr Glueckert:

You will be pleased to know that your manuscript has been accepted for publication. Thank you for submitting this excellent work to our journal.

In the coming weeks, the Production Department will contact you regarding a copyright transfer agreement and they will then send an electronic proof file of your article to you for your review and approval.

Please note that your article cannot be published until the publisher has received the appropriate signed license agreement. Within the next few days, the corresponding author will receive an email from Wiley’s Author Services asking them to log in. There, they will be presented with the appropriate license for completion. Additional information can be found at https://authorservices.wiley.com/author-resources/Journal-Authors/licensing-open-access/index.html

Would you be interested in publishing your proven experimental method as a detailed step-by-step protocol? Current Protocols in Neuroscience welcomes proposals from prospective authors to disseminate their experimental methodology in the rapidly evolving field of neuroscience. Please submit your proposal here: https://currentprotocols.onlinelibrary.wiley.com/hub/submitaproposal

Congratulations on your results, and thank you for choosing the Journal of Neuroscience Research for publishing your work. I hope you will consider us for the publication of your future manuscripts.

Sincerely,

Dr Lisa Nolan

Associate Editor, Journal of Neuroscience Research

Dr Cristina Ghiani

Co-Editor-in-Chief, Journal of Neuroscience Research

Associate Editor: Nolan, Lisa

Comments to the Author:

The authors have now amended the confocal figures in-line with JNR's policy on red-green colorblindness. I have no further queries and look forward to seeing the manuscript published in JNR.

NIH GRANTEES: All NIH authors should also be aware that Wiley-Blackwell will submit manuscripts to the NIH Manuscript Submission system automatically. After their accepted manuscript files have been posted by Wiley-Blackwell to the NIH Manuscript Submission system, the corresponding author will receive an e-mail request from PubMed Central asking them to approve the upload for display on the PubMed Central system. Authors will need to provide approval to complete the process of deposition to PubMed Central. This is a requirement of their grant/affiliation.

P.S. You can help your research get the attention it deserves! Check out Wiley’s Promotion Guide for best-practice recommendations for promoting your work at www.wileyauthors.com/maximize

_____________________________________________________________________________________
